# Quantitative proteomic analysis of histone modifications in decitabine sensitive and resistant leukemia cell lines

**DOI:** 10.1186/s12014-016-9115-z

**Published:** 2016-07-05

**Authors:** Chunchao Zhang, Jinfeng Suo, Hiroyuki Katayama, Yue Wei, Guillermo Garcia-Manero, Samir Hanash

**Affiliations:** Clinical Cancer Prevention, University of Texas MD Anderson Cancer Center, 6767 Bertner Ave, Houston, TX 77030 USA; Department of Leukemia, University of Texas MD Anderson Cancer Center, 1515 Holcombe Boulevard, Unit 428, Houston, TX 77030 USA

**Keywords:** Leukemia, Acute myeloid leukemia (AML), Myelodysplastic syndromes (MDS), Decitabine (DAC), Histone modifications, Mass spectrometry

## Abstract

**Background:**

The refractory nature of many cancers remains the main health challenge over the past century. The epigenetic drug, decitabine (DAC), represents one of the most promising therapeutic agents in cancers particularly in acute myeloid leukemia (AML) and myelodysplastic syndromes (MDS). However, its ambiguous anti-tumor mechanism and the unpredictable drug-resistant nature in some population compromise its application in cancer therapy. In crosstalk with DNA methylation, histone post-translational modifications (PTMs) are the key players in modulating the downstream epigenetic status of tumor suppressor genes. This study targets the role of decitabine in epigenetic regulation in leukemia therapy and searches responsive predictors and therapeutic targets for pretreatment evaluation and drug development.

**Results:**

A simple, fast, and robust proteomic strategy identified 15 novel PTMs and 60 PTM combinations in two leukemia cell lines (MDS-L and TF-1). Histone modification profiles have been generated and compared between DAC sensitive and resistant groups (n = 3) in response to DAC treatment. Among these histone PTMs, five of which were found differentially upon DAC treatment in drug sensitive and resistant cells: H3.3K36me3, H4K8acK12acK16ac in MDS-L cells; and H3.1K27me1, H3.1K36me1, H3.1K27me1K36me1 in TF-1 cells. They may serve as biomarkers in predicting leukemia and drug responsiveness. In addition, we also explored PTM differences in two cell lines which were developed from early and advanced stages of AML. Three PTMs (H3.1K27me3, H3.1K27me2K36me2 and H3.3K27me2K36me2) are highly abundant in TF-1 cells (advanced AML cell line), suggesting their relevance to leukemogenesis. Our method allowed deep analysis of histone proteins and elucidation of a large number of histone PTMs with high precision and sensitivity.

**Conclusion:**

DAC-induced DNA hypomethylation has wide impact on chromatin modifications. This study represents first effort to investigate the undefined epigenetic mechanism of decitabine in leukemia therapy. The identification of 15 novel PTMs and the discovery of several marks have relevance to epigenetic directed therapies.

**Electronic supplementary material:**

The online version of this article (doi:10.1186/s12014-016-9115-z) contains supplementary material, which is available to authorized users.

## Background

Epigenetics is an active field of cancer research. DNA methylation and histone modifications are two major contributors to epigenetics. As a crucial epigenetic mark on the genome, DNA methylation modulates many important cellular processes including embryonic development, transcription, mammalian X chromosome inactivation, genomic imprinting and chromosome stability [1, 2]. About 60–80 % of CpGs in the human genome undergo DNA methylation [3]. There are two classes of DNA methyltransferases (DNMTs) that catalyze the transfer of the methyl group onto DNA: (1) DNMT1 maintains the global methylation pattern; and (2) DNMT3A and DNMT3B perform de novo methylation during embryonic development [3–7]. DNA methylation is generally considered a repressive mark and is often associated with gene silencing [1, 8]. The balance between DNA methylation and demethylation is required to be precisely maintained and dysregulation of the balance may lead to human diseases notably cancer [3, 9, 10]. Global DNA hypomethylation as well as DNA hypermethylation in the promoter regions of tumor suppressor genes are common hallmarks of cancer cells [10–12].

Histone modifications, on the other hand, provide another layer in modulating DNA replication and gene transcription. Histones are relatively small proteins with high content of basic residues, lysine and arginine, making these proteins strongly positively charged which contributes to their tight interactions with the negatively charged DNA backbone [13]. There are two types of histones: (1) Linker histone H1; and (2) Core histones (H2A, H2B, H3, and H4) [14, 15]. A protein octamer formed by two of each copy of core histones constitutes the basic unit of eukaryotic chromatin [13, 14]. Both linker histones and core histones undergo a large number of chemical modifications including methylation, acetylation, phosphorylation, biotinylation, citrullination, ADP-ribosylation, and ubiquitination [16]. Histone modifying enzymes are responsible for addition or removal of these different types of chemical modifications [13, 17, 18]. A wide range of dynamic histone marks (histone code), their modifying enzymes (code writers or erasers), and their downstream effector molecules (code readers) are key players in regulating eukaryotic chromatin structure and functions [19–22]. Multiple regulatory layers can be achieved by changing the levels, types, and positions of PTMs on these proteins. As with DNA methylation, a wrong histone code (i.e. an aberrant histone modification pattern) written or erased by histone modifying enzymes may result in disease [23–26].

As DNA and histones are physically intertwined, changes in chromatin states often require synergistic actions affecting both DNA methylation and histone modifications involving proteins including DNMTs, histone modifying enzymes, and chromatin binding proteins [27–29]. Little is known, however, about the crosstalk between these two types of modifications in many diseases. Some preliminary studies have pointed to a connection between these two types of modifications and their relevance to human cancers [8, 11, 28]. DNA methylation may affect histone modification patterns and altered histone modifications also impact the DNA methylome and vice versa [30–37]. Therefore, all of these marks and proteins mentioned above provide a rich source to mine for cancer biomarkers and therapeutic targets.

The recent advancements in cancer epigenetics has led to the development of new epigenetic drugs, most of which are DNA methyltransferase inhibitors (DMNTi) or histone modifying enzyme inhibitors (e.g. HATi, HDACi, HMTi, and HDMi) [38–42]. Decitabine (DAC), a DNA methylation inhibitor, has potential as a therapy for myelodysplastic syndromes (MDS) and certain types of leukemia [43–48]. DAC depletes DNMT1 and reverses aberrant epigenetic repression of tumor suppressor genes via an unknown mechanism. Low-dose administration of DAC is less toxic and improves efficacy in MDS and other cancers [49–52]. On the other hand, ~50 % of patients are non-responders and most of patients eventually develop resistance to the drug [45]. Patients who are resistant to DAC have limited alternative options and have high mortality. The ambiguous anti-tumor mechanism and unpredictable basis for drug-resistance represent a current challenge. Therefore, knowledge of DAC anti-tumor effects and mechanisms of resistance has essential relevance to cancer therapy.

Liquid chromatography tandem mass spectrometry (LC–MS/MS) technique has become the most popular and powerful tool for large-scale protein identification, PTM analysis, protein–protein interactions, and etc. [53, 54]. The quantitative feature gives it extra credit in comparing thousands of components across multiple samples in different cell states [55–57]. However, analysis of histones and their posttranslational modifications are challenging owing the high complexity and combinatory manners of the numerous chemical modifications, which often involves the multi-step histone purifications such as HPLC and gel electrophoresis [58–60]. As a result a large number of cells or tissues are required to obtain enough histones for mass spectrometry analysis, complicating analysis of clinical samples available in limited quantities. Since histone proteins have excess basic amino acid residues (lysine and arginine), trypsin digestion will generate undersized, hydrophilic peptides which are unfavorable when conventional proteomics strategy is adopted. Chemical derivation of histones, such as propionylation [61], is a popular protocol which greatly facilitates LC–MS/MS analysis by introducing an artificial modification (propionyl group) onto lysine residues generating longer, more hydrophobic tryptic peptides. However, operational variations during sample preparation are introduced since propionylation of histones is usually incomplete and varies from batch to batch [62]. For comparison of histone PTMs in different biological states, chemical or metabolic labeling techniques often provide more accurate quantification than label-free methods which require more measurements and repeats to achieve the power of statistical tests [63–65]. Although middle-down analysis of large-size peptides (3–9 kDa) or top-down analysis of intact histones gives more comprehensive assessment of the histone code, the use of these approaches is largely limited since they require more separation steps and large amounts of histones [66]. Targeted proteomics allows absolute quantification of histone PTMs by spiking samples with synthetic, isotope labeled peptides [67]. Nonetheless, this strategy is designed upon biomarker validation not for discovery purpose. To dissect the role of DAC in epigenetic regulation in leukemia therapy, we have implemented a simple, fast, highly sensitive proteomic method for mapping and quantifying histone modifications and their combination patterns in leukemia cells based on high-resolution mass spectrometry. Prior to enzyme digestion and LC–MS/MS analysis, this method relies on a simple histone enrichment step without HPLC or gel separation, thus greatly simplifying the overall workflow. Propionylation in combination with stable isotope labeled histones as internal standard further improves the PTM identification and quantification. Using this strategy, we have identified 61 individual histone marks and quantified the relative levels of 60 PTM combinations in two leukemia cell lines. We also investigated effects of treatment with thus linking DNA methylation and chromatin modifications.

## Methods

### Cell culture and experimental design

**Fig. 1:**
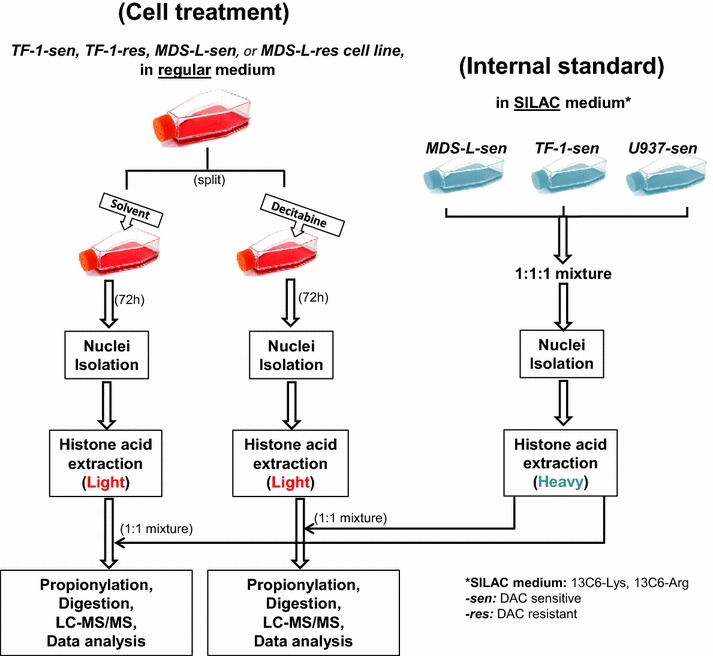
Experimental design. To reveal DAC-resistant mechanism, parental TF-1 and MDS-L cell lines were selected and their drug-resistant cell lines were developed. Parental and DAC-resistant cells were cultured in RPMI-1640 medium with or without drug treatment. The internal standard is composed of three cell lines (parental MDS-L, TF-1, U937) which were cultured in SILAC medium (^13^C_6_-Lys, ^13^C_6_-Arg) and equally mixed before nuclear isolation and acid extraction. Cells were collected and underwent nuclear isolation and acid extraction to achieve crude histone mixture. The light and heavy extracts were mixed equally followed by propionylation, trypsinization, LC–MS/MS and data analysis

Two cell lines, TF-1 (derived from human erythroleukemia, purchased from ATCC) and MDS-L (derived from patient MDS) were chosen for this study. TF-1 cells were maintained in RPMI-1640 containing 10 % fetal bovine serum and 10 ng/mL interleukin 3. MDS-L cells were maintained in RPMI-1640 containing 20 % fetal bovine serum and 10 ng/mL interleukin 3. An additional cell line U937 was maintained in RPMI-1640 medium containing 10 % fetal bovine serum. To develop resistance to DAC, parental (DAC-sensitive) TF-1 and MDS-L cell lines were challenged with decitabine over a year. Both DAC-sensitive and DAC-resistant cell lines were then cultured in DAC-free medium and analyzed with or without treatment with DAC (1-3 µM) for 72 h in the same medium. To obtain the global internal standard, the three parental cell lines (MDS-L, TF-1, U937) were cultured in SILAC medium (R1780 SIGMA, RPMI-1640 Medium) supplemented with heavy amino acids-l-ARGININE: HCL (13C6, 99 %, CLM-2265-H-0.5) and l-LYSINE: 2HCL (U-13C6, 99 %; CLM-2247-H-0.5) purchased from Cambridge Isotope Laboratories. After SILAC labeling, they were mixed in equal numbers prior to nuclear isolation and histone acid extraction. Meanwhile, regularly cultured (unlabeled) cell lines also underwent nuclear isolation and acid extraction separately (Fig. 1).

### Nuclear isolation and bulk histone acid extraction

About 1 × 10^6^ cells were collected by centrifugation and washed twice with 10 mL ice-cold PBS (Phosphate-buffered saline) supplemented with 5 mM Sodium Butyrate (Sigma-Aldrich). Cell pellets were suspended in 1 mL ice-cold TEB Buffer (Triton Extraction Buffer: PBS containing 0.5 % Triton X 100 (v/v), 2 mM PMSF) supplemented with 5 mM Sodium Butyrate, 1× Protease inhibitor (Roche), 1× PhoStop (Roche), and incubated in a rotator at 4 °C for 10 min. Intact nuclei released from cells were collected by spinning at 3000*g* for 10 min at 4 °C. Nuclei were washed with 1 mL TEB buffer and spun down again. For histone acid extraction, nuclear pellets were resuspended in 1 mL of 0.4 N H_2_SO_4_ with gentle spin in a rotator overnight at 4 °C. Nuclear debris was removed by centrifugation at 16,000*g* for 10 min at 4 °C in a cooled table-top centrifuge. The supernatant containing bulk histones was transferred into a new Eppendorf tube for precipitation by addition of TCA (Trichloroacetic acid, Sigma-Aldrich) drop by drop to the histone supernatant (final concentration of TCA = 33 %). Histones were collected by spinning at 16,000*g*, for 10 min at 4 °C. Pellets containing histones were washed with ice-cold acetone twice. Once acetone was removed, pellets were air dried for 20 min at room temperature, dissolved in 400 µL H_2_O, and stored at −20 °C. Both linker histone and core histones could be effectively enriched after this simple step. Typically, more than 100 µg proteins can be extracted from 1 × 10^6^ cells and up to 80 % of the proteins are histones. Purity of crude histones after acid extraction was checked by means of SDS-PAGE (Sodium dodecyl sulfate polyacrylamide gel electrophoresis) and gel staining with Coomassie Brilliant Blue.

### Chemical derivatization, trypsinization, and LC–MS/MS

Unlabeled bulk histones extracted from TF-1 or MDS-L (sensitive or resistant cells treated with or without DAC) were equally mixed with SILAC labeled crude histones extracted from a three cell line mixture which serves as internal standard for comparison across samples collected under different conditions (Fig. 1). The light and heavy protein mixture was chemically propionylated twice before and after trypsin digestion as previously described [61, 68]. Propionylation significantly increases the hydrophobicity and retention times of tryptic peptides on a reversed-phase column, which improves the number of PTMs identified and quantified by LC–MS/MS. Samples were then mixed in 100 mM ammonium bicarbonate buffer and digested with sequencing-grade trypsin (Promega, Madison, WI) at a ratio of 1:20 (enzyme:substrate) at 37 °C for 6 h. The digested mixture was vacuum-dried, reconstituted in 0.1 % formic acid and filtered by 10 kDa cut-off centrifugal filter unit (Millipore Ultracel YM-10). About ~1 μg of protein digest was loaded onto a C18 trap column (Waters, 180 μm ID × 20 mL) and resolved on a 25 cm long, capillary column (75 μm ID × 360 μm OD, New Objective) packed with 5 μm, 200 Å C18 silica-bonded material (Magic C18 AQ, New Objective). Peptides were eluted using a linear gradient as follows: run 100 % solvent A (0.1 % formic acid) over 5 min; run a 0–40 % gradient against solvent B (0.1 % formic acid in acetonitrile) over 90 min; and finally run 10 min, 95 % solvent B at a flow rate of 300 nL/min. The eluted peptides were analyzed using a Thermo Fisher Q Exactive Hybrid Quadrupole-Orbitrap Mass Spectrometer under data dependent acquisition mode. After a full scan (m/z 300–1800), tandem spectra were collected by selecting the 10 most intensive peaks with normalized collision energy of 35 %. Resolution on MS1 and MS2 was 70,000 and 17,500 respectively. A dynamic exclusion of 35 s and internal mass lock (445.12002 m/z) were also executed to obtain best performance.

### Data analysis

A quantitative proteomics software package MaxQuant (version 1.5.2.8) was used for raw data processing, peptide identification, and quantification [69]. Collected spectra were searched against Uniprot Human proteome FASTA database (released in April 2015) containing 90,411 sequences. Mass tolerance for precursor ion was 10 and 20 ppm for the fragment ions. N-terminal propionylation was considered as fixed modification while lysine acetylation, lysine and arginine methylation, and lysine propionylation were searched as variable modifications. Up to five missed cleavages were allowed during digestion as trypsin doesn’t cleave propionylated lysine. Data were normalized so that the median of the logarithms of all peptide ratios in each LC–MS run is zero [69]. We accepted 1 % false discovery rate at both peptide and protein level estimated from decoyed sequences. All spectra identified with PTMs were manually checked and validated. The peptide ratios of light/heavy were evaluated in biological triplicates. The true ratios of peptides and proteins in any two different conditions can be directly converted from their light/heavy ratios. Unpaired, two-tailed Student’s *t* test was analyzed in Microsoft Excel.

## Results and discussion

### Research strategy and method development

Owing to the challenges and difficulties in analyzing histone PTMs (discussed in “Background” section), we have developed a simple, fast, and robust quantitative LC–MS/MS proteomic method. Compared to the existing methods, our entire experimental platform has several advantageous features for confident PTM identification and quantification in human cells: (1) The simple enrichment procedure with acid extraction effectively isolated bulk histones containing both linker histone and core histones with some minor non-histone contaminants, which greatly reduces the workload, and improves sensitivity, throughput and reproducibility. The whole enrichment procedure took less than 1 day before trypsin digestion and LC–MS/MS analysis. (2) In comparison to label-free quantification, the use of internal standard derived from three SILAC-labeled cell lines reduces sample sizes and gives a better PTM coverage owing to PTM pattern varies from cell line to cell line. In comparison to multiplex SILAC labeling, our protocol is more cost-effective and also allows longitudinal study of histone modifications in clinical samples. (3) The chemical derivatization took advantage of the fact that propionylation blocks trypsin cleavage at lysine and neutralizes positive charges on lysine residues [61], thus generates adequately sized, more hydrophobic peptides suitable for reverse-phase mass spectrometry analysis. The variations caused by incomplete propionylation during sample preparation are greatly minimized by using the internal standard which enables reliable and consistent comparison of protein and PTM abundance in different samples [62]. (4) The use of Q Exactive mass spectrometers that offer high-resolution option on both MS1 and MS2, allows for high confidence identification of peptides and PTMs. All together, these efforts greatly improve the number of PTMs identified and quantified by LC–MS/MS [63, 68, 70, 71]. Our optimized method allowed profiling of histones and their PTMs from as few as 10^5^ leukemia cells.

In total, 22 histones or histone variants were identified with at least one unique peptide (Additional file 1: Table S1) and 108 modified peptide species (Additional file 2: Table S3) consisting of 61 distinct histone marks at 39 sites in both linker and core histones (Additional file 3: Figure S1, Additional file 4: Figure S2). Importantly, conserved histone PTMs were detected and quantified in this study. Among these marks, 15 are novel PTMs (Additional file 3: Figure S1) according to the UniProt Knowledgebase. They are: H1.0 Lys-40 mono-methylation, H1.2 Lys-52 (or H1.3 Lys-53, H1.4 Lys-52) mono-methylation, H1.5 Lys-55 mono-methylation, H1.5 Lys-78 mono-methylation, H1.4 Lys-119 (or H1.5 Lys-122) mono-methylation, H1.4 Lys-121 (or H1.5 Lys-124) mono-methylation, H1.2/H1.5 Lys-172 mono-methylation, H2A type 1-D Lys-43 mono-methylation, H2A.1/H2A type 1-C/H2A type 1-D Lys-126 mono-methylation, H2B type 2-F Lys-21 mono-methylation, H2B type F-S Lys-25 mono-methylation, H2B type F-S Lys-29 mono-methylation, H4 Lys-9 mono-methylation, H4 Arg-56 mono-methylation, and H4 Lys-78 mono-methylation. In addition, 40 individual PTMs or 60 PTM combinations were quantified in leukemia sensitive and resistant cell lines (Additional file 5: Table S2). After normalization, most proteins and PTMs quantified had Light/Heavy ratios close to one with small statistical variations (Fig. 3; Additional file 1: Table S1, Additional file 5: Table S2) suggesting reliability of the method.

### PTMs related to decitabine resistance

As our primary effort, we first targeted the DAC resistance in MDS-L and TF-1 cells which represent early and late stage of the disease respectively. To identify PTMs that are related to DAC resistance, we investigated histone modification profiles in DAC-sensitive and DAC-resistant cells upon drug treatment.

**Fig. 2:**
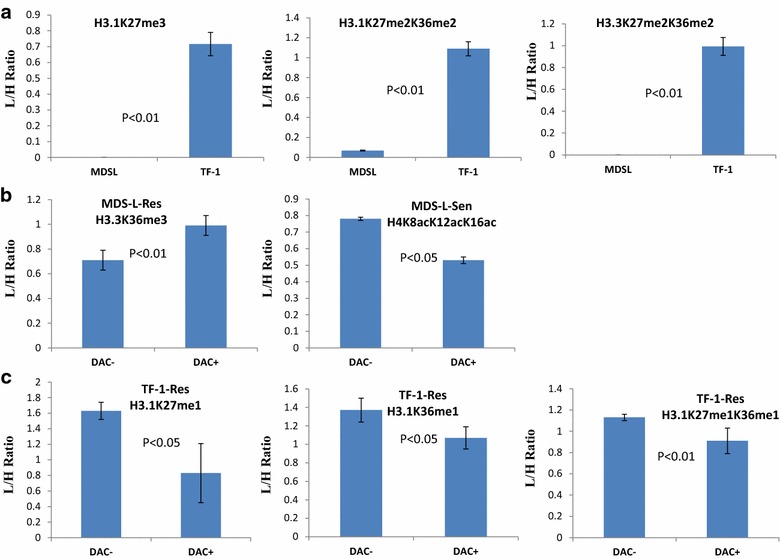
Differentially expressed PTMs in different groups. **a** 3 PTMs as signature in the TF-1 cells. H3.1K27me3 and H3.3K27me2K36me2 are only detectable in the TF-1 groups while H3.1K27me2K36me2 presents at a much lower level in MDS-L cells (0.07 vs 1.06, p < 0.01); **b** Induction of H3.3K36me3 and reduction of H4K8acK12acK16ac in MDS-L cells after DAC treatment; **c** Reduction of mono-methylation states on H3 Lys-27 and Lys-36 in the DAC-resistant TF-1 cells after drug stimulus. H3.1K27me1 is significantly lower in the resistant cells after DAC treatment (0.51 vs 1.08, p < 0.05). H3.1K36me1 and H3.1K27me1K36me1 are moderately decreased in the resistant cells after drug exposure. No significant changes found in the sensitive cells in response to the drug stimulus

**Fig. 3:**
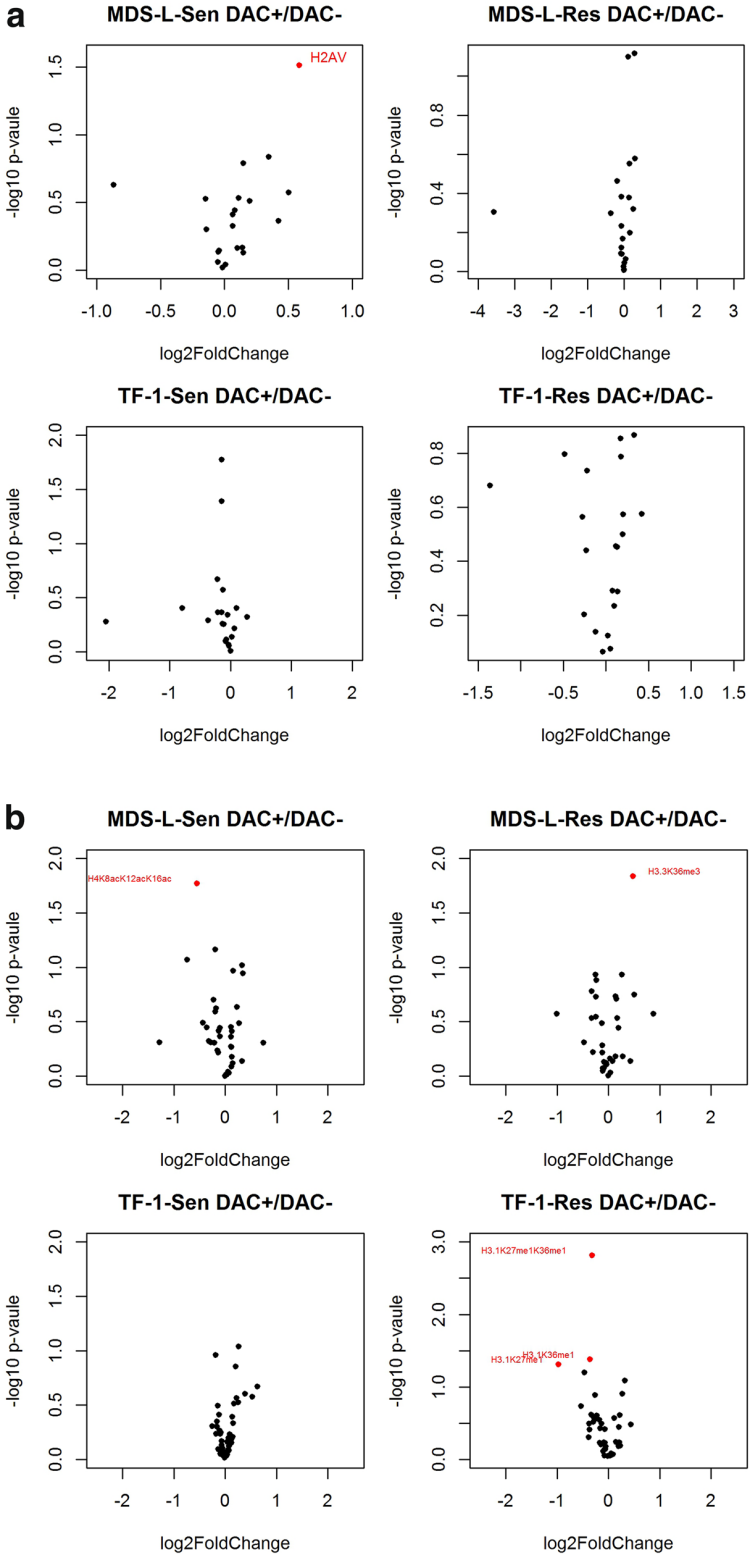
Fold changes of histones and histone PTMs in two cell lines. **a** Volcano plot of protein fold changes in sensitive and resistant cells before and after DAC treatment. **b** Volcano plot of PTM changes in sensitive and resistant cells before and after DAC treatment. *Sen*, sensitive cells; *Res*, resistant cells; DAC−, no drug treatment; DAC+, drug treated

In MDS-L cells, two peptide species (Additional file 5: Table S2, Figs. 2, 3b) showed significantly difference among sensitive and resistant groups in response to DAC treatment (p < 0.05): 27-KSAPSTGGV*K*me3KPHR-40 (H3.3K36me3, Additional file 6: Figure S3. Note: N-terminal methionine is not counted in the following text) and 4-GKGG*K*acGLG*K*acGGA*K*acR-17 (H4K8acK12acK16ac, Additional file 6: Figure S3). H3.3K36me3 was moderately induced in MDS-L resistant cells after DAC treatment (final ratio = 1.39, p < 0.01) as compared to sensitive cells (final ratio = 1.09). Whereas tri-acetylation on the H4 N-terminal tail (H4K8acK12acK16ac) was reduced after DAC treatment in MDS-L sensitive cells (final ratio: 0.68, p < 0.05) but this modification remained mostly unchanged in resistant cells upon drug exposure (final ratio: 0.93). Alteration of histone lysine methylation and their enzymes are often associated with leukemogenesis or tumor progression and drugs targeting disordered patterns of histone methylation are being developed [72]. On the other hand, deregulation of histone acetylation and their modifying enzymes (HATs and HDACs) are common observations in AML and MDS [73]. H4K8acK12acK16ac may be relevant to the development of inhibitors of HAT and HDAC as in the case of Vorinostat and Romidepsin [38].

In TF-1 cells, three mono-methylation states on H3 Lys-27, Lys-36, and Lys-37 changed differently among sensitive and resistant groups in response to DAC treatment (Figs. 2, 3b). 27-*K*me1SAPATGGVKKPHR-40 (H3.1K27me1, Additional file 6: Figure S3) was reduced by about 50 % (final ratio = 0.51, p < 0.05) in resistant cells while this modification maintained stable in TF-1 sensitive cells (final ratio = 1.08). The other two modification states: 27-KSAPATGGV*K*me1KPHR-40 (H3.1K36me1, Additional file 6: Figure S3) and 27-*K*me1SAPATGGV*K*me1KPHR-40 (H3.1K27me1K36me1, Additional file 6: Figure S3) were moderately decreased in resistant cells after drug treatment. These methylation states were also stable in sensitive cells in response to the drug treatment. EZH2, one of the H3K27 methyltransferases and the principal component of Polycomb Repressive Complex 2 (PRC2), is a key player (code writer) in regulating the methylation states of Lys-27 in histone H3 [74]. EZH2 also controls DNA methylation by interacting with DNMTs [75]. Given its key function in maintaining gene transcription, mutation or deregulation of EZH2 is associated with cancer development [74]. Development of inhibitors of EZH2 represents a therapeutic strategy for cancer [76]. Our study has provided additional evidence that H3K27 methylation may play a role in DAC treatment effect. However, no common individual marks related to drug resistance were found in both cell lines. This may be explained by cell line difference or disease stages.

### Highly abundant PTMs in TF-1 cells

As our secondary effort, we investigated and compared the PTM patterns in these two cell lines since alterations of epigenetic marks may be related to disease stages. The TF-1 cell line was first established from a patient with erythroleukemia, a rare form of acute myeloid leukemia (AML) [77]. The MDS-L cell line was derived from myelodysplastic cells [78]. MDS could transform into AML thus is generally regarded as a pre-cancer stage of AML [78]. Therefore, studying these clinically closely related cell lines could provide information about leukemia development and progression.

By comparing steady-state level of histone PTMs in the sensitive cell lines, we found that di- and tri-methylation states of H3K27 were significantly more abundant in TF-1 cells compared to MDS-L cells (Fig. 2). Three species were identified in total consisting of 27-*K*me3SAPATGGVK-36 (H3.1K27me3, Additional file 6: Figure S3), 27-*K*me2SAPATGGV*K*me2KPHR-40 (H3.1K27me2K36me2, Additional file 6: Figure S3), and 27-*K*me2SAPSTGGV*K*me2KPHR-40 (H3.3K27me2K36me2, Additional file 6: Figure S3) that were substantially more abundant in TF-1 or missing in MDS-L (Additional file 5: Table S2). H3.1K27me3 and H3.3K27me2K36me2 were identified in all samples from the TF-1 cell line but were not detectable in all MDS-L cell line samples. H3.1K27me2K36me2 were found in all TF-1 samples but were only detected in two MDS-L samples with very low Light/Heavy ratios (0.068 on average). Of note the dimethylation state of H3K27 (27-*K*me2SAPATGGVK-36) was also significantly reduced in MDS-L cells compared to TF1 cells (Light/Heavy ratio: 0.23 vs 1.61, p < 0.01).

The role of H3K27 methylation has been extensively studied and a precise balance of this modification is a key to maintaining normal cell growth [79]. There are several histone methyltransferases that impact H3K27 methylation including EZH1, EZH2, and WHSC1LI [80]. Moreover, there are also three histone demethylases that reverse the methylation status of H3K27 [81]. Mutations of these chromatin modifiers are often linked to cancer [82]. H3K27 methylation is generally considered as a repressive mark and alteration of H3K27 methylation has been found in several cancer types including leukemia [83]. Histone modifications are currently being explored as potential biomarkers for disease progression and prognosis [84]. The induction of H3K27me2/me3 and H3K36me2 in TF-1 cells suggests a potential for these epigenetic marks to serve as biomarkers that differentiate the early (MDS) and late stage (AML) of leukemia. However, if the alteration of H3K27 methylation contributes to myeloid leukemogenesis remains to be fully elucidated.

### Interplay between DNA methylation and histone modifications

Prior studies have pointed to the relatedness of DNA methylation and histone modifications in impacting tumor pathogenesis [8, 11, 28, 30–35]. Three major DNMTs (DNMT1, DNMT3A, and DNMT3B) are involved in DNA methylation in cells. All three DNMTs are post-translationally modified with a variety of PTMs including acetylation, methylation, phosphorylation, SUMOylation, and ubiquitination [85]. Reversible covalent modifications of DNMTs may affect their stability, enzyme activity, DNA binding, and interactions with other partners [85–87]. For example, SUMOylation of DNMT3A affects protein binding to HDACs and changes DNA methylation profiles [88, 89]. DNMT1 is the primary target trapped and depleted by DAC. Among its listed known interacting proteins are chromatin modifiers including HDAC1/2 (HDACs), KDM1A (HDM), SUV39H1 (HMT), EHMT2 (HMT), EZH2 (Polycomb group protein), EED (Polycomb group protein), SETD7 (HMT), KAT5 (HAT), and etc. [87].

The role of DNMTs in linking these two types of modifications is of particular interest. In this study, we have demonstrated that DAC-induced DNA hypomethylation has significant impact on chromatin modifications. We have revealed five particular histone marks (H3K27me1, H3K36me1, H3K27me1K36me1, H3.3K36me3, and H4K8acK12acK16ac) that were differentially expressed in MDS-L and TF-1 sensitive and resistant cells in response to DAC treatment, pointing to a potential role for these modifications in drug resistance mechanisms. These findings suggest a DNMT-dependent pathway through which DAC inhibits DNMTs and re-activates downstream tumor suppressor genes via histone modifying enzymes or other unknown factors.

## Conclusion

We have developed a fast proteomic method for robust quantitative analysis of histone PTMs. (1) The proteomic strategy described here enhances data quality and acquisition sensitivity resulting in comprehensive analysis of histone modifications in relatively small number of cells; (2) The systematic analysis of epigenetic profiles in drug sensitive and resistant cell lines have identified 61 PTMs, of which 15 are novel histone modifications; (3) The identification of H3.1K27me3 and H3.1/H3.3K27me2K36me2 as signature in TF-1 cells suggests their potential role in leukemogenesis; (4) The discovery of five additional histone marks that were differentially impacted by DAC in the sensitive and resistant cells suggests their potential relevance to the development of drug resistance. Our results also suggest a conserved, DNMT-dependent pathway in DAC-mediated leukemia treatment. More investigations, however, are needed to elucidate how DAC exerts its anti-tumor effect and how tumor cells develop a resistant strategy to escape the DAC-mediated anti-tumor therapy.

## Additional files

10.1186/s12014-016-9115-z 22 histone variants and their relative levels in the different groups.
10.1186/s12014-016-9115-z A list of 107 modified peptide species. Note: Trypsin cleaves K if propionylation is incomplete.
10.1186/s12014-016-9115-z Figure representation of all 61 individual PTMs in linker and core histones.
10.1186/s12014-016-9115-z Tandem spectra of 108 peptide species with PTMs.
10.1186/s12014-016-9115-z Relative levels of 60 PTM combinations in the different groups.
10.1186/s12014-016-9115-z A list of 107 modified peptide species. Note: Trypsin cleaves K if propionylation is incomplete.

## Abbreviations

ac: acetylation
me1: mono-methylation
me2: dimethylation
me3: tri-methylation
pr: propionylation
pm: propionylation and monomethylation
DNMT1: DNA (cytosine-5)-methyltransferase 1
DNMT3A: DNA (cytosine-5)-methyltransferase 3A
DNMT3B: DNA (cytosine-5)-methyltransferase 3 beta
HAT: histone acetyltransferase
HDAC: histone deacetylase
HMT: histone methyltransferase
HDM: histone demethylase
HATi: histone acetyltransferase inhibitor
HDACi: histone deacetylase inhibitor
HMTi: histone methyltransferase inhibitor
HDMi: histone demethylase inhibitor
MS1: a first stage of mass spectrometry
MS2: tandem mass spectrometry or MS/MS
PMSF: phenylmethylsulfonyl fluoride
SILAC: stable isotope labeling by amino acids in cell culture
