# Supplementary material for: Quantitative proteomic analysis of histone modifications in decitabine sensitive and resistant leukemia cell lines
Source: Clin Proteomics. 2016 Jul 5;13:14. doi: 10.1186/s12014-016-9115-z (PMC4932764; doi:10.1186/s12014-016-9115-z)
Supplement: Supplementary file 3 — 10.1186/s12014-016-9115-z Figure representation of all 61 individual PTMs in linker and core histones. [file 12014_2016_9115_MOESM3_ESM.pptx]

## Slide 1
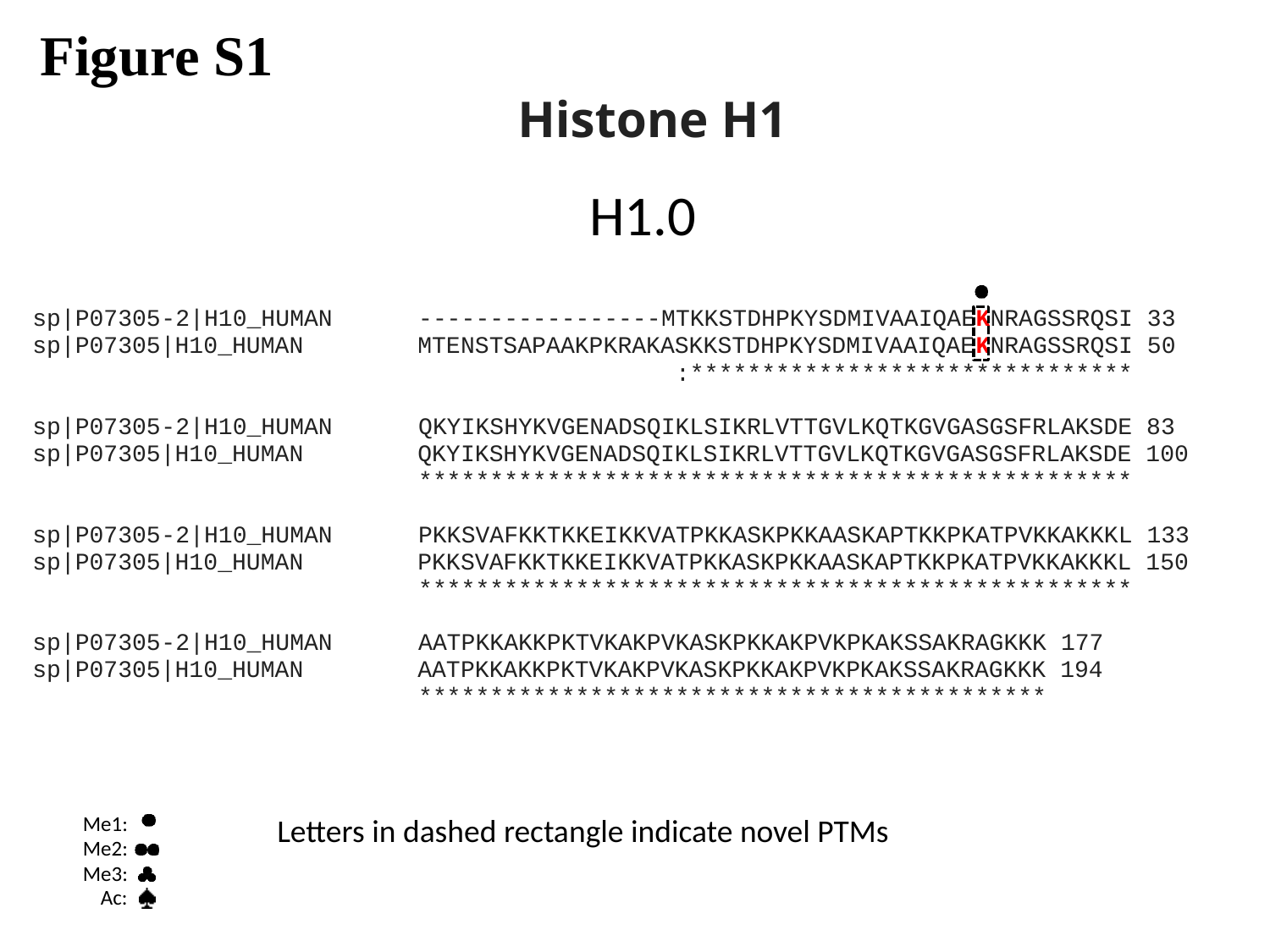

Figure S1
Histone H1
H1.0
Me1:
Letters in dashed rectangle indicate novel PTMs
Me2:
Me3:
Ac:

## Slide 2
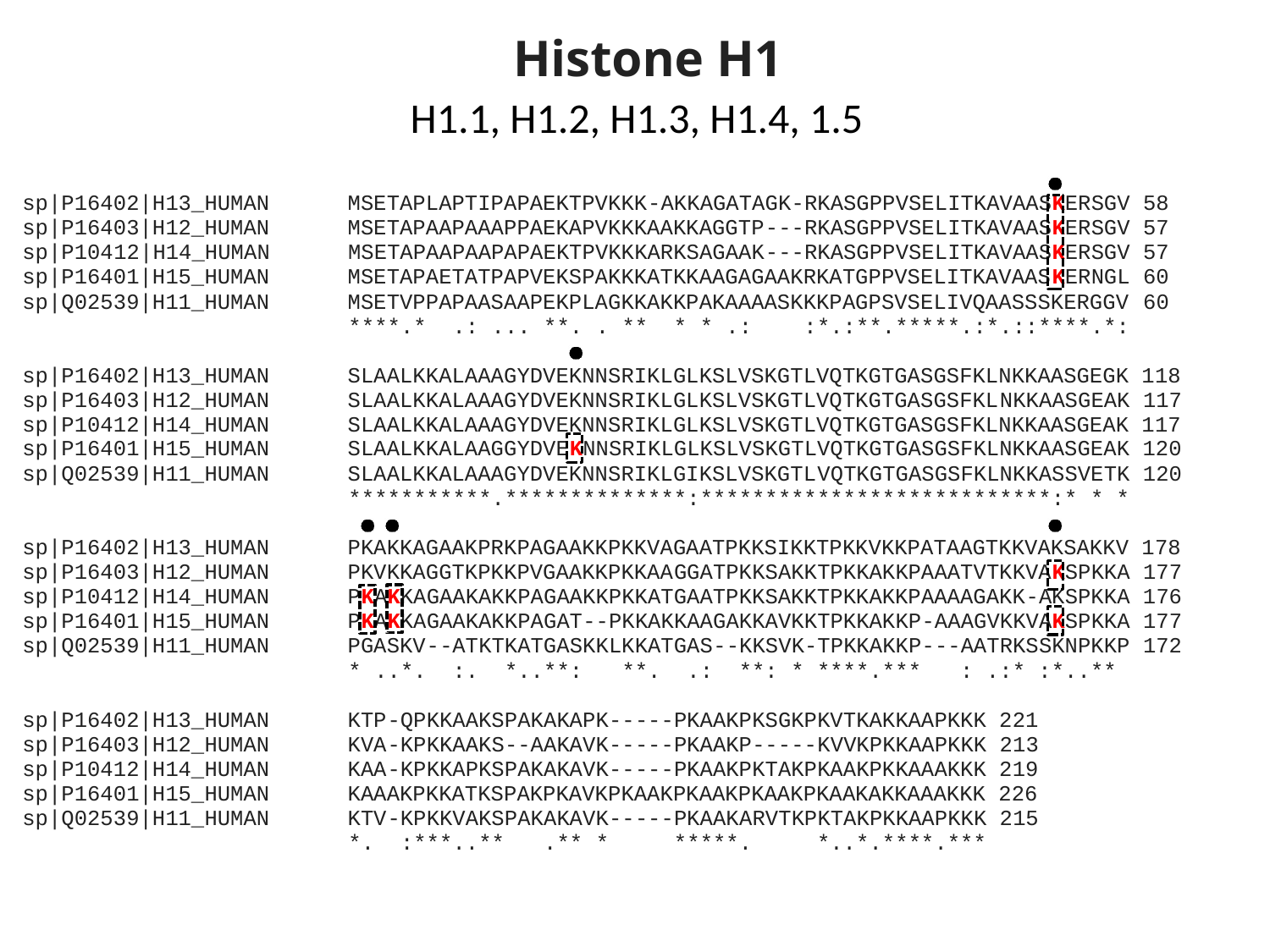

Histone H1
H1.1, H1.2, H1.3, H1.4, 1.5

## Slide 3
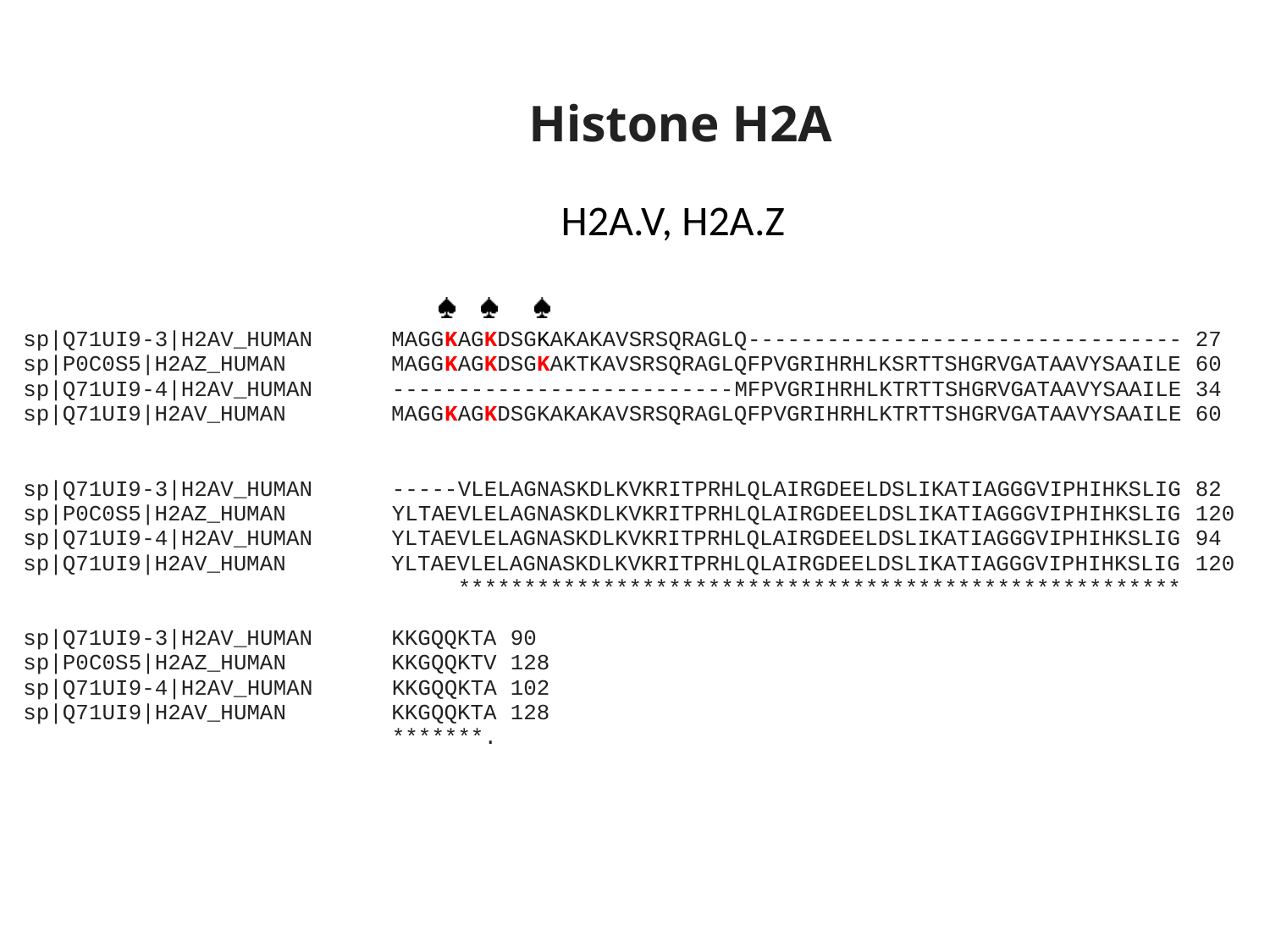

Histone H2A
H2A.V, H2A.Z

## Slide 4
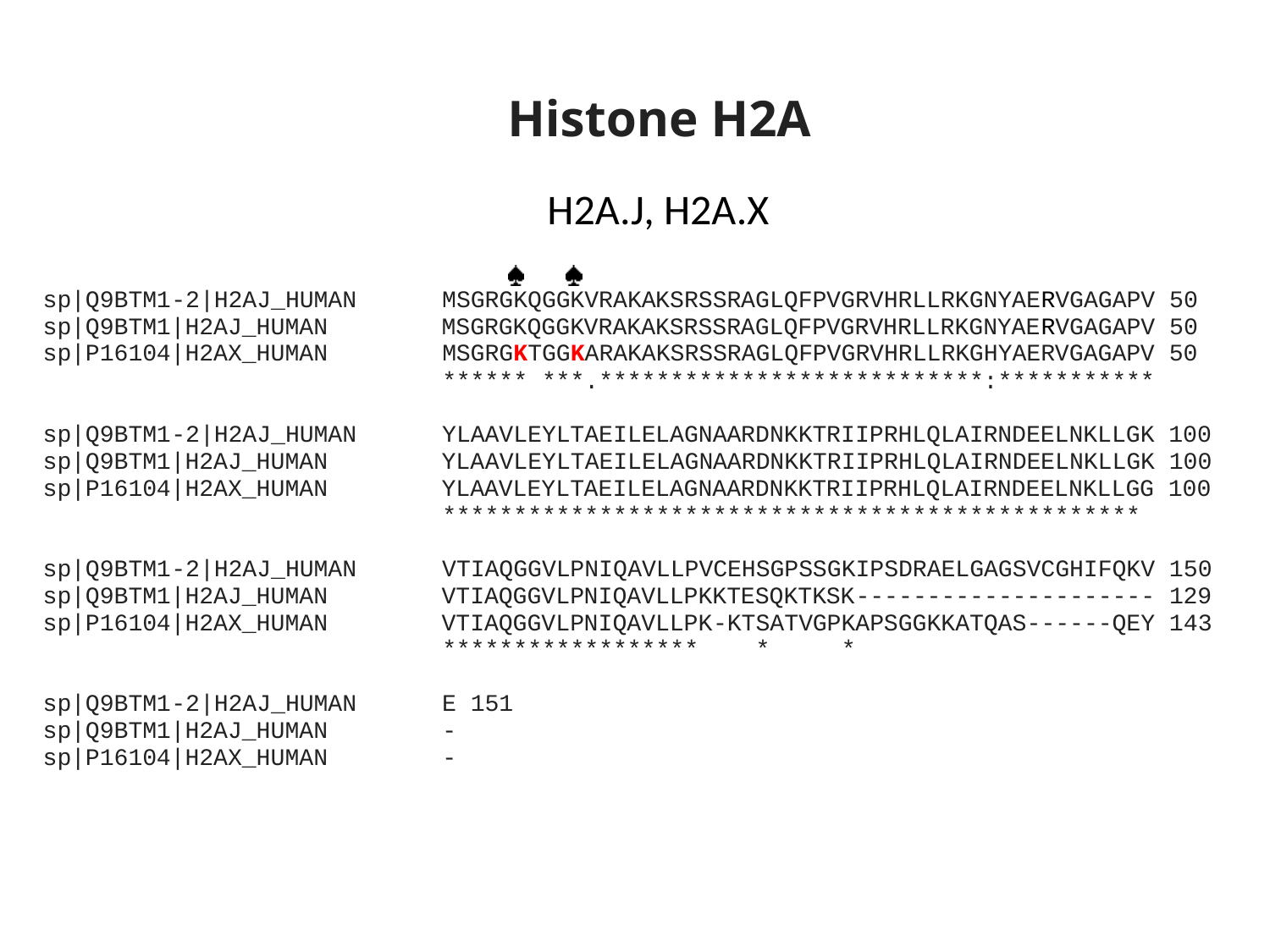

Histone H2A
H2A.J, H2A.X

## Slide 5
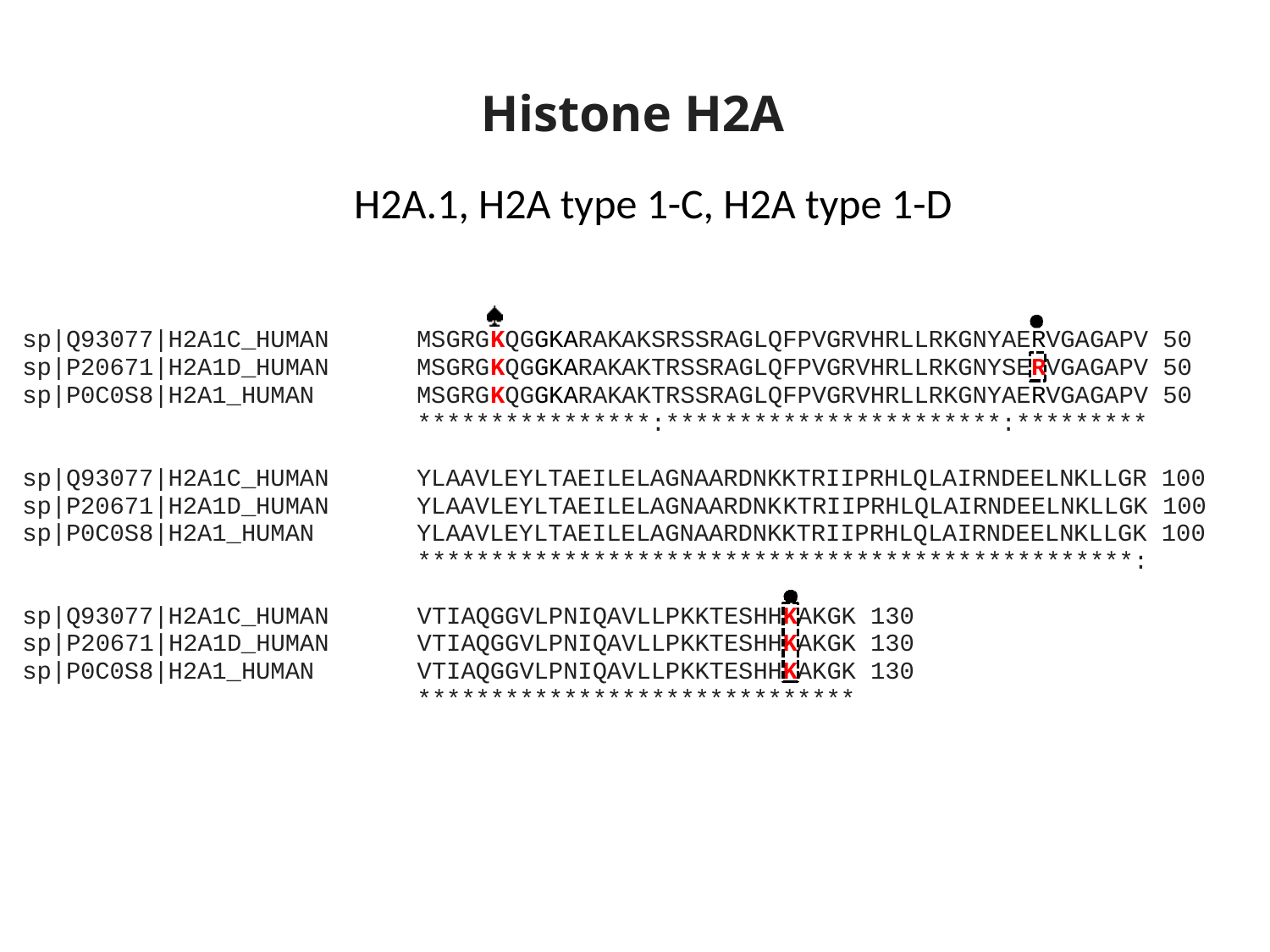

Histone H2A
H2A.1, H2A type 1-C, H2A type 1-D

## Slide 6
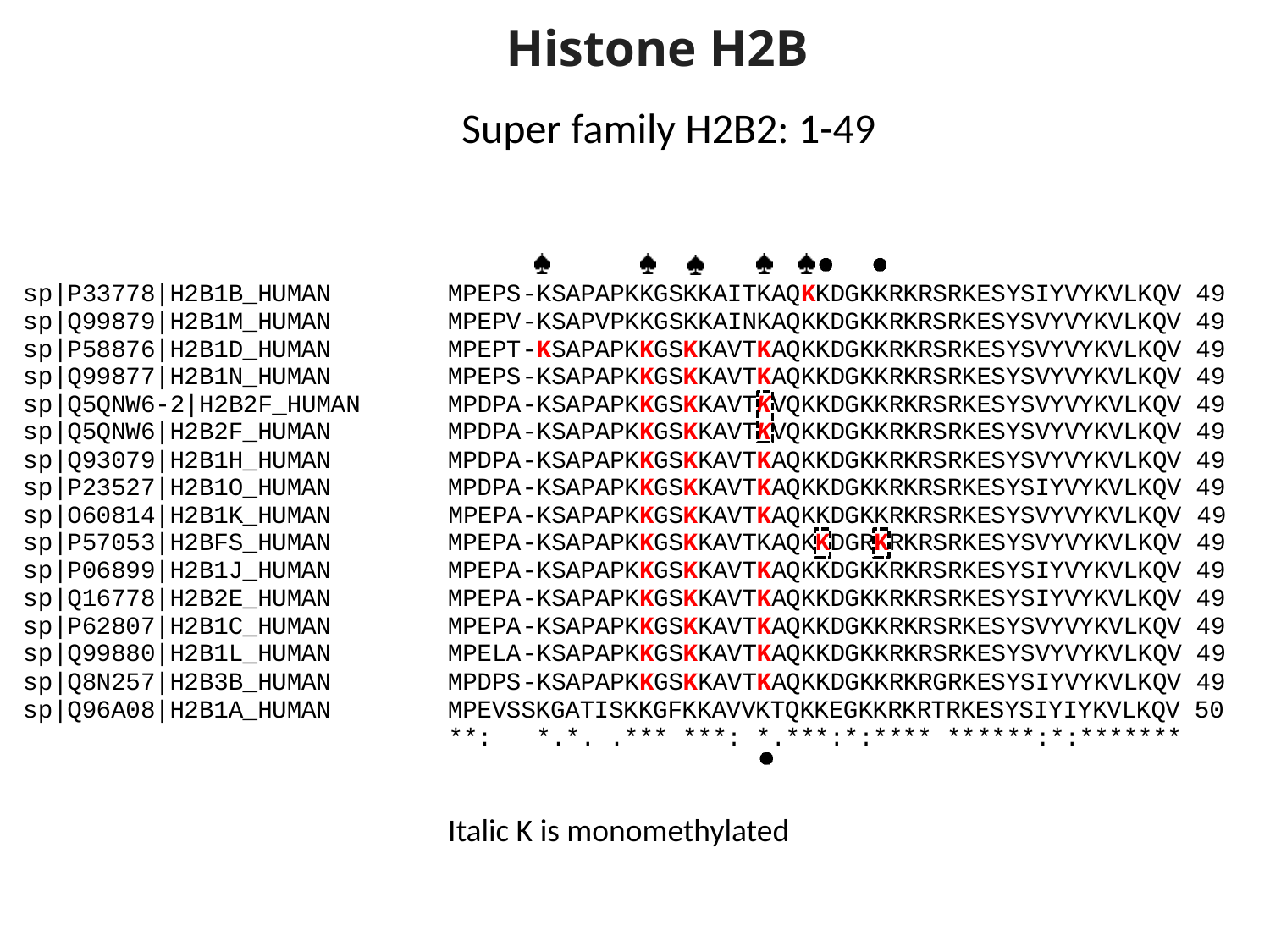

Histone H2B
Super family H2B2: 1-49
Italic K is monomethylated

## Slide 7
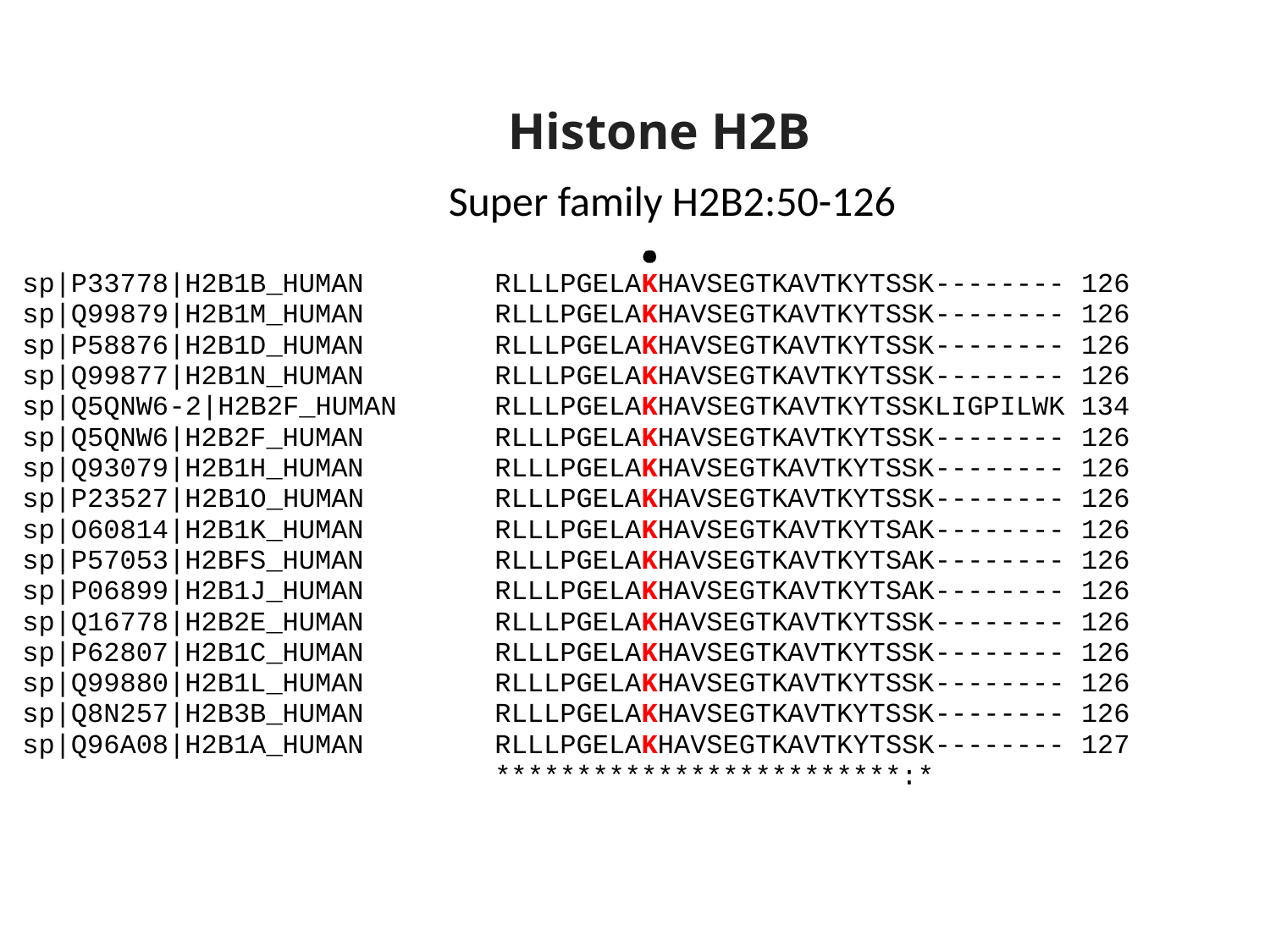

Histone H2B
Super family H2B2:50-126

## Slide 8
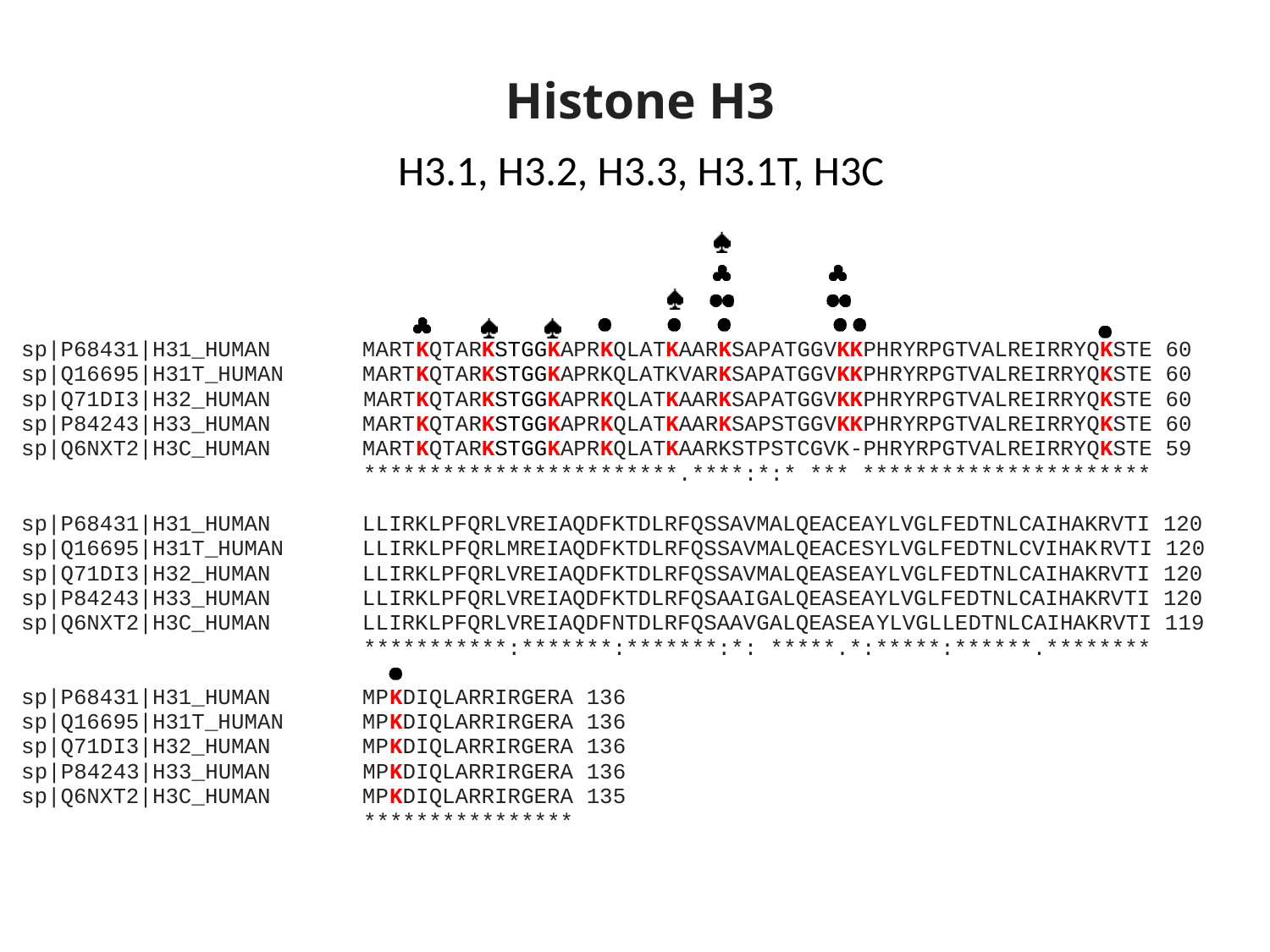

Histone H3
H3.1, H3.2, H3.3, H3.1T, H3C

## Slide 9
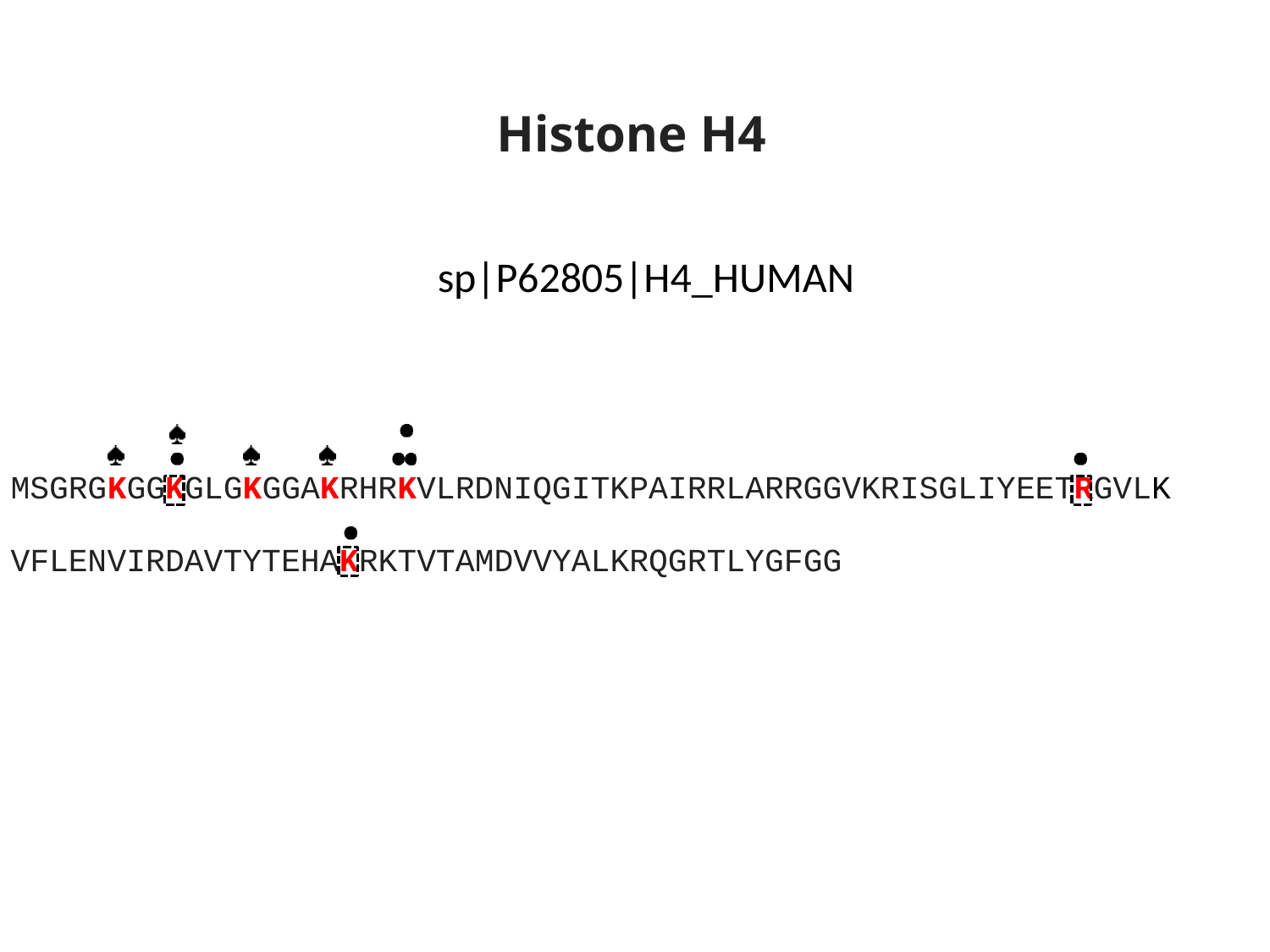

Histone H4
sp|P62805|H4_HUMAN
