# Supplementary material for: Quantitative proteomic analysis of histone modifications in decitabine sensitive and resistant leukemia cell lines
Source: Clin Proteomics. 2016 Jul 5;13:14. doi: 10.1186/s12014-016-9115-z (PMC4932764; doi:10.1186/s12014-016-9115-z)

Raw file	Scan	Method	Score	m/z
MR+3	7032	FTMS; HCD	71.18	558.29

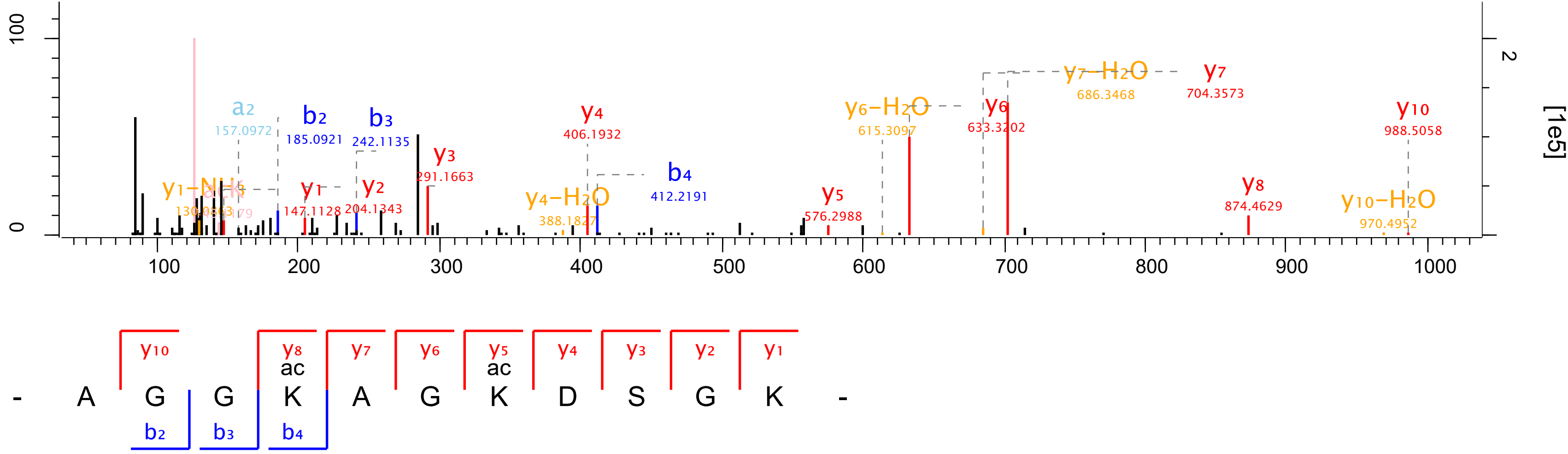

Raw file	Scan	Method	Score	m/z
MP-2	8512	FTMS; HCD	54.02	574.32

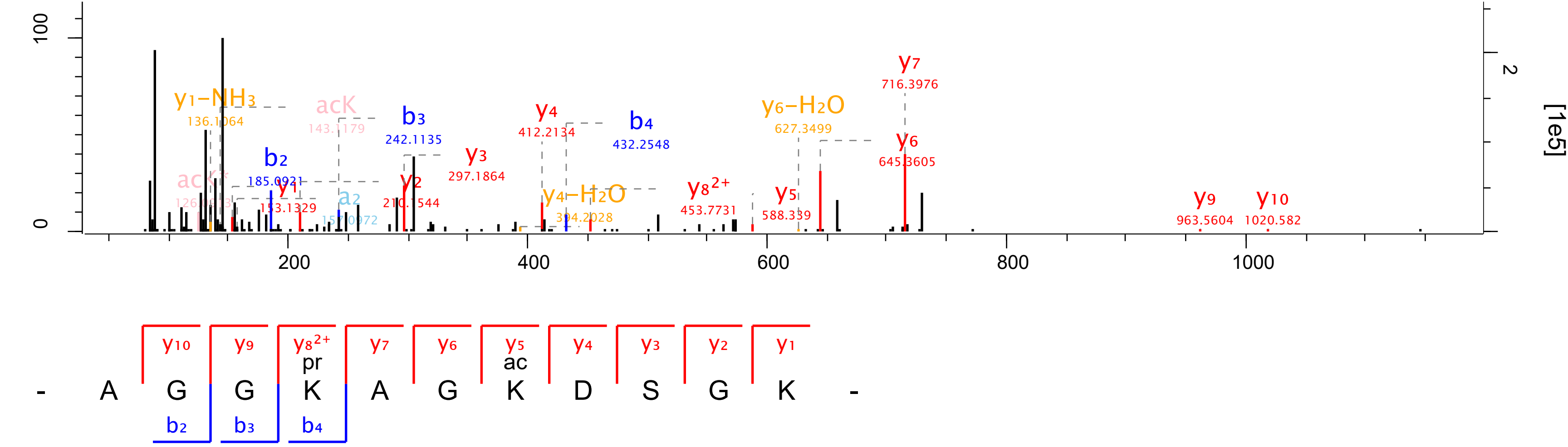

Raw file	Scan	Method	Score	m/z
TP+1	11893	FTMS; HCD	41.62	692.87

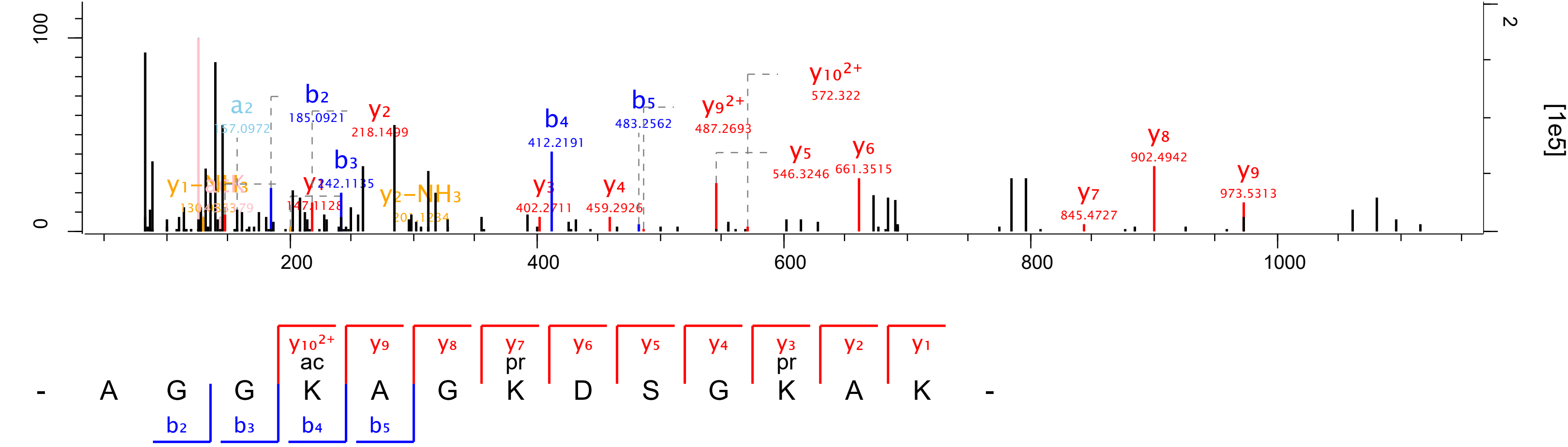

Raw file	Scan	Method	Score	m/z
MP-3	19663	FTMS; HCD	49.77	1048.58

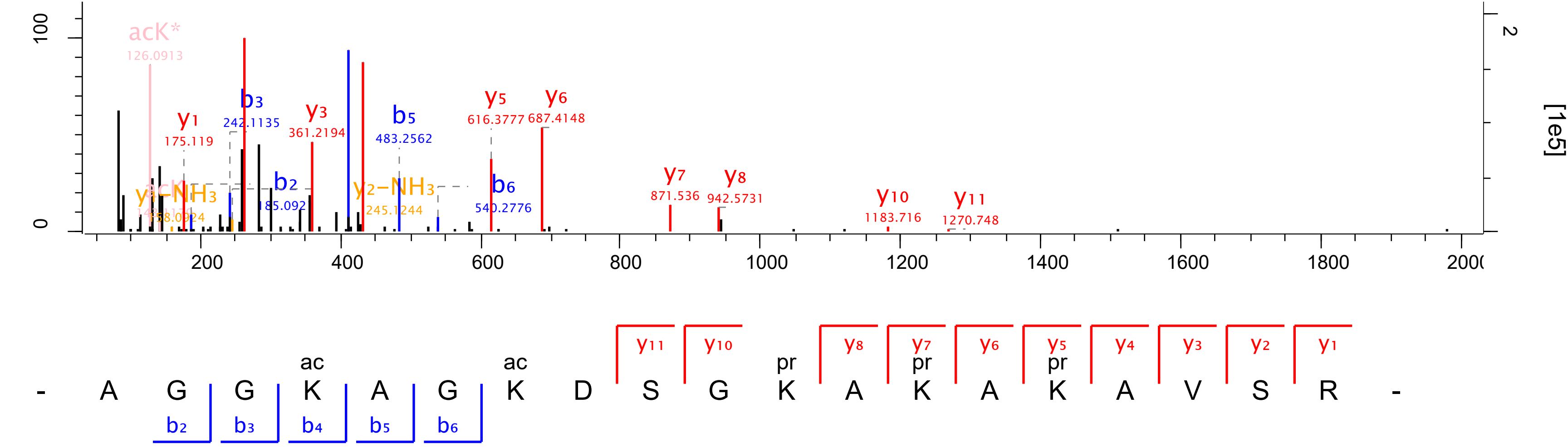

Raw file	Scan	Method	Score	m/z
TR+3	20679	FTMS; HCD	51.52	1056.09

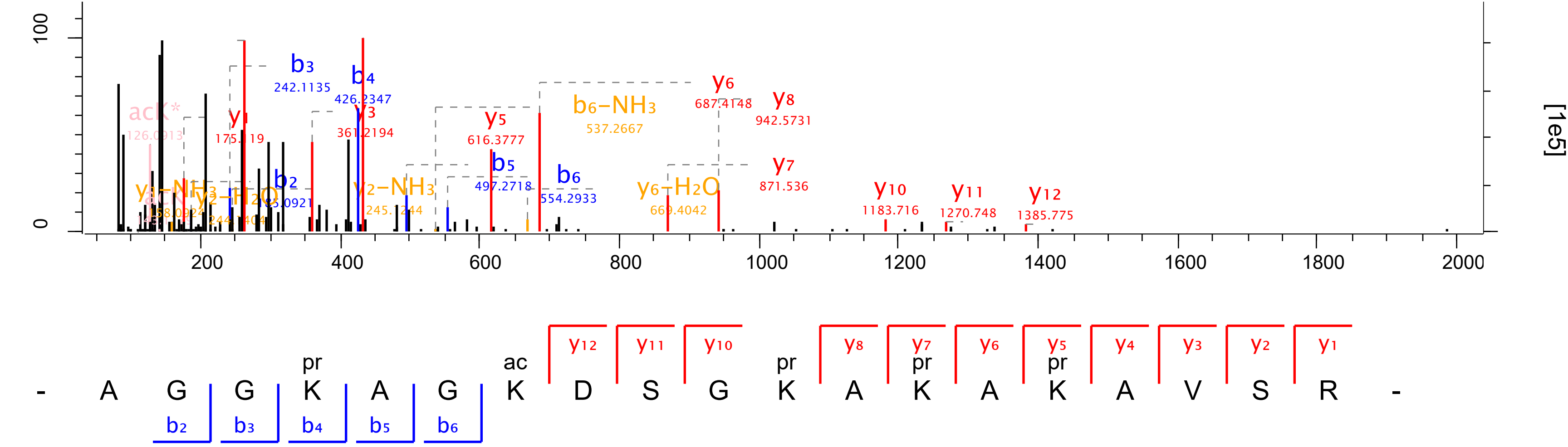

Raw file	Scan	Method	Score	m/z
TR-3	19113	FTMS; HCD	49.19	1063.59

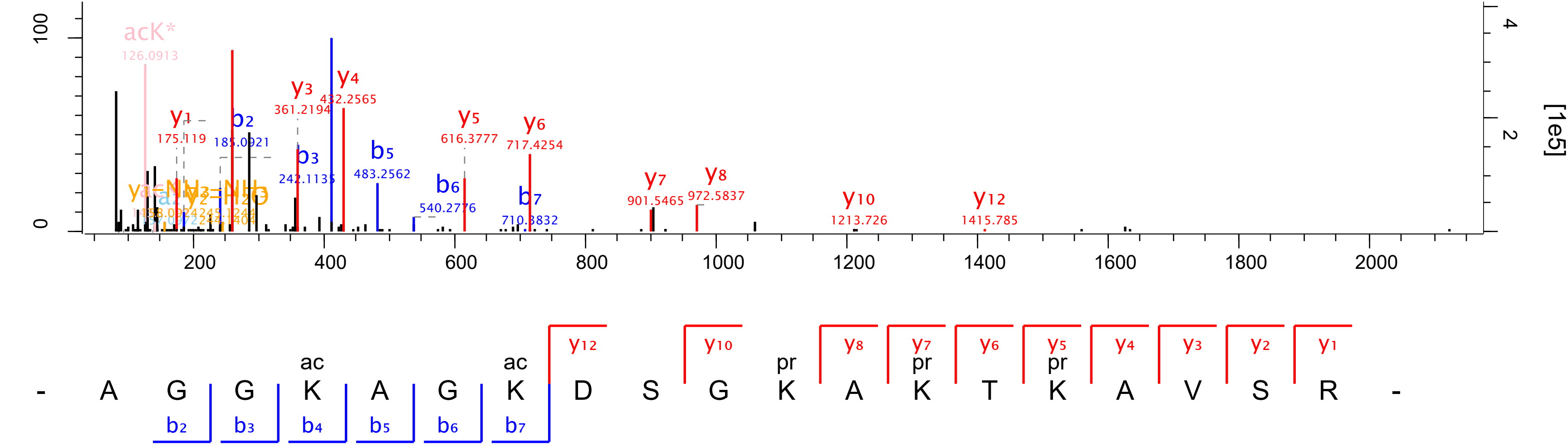

Raw file	Scan	Method	Score	m/z
MP+3	17308	FTMS; HCD	49.19	1056.07

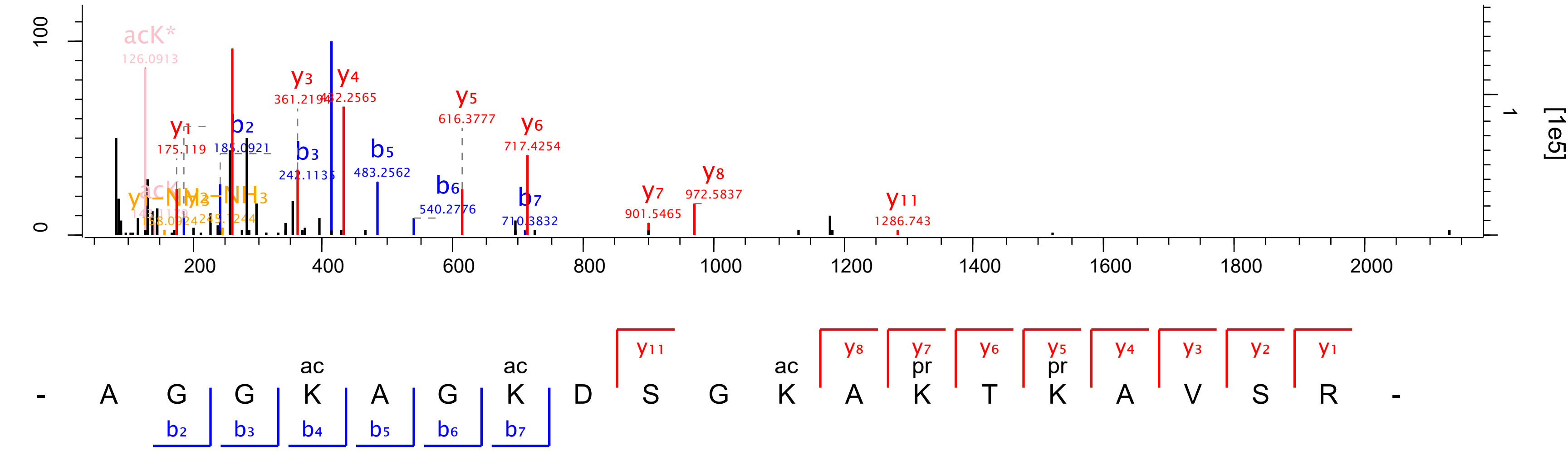

Raw file	Scan	Method	Score	m/z
TP+1	15847	FTMS; HCD	56.72	1042.58

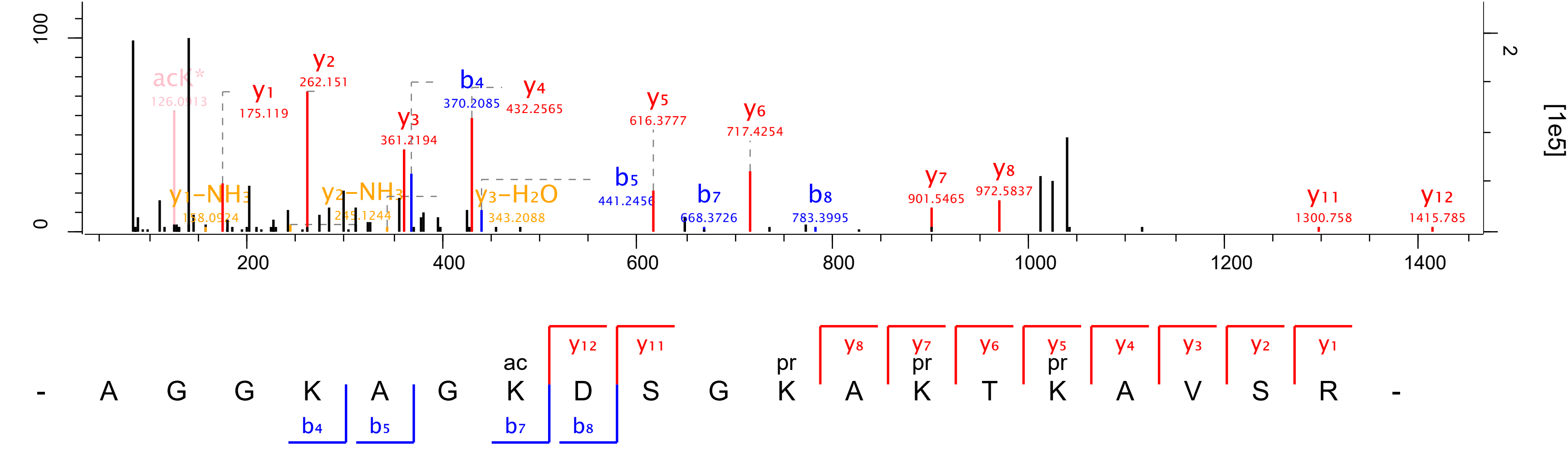

Raw file	Scan	Method	Score	m/z
MP-3	19057	FTMS; HCD	52.18	1070.59

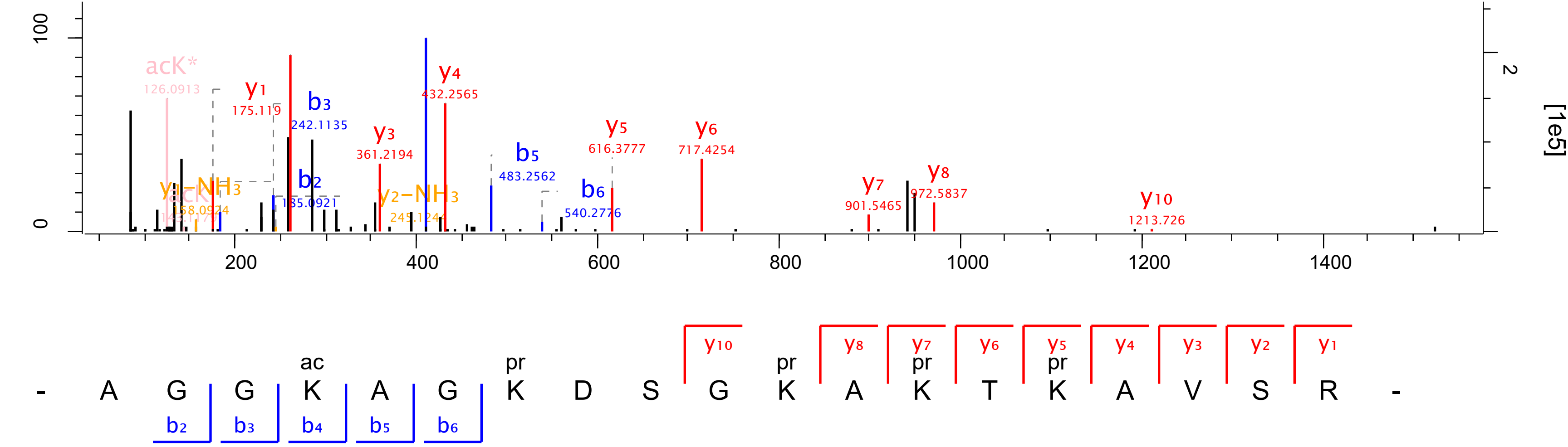

Raw file	Scan	Method	Score	m/z
MP-2	15550	FTMS; HCD	51.13	580.01

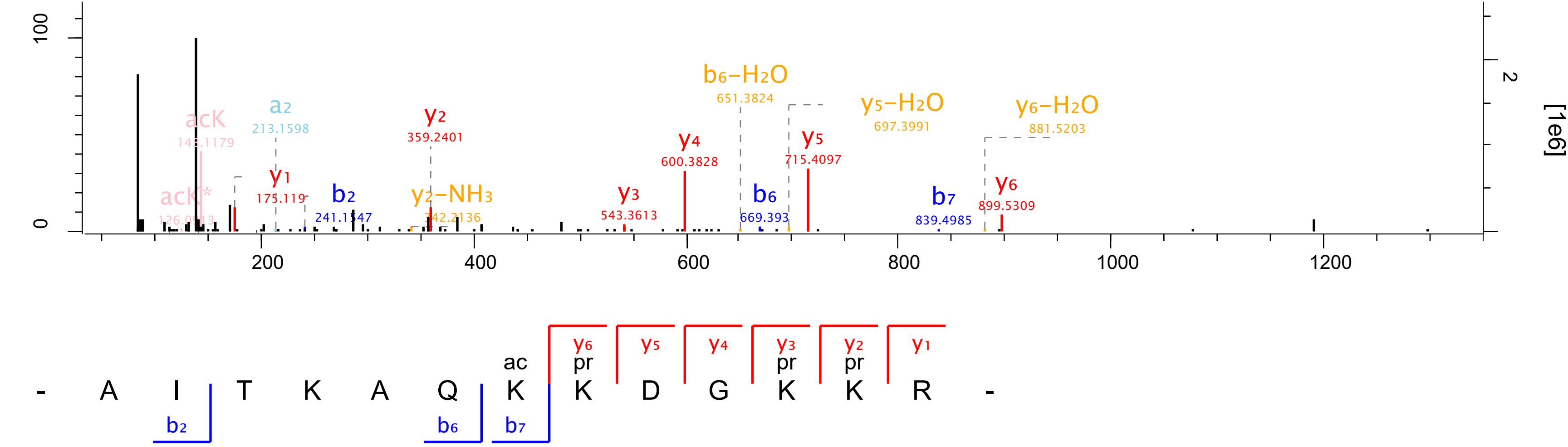

Raw file	Scan	Method	Score	m/z
MP-1	5948	FTMS; HCD	61.44	1012.59

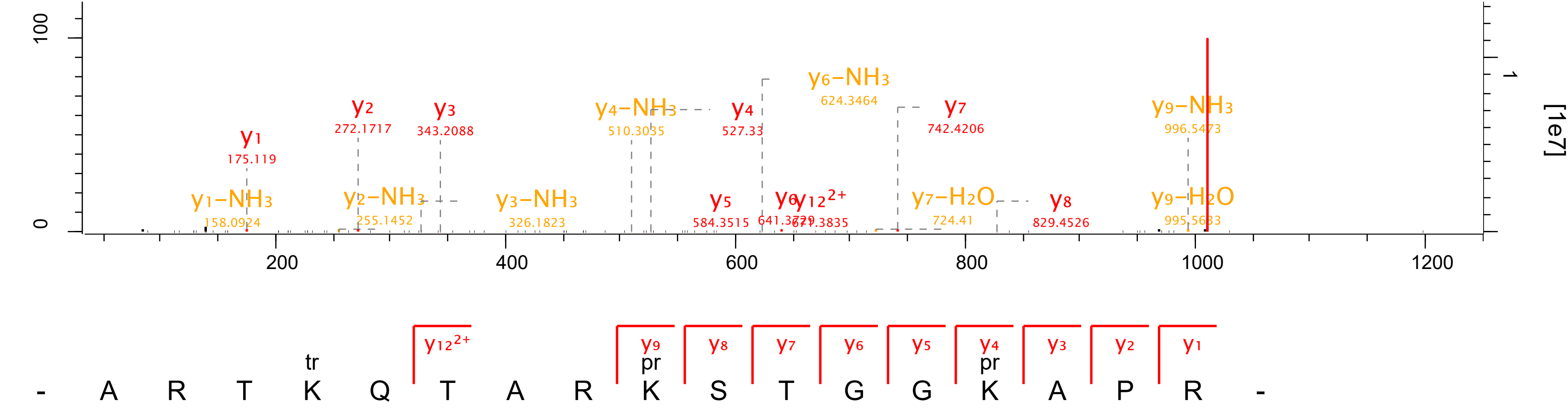

Raw file	Scan	Method	Score	m/z
TP+2	16002	FTMS; HCD	87.18	479.27

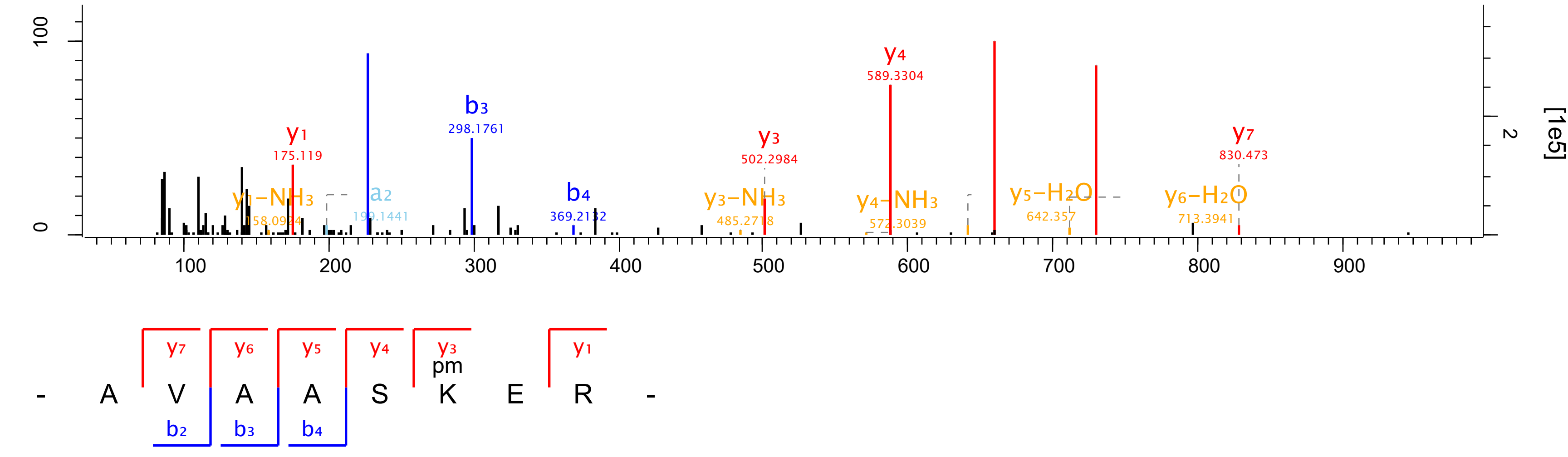

Raw file	Scan	Method	Score	m/z
MP+3	19851	FTMS; HCD	54.9	594.35

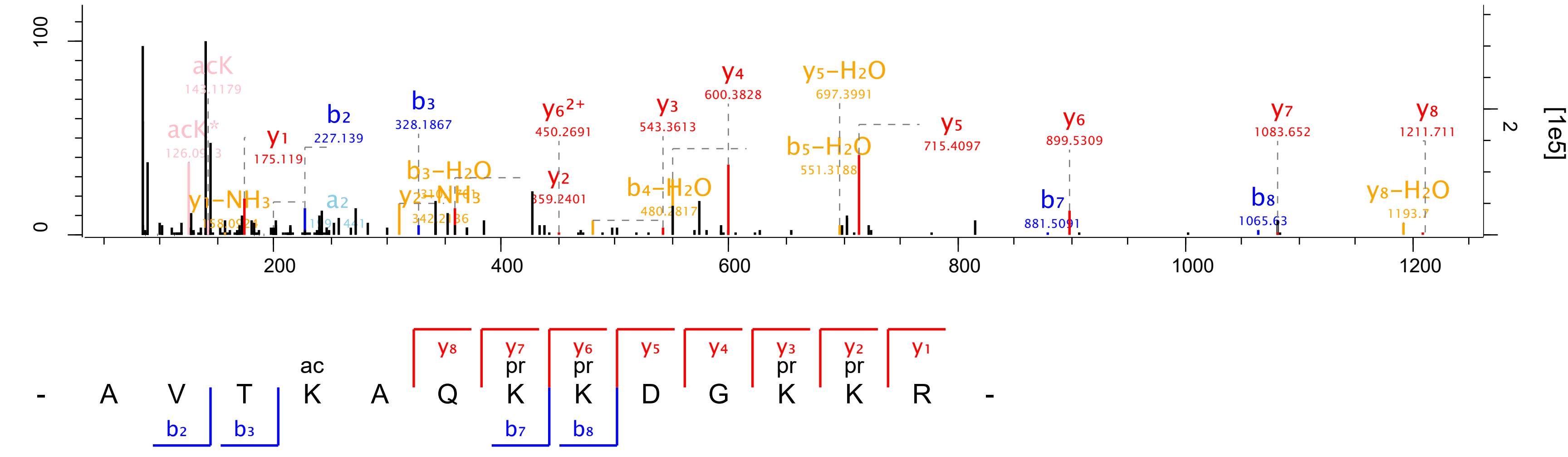

Raw file	Scan	Method	Score	m/z
TR-3	22453	FTMS; HCD	50.9	898.03

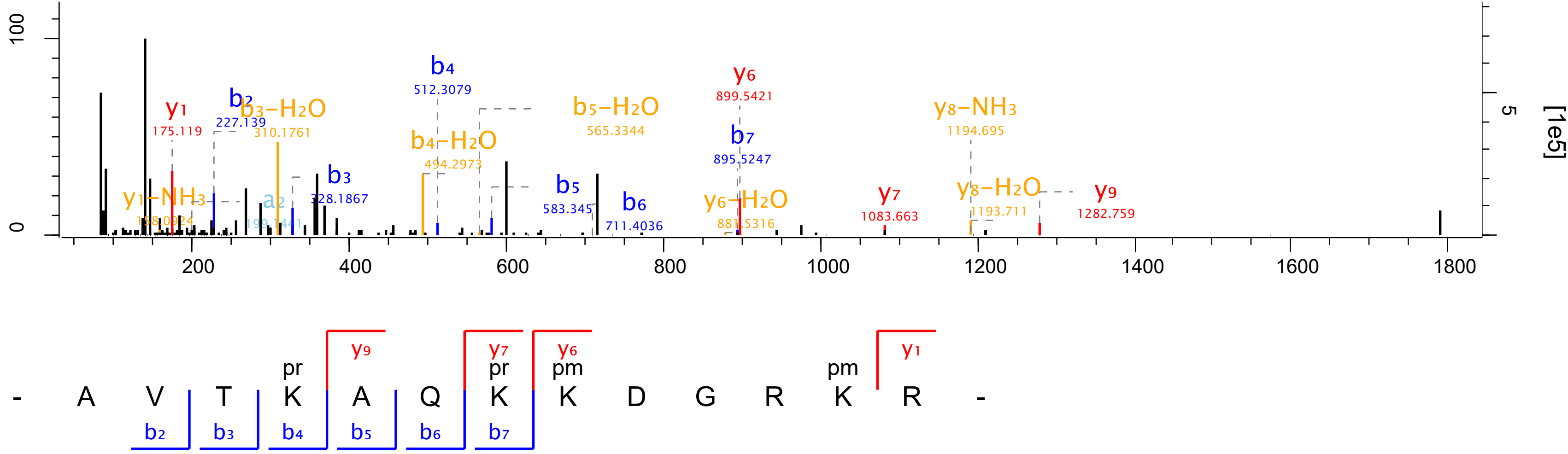

Raw file	Scan	Method	Score	m/z
TR+1	25139	FTMS; HCD	45.11	919.05

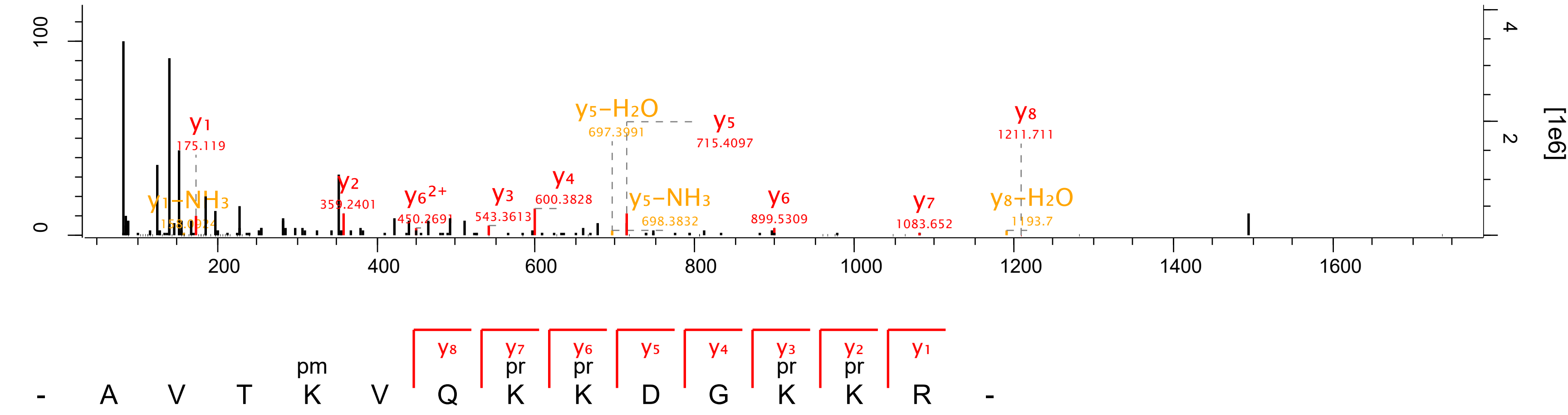

Raw file	Scan	Method	Score	m/z
MR+1	18351	FTMS; HCD	44.85	708.86

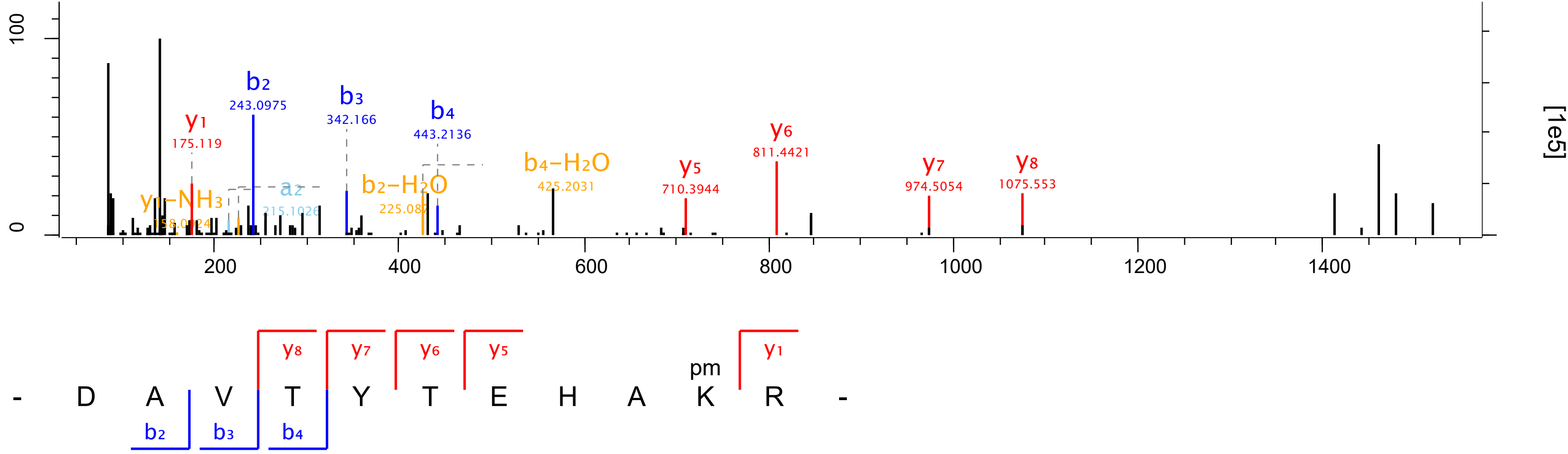

Raw file	Scan	Method	Score	m/z
TR-2	8843	FTMS; HCD	43.07	742.45

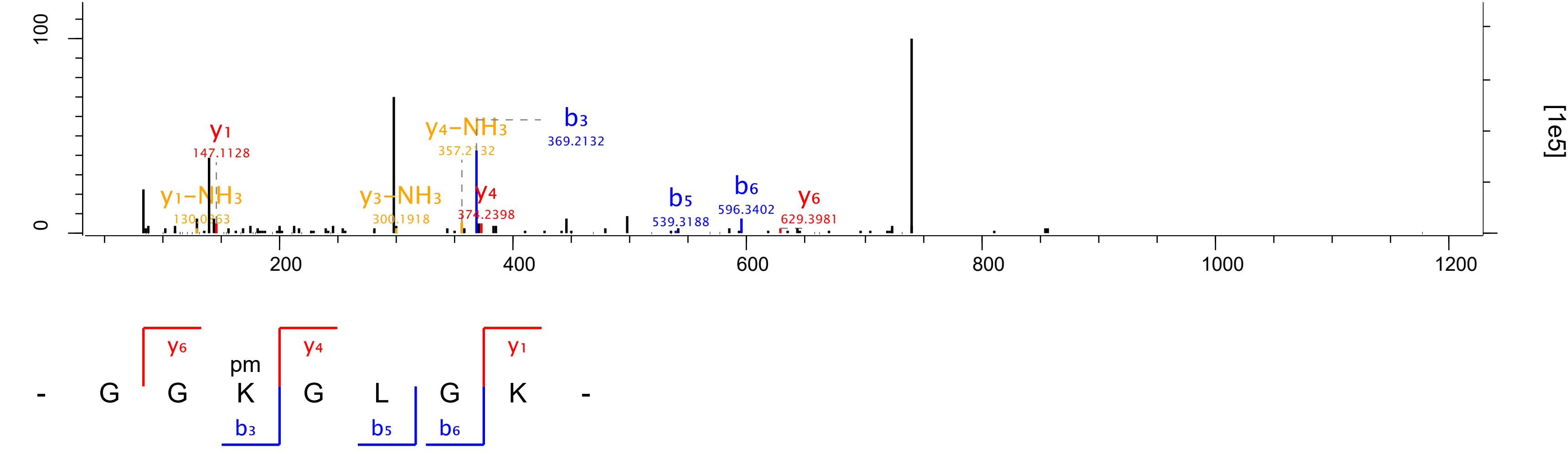

Raw file	Scan	Method	Score	m/z
TP+3	10388	FTMS; HCD	73.27	613.35

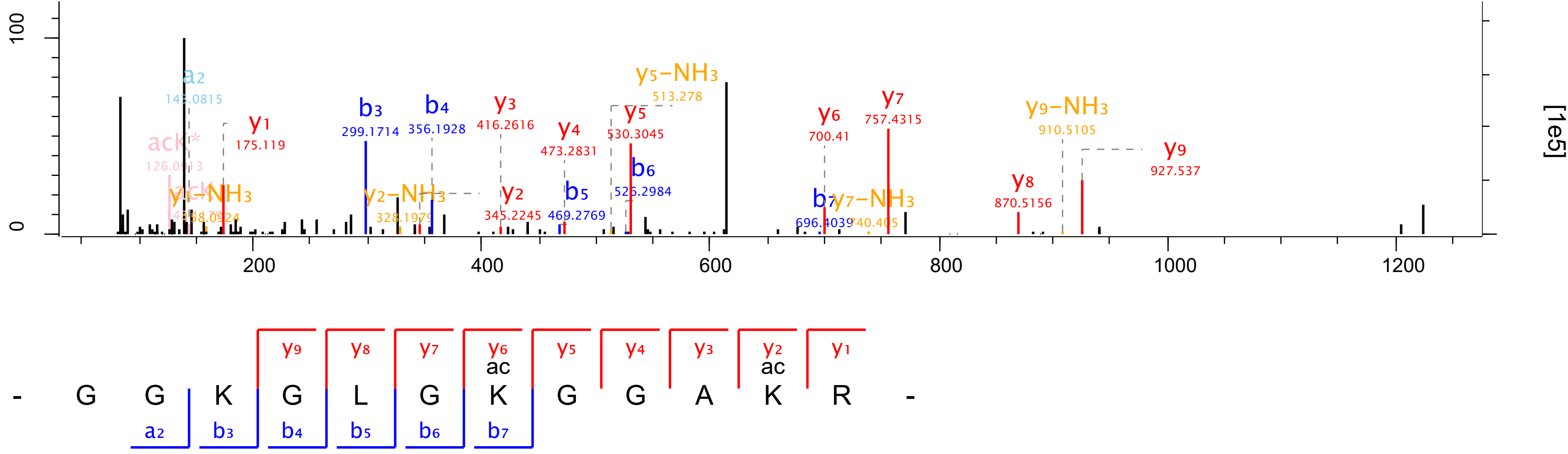

Raw file	Scan	Method	Score	m/z
TP-2	14814	FTMS; HCD	54.2	641.87

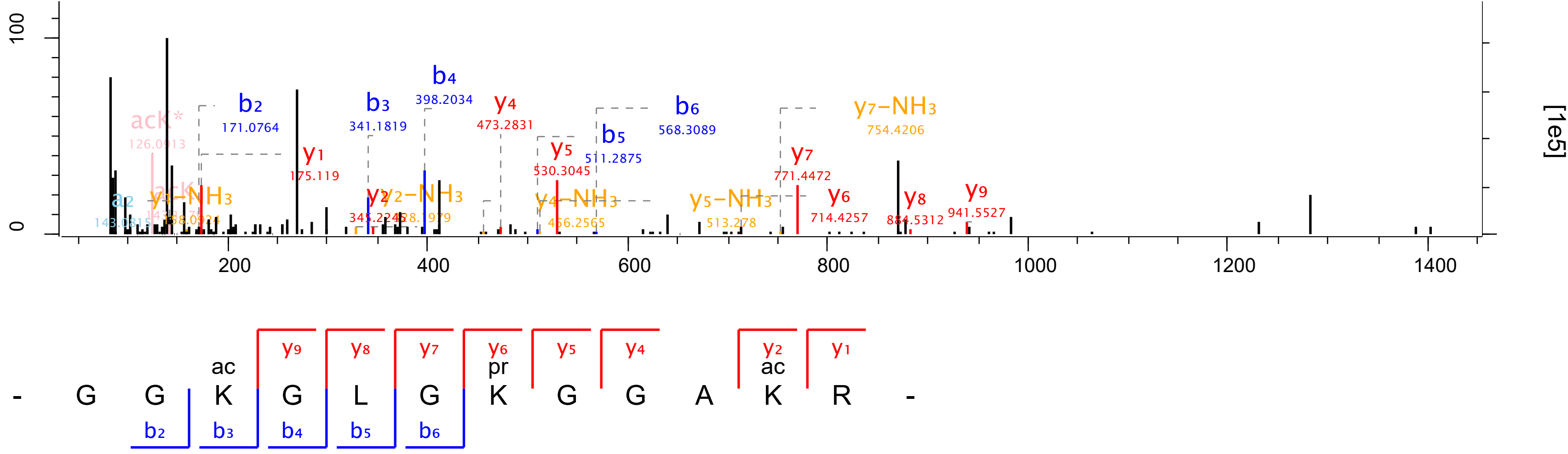

Raw file	Scan	Method	Score	m/z
TP-1	13336	FTMS; HCD	90.7	634.36

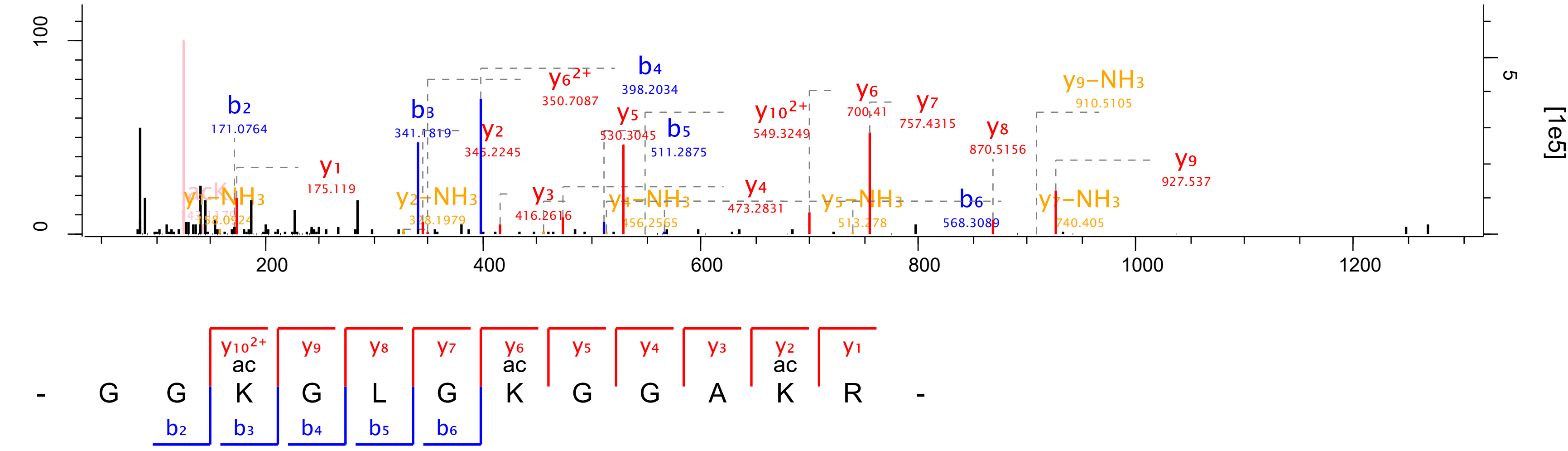

Raw file	Scan	Method	Score	m/z
TP-1	16496	FTMS; HCD	61.42	648.37

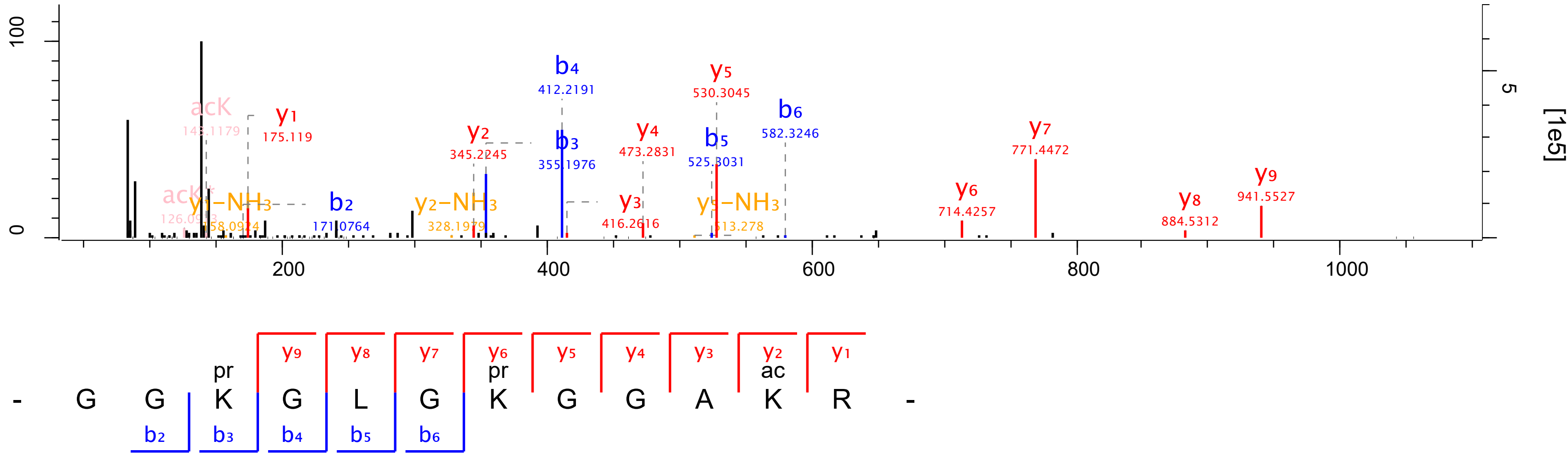

Raw file	Scan	Method	Score	m/z
MR+3	11856	FTMS; HCD	62.34	620.86

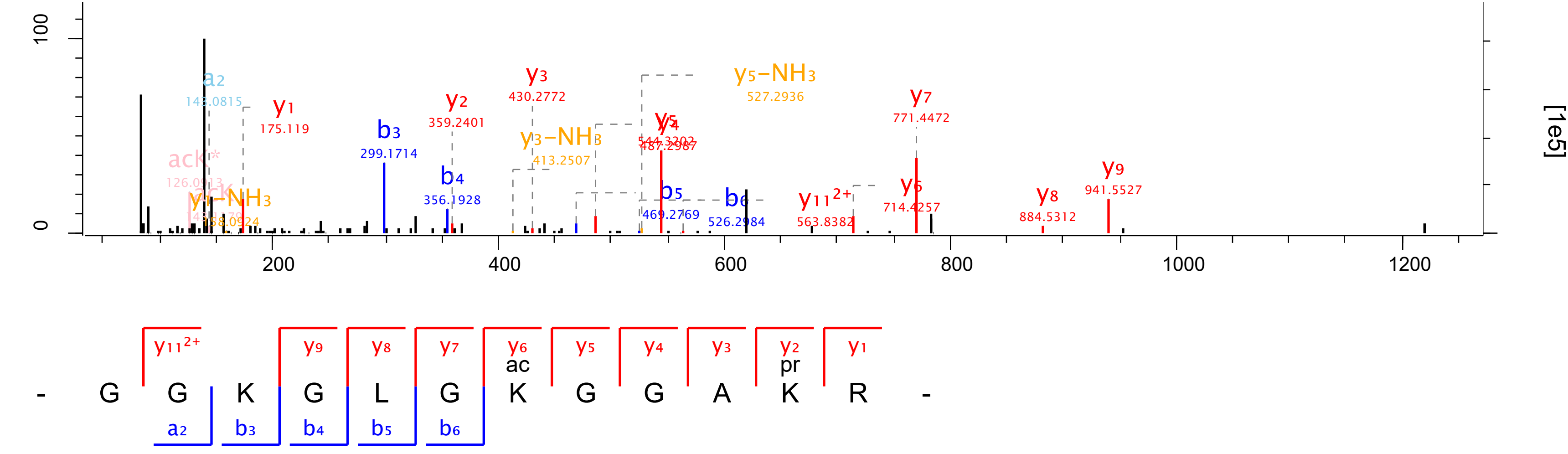

Raw file	Scan	Method	Score	m/z
MP-3	11015	FTMS; HCD	60.1	471.27

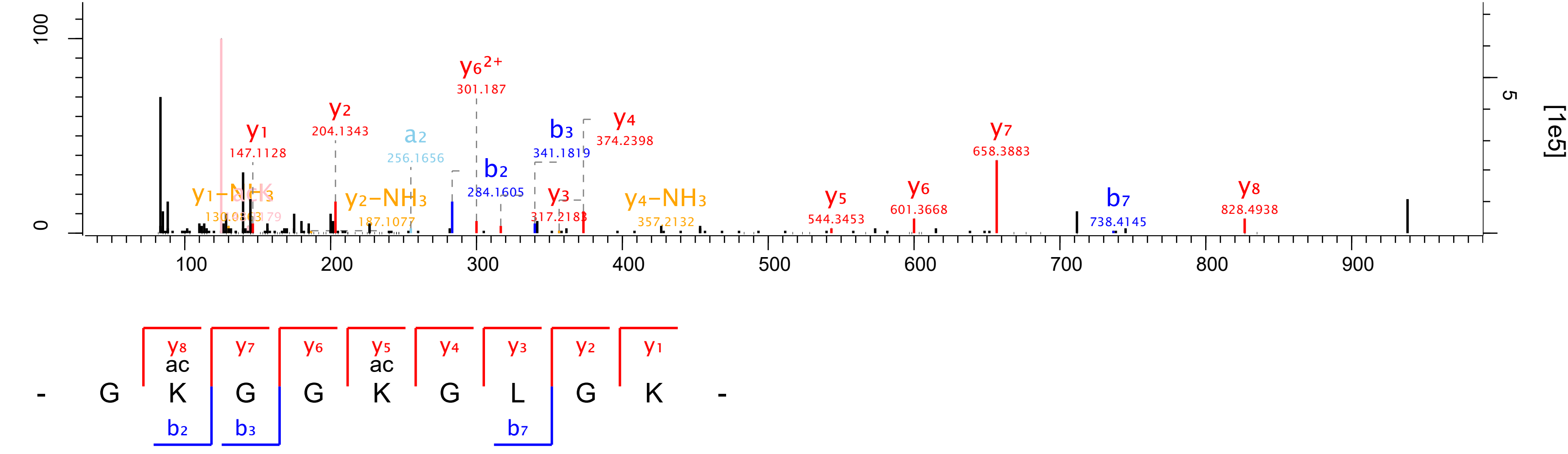

Raw file	Scan	Method	Score	m/z
TP+2	7754	FTMS; HCD	64.26	899.53

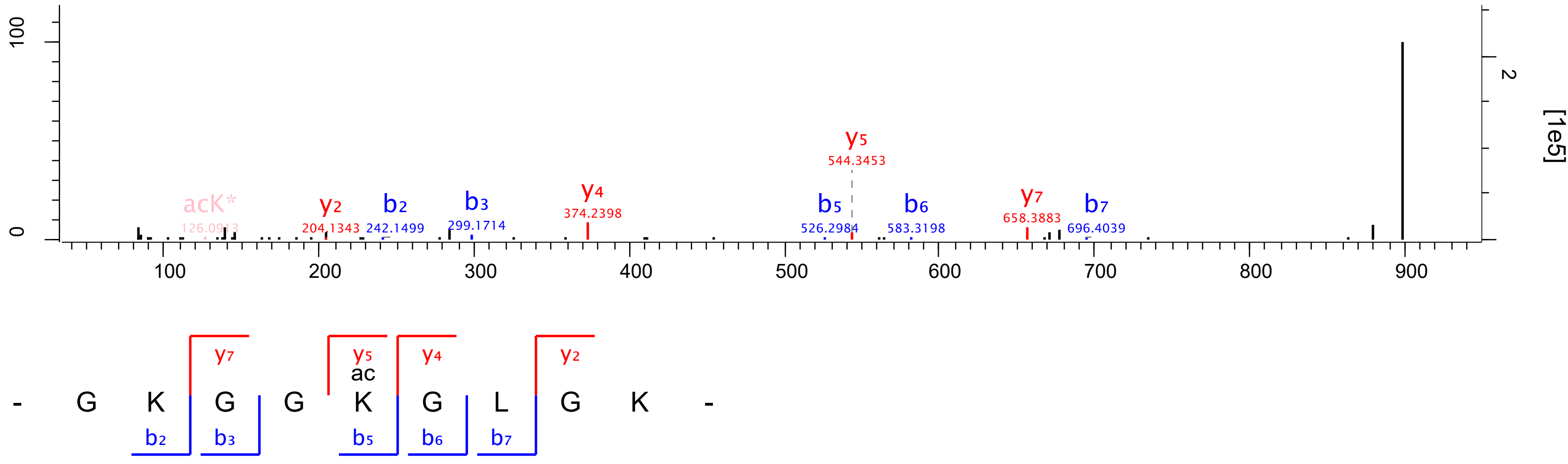

Raw file	Scan	Method	Score	m/z
MR-3	14082	FTMS; HCD	54.28	655.87

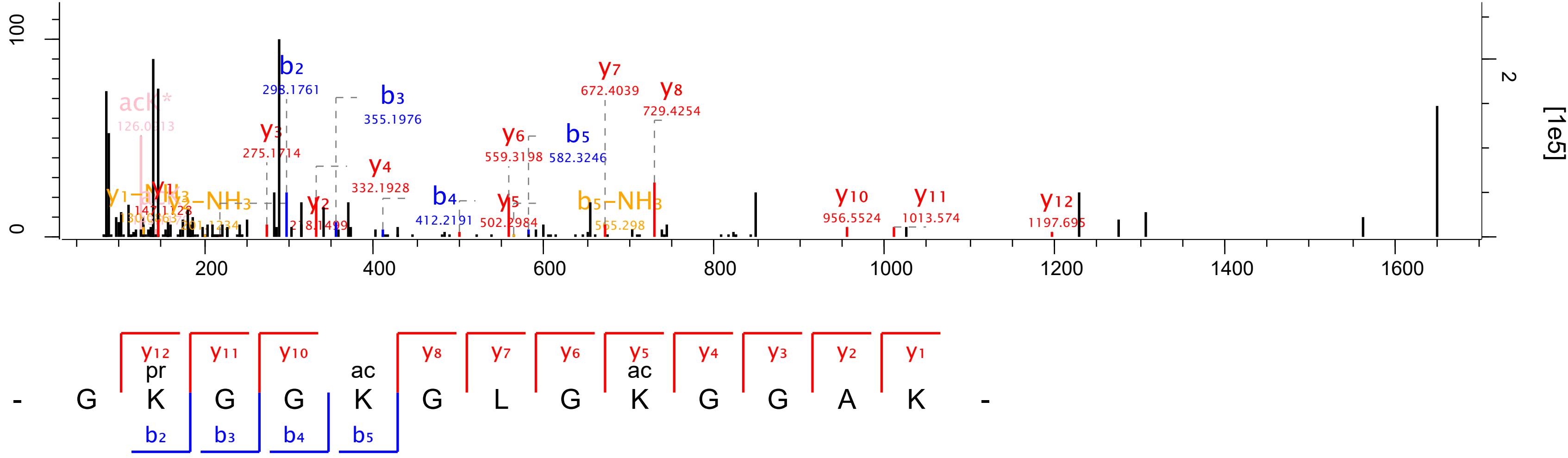

Raw file	Scan	Method	Score	m/z
TR-2	13079	FTMS; HCD	70.89	648.87

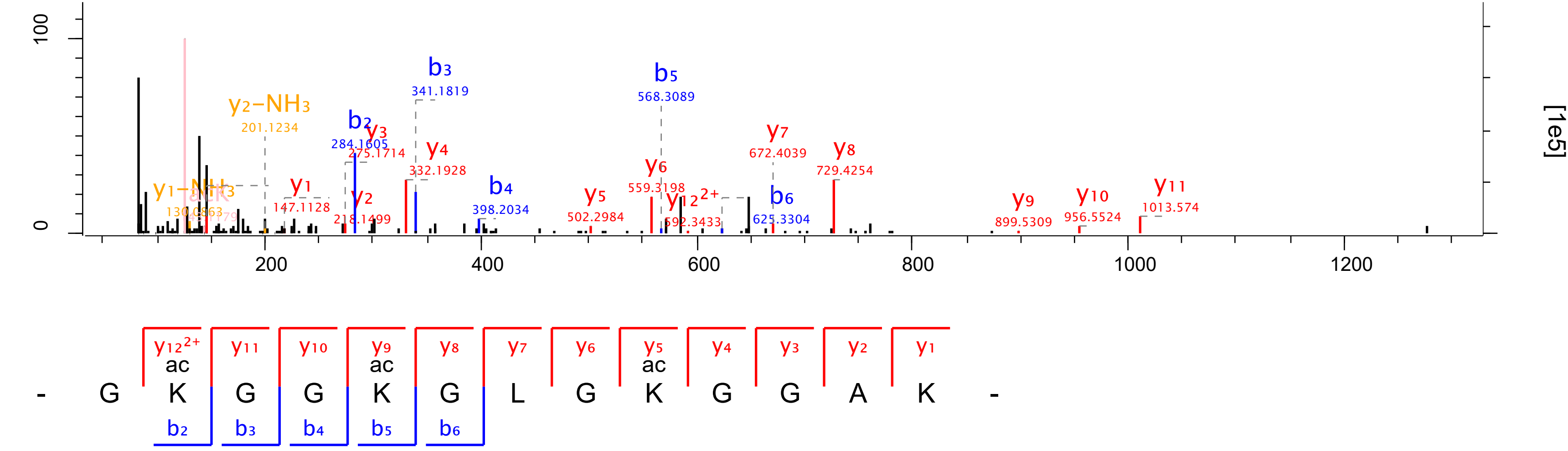

Raw file	Scan	Method	Score	m/z
MR-2	15417	FTMS; HCD	97.46	662.88

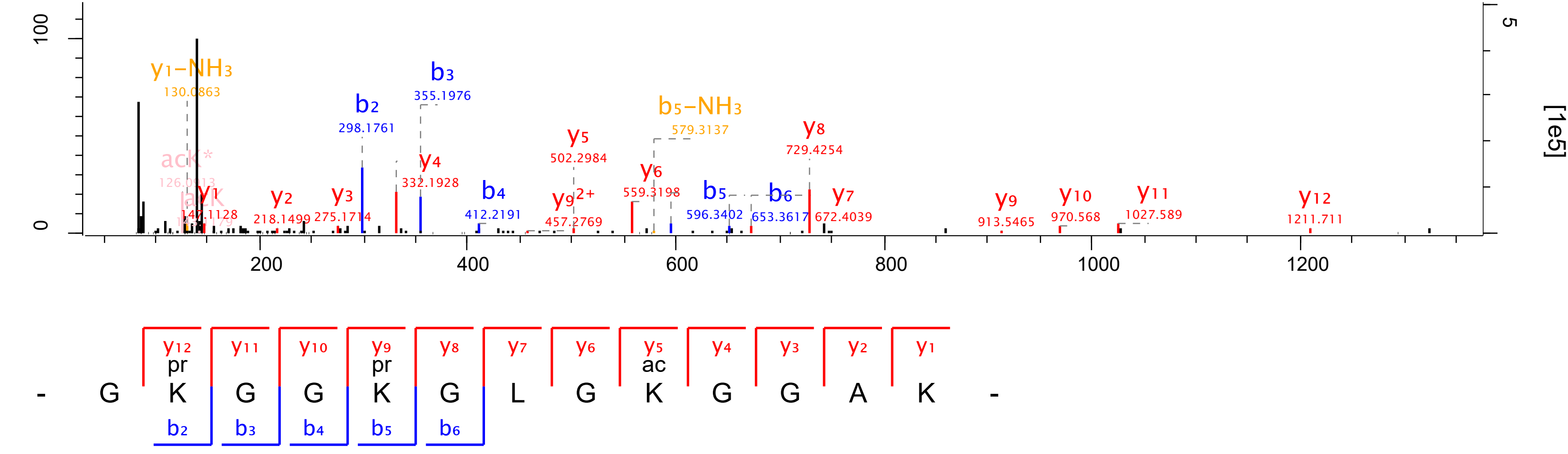

Raw file	Scan	Method	Score	m/z
TP-1	11470	FTMS; HCD	75.89	634.87

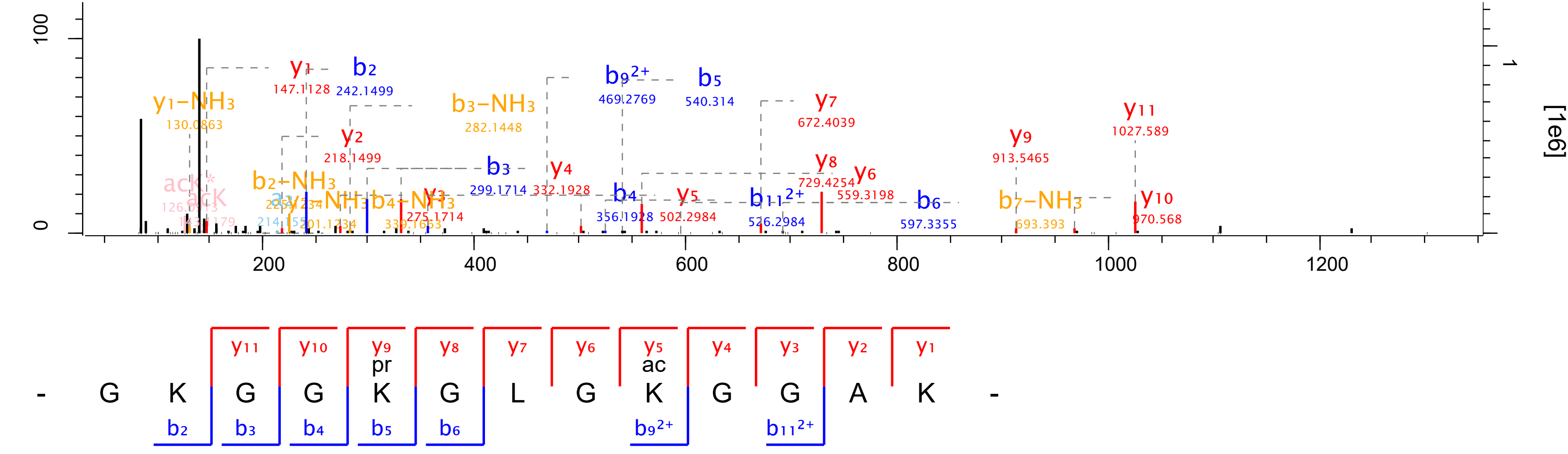

Raw file	Scan	Method	Score	m/z
TR-2	8415	FTMS; HCD	60.2	470.94

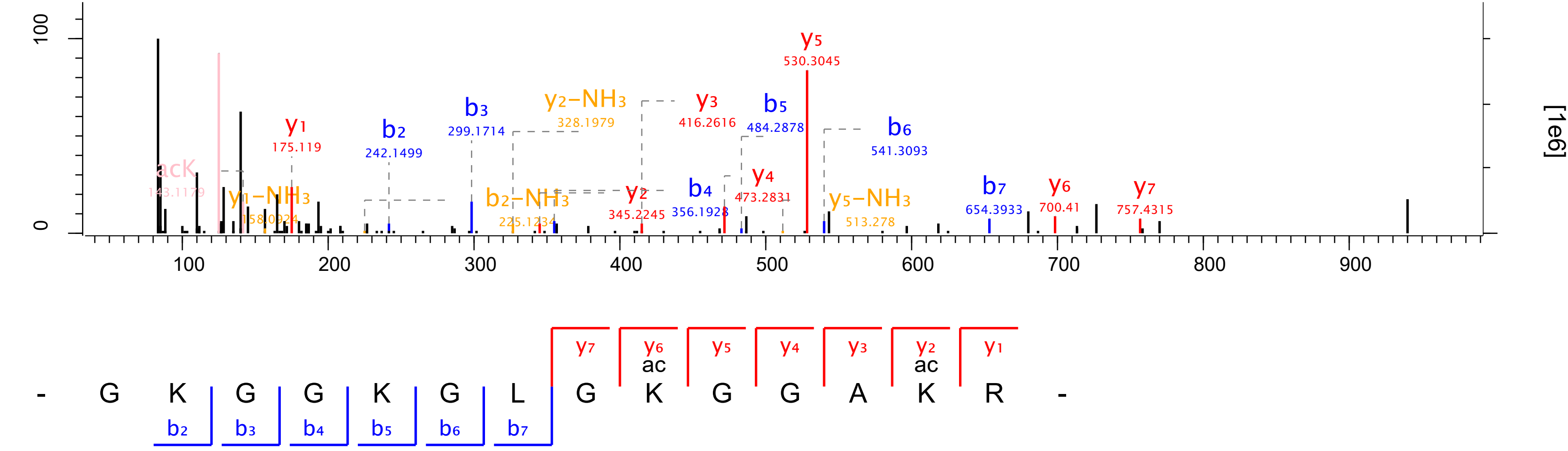

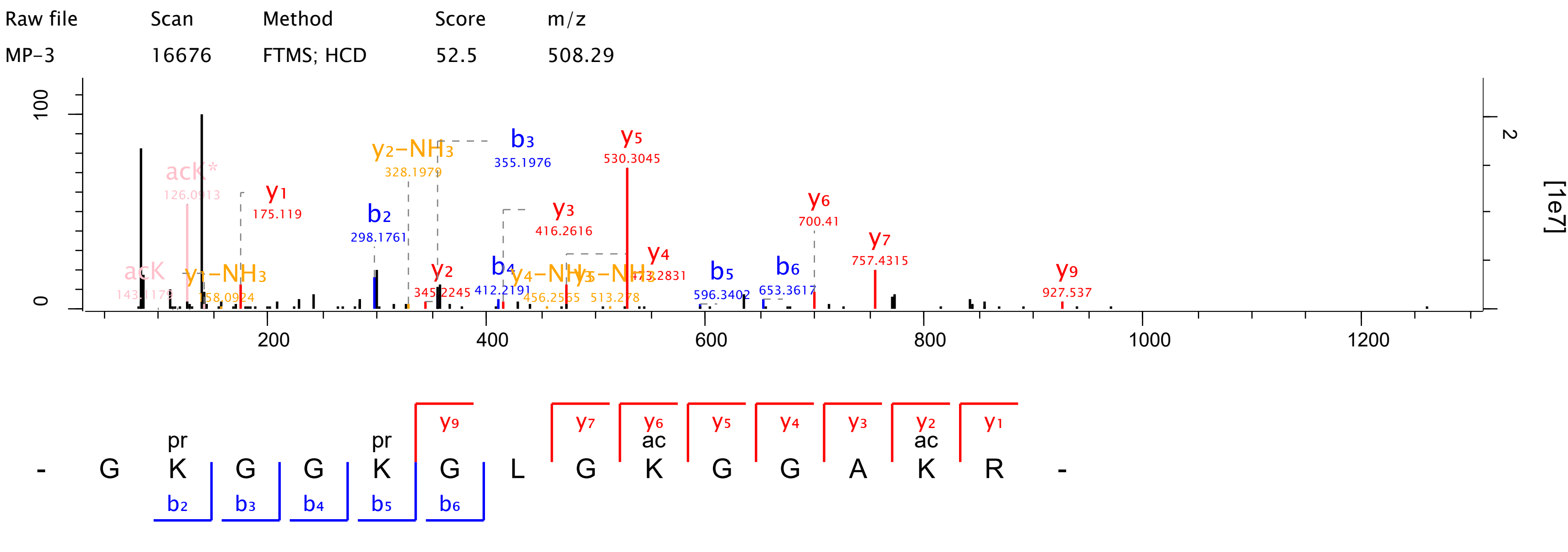

Raw file | Scan | Method | Score | m/z
MP-3 | 16676 | FTMS; HCD | 52.5 | 508.29
100
0
[1e7]
2
200
400
600
800
1000
1200
ack*
126.0913
acK
143.1179
y1
175.119
y1−NH3
158.0924
b2
298.1761
y2−NH3
328.1979
b3
355.1976
y2
345.2245
b4
412.2191
y3
416.2616
y4−NH3
456.2565
y5
530.3045
y5−NH3
513.278
y4
473.2831
b5
596.3402
b6
653.3617
y6
700.41
y7
757.4315
y9
927.537
- G K G G K G L G K G G A K R -
pr pr ac ac
y9 y7 y6 y5 y4 y3 y2 y1
b2 b3 b4 b5 b6

Raw file	Scan	Method	Score	m/z
TP+3	11946	FTMS; HCD	46.28	733.92

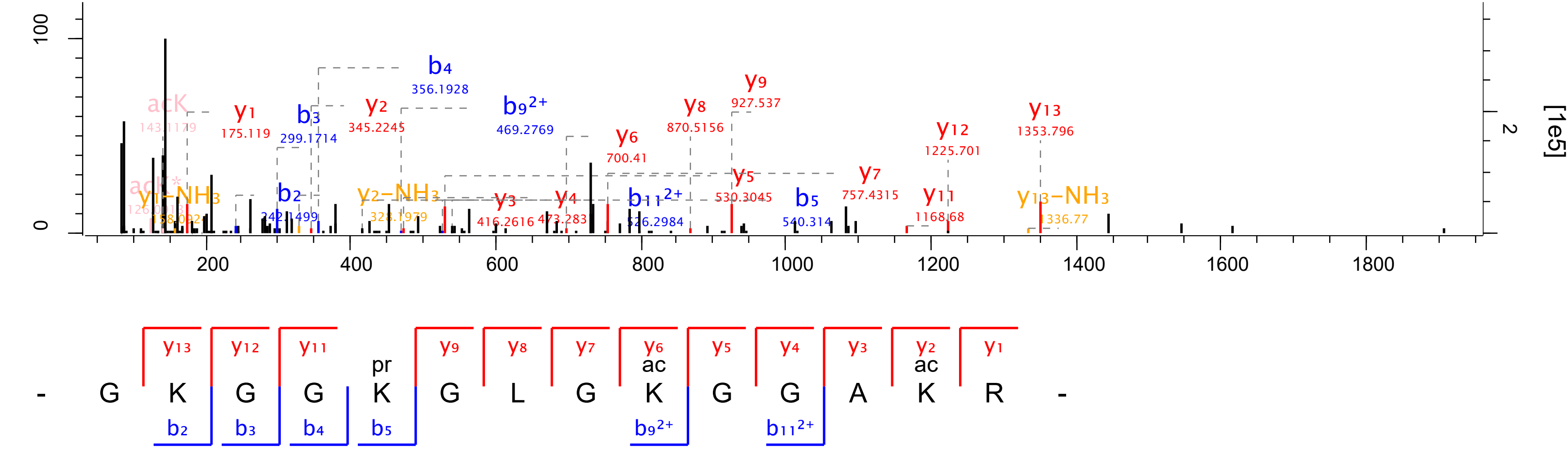

Raw file	Scan	Method	Score	m/z
MP-1	11116	FTMS; HCD	45.21	484.95

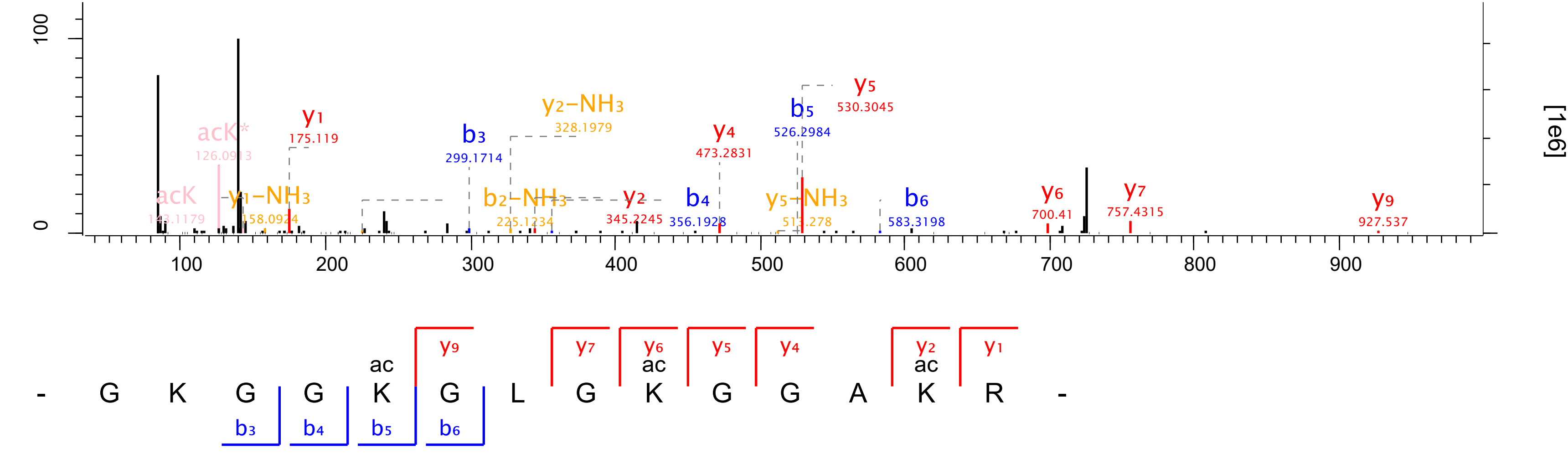

Raw file	Scan	Method	Score	m/z
MP-1	16273	FTMS; HCD	47.04	755.43

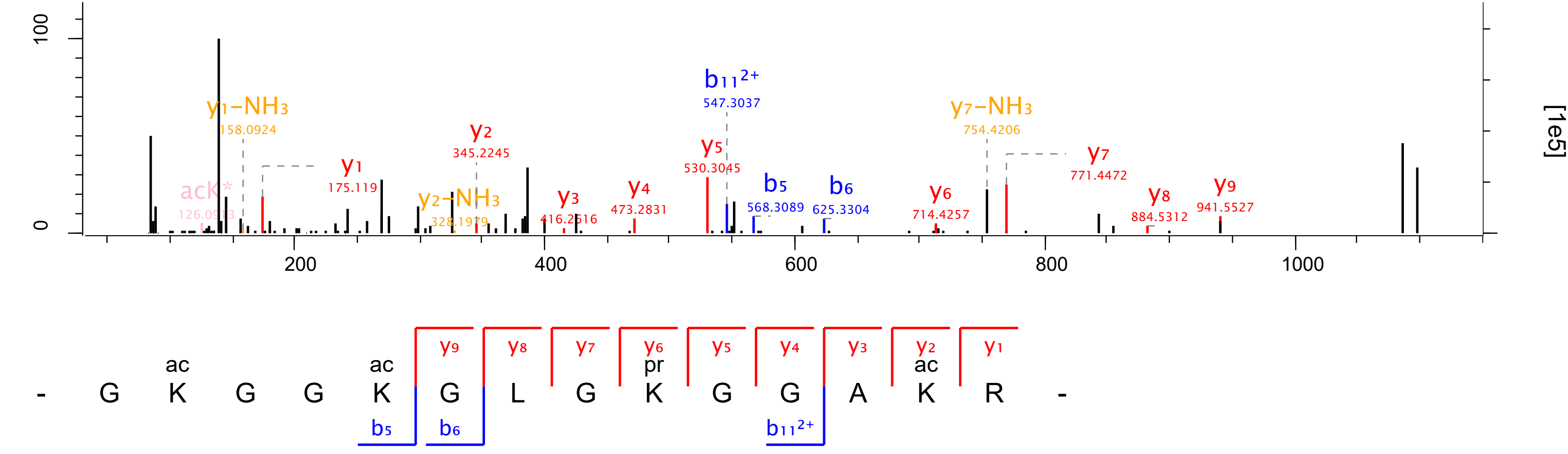

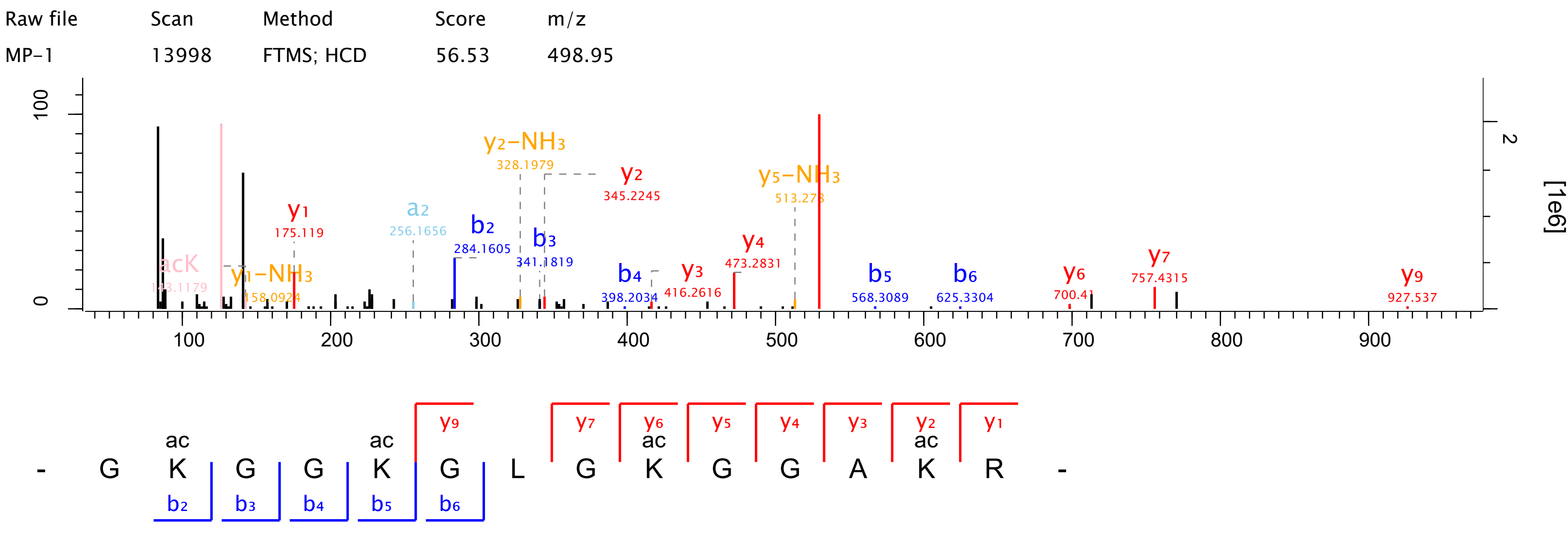

Raw file
Scan
Method
Score
m/z
MP-1
13998
FTMS; HCD
56.53
498.95
100
0
[1e6]
2
acK
143.1179
y1-NH3
158.0924
y1
175.119
a2
256.1656
b2
284.1605
y2-NH3
328.1979
b3
341.1819
y2
345.2245
b4
398.2034
y3
416.2616
y4
473.2831
y5-NH3
513.278
b5
568.3089
b6
625.3304
y6
700.41
y7
757.4315
y9
927.537
100
200
300
400
500
600
700
800
900
- G K G G K G L G K G G A K R -
ac
ac
ac
ac
y9 y7 y6 y5 y4 y3 y2 y1
b2 b3 b4 b5 b6

Raw file	Scan	Method	Score	m/z
TP+3	13141	FTMS; HCD	73.39	740.93

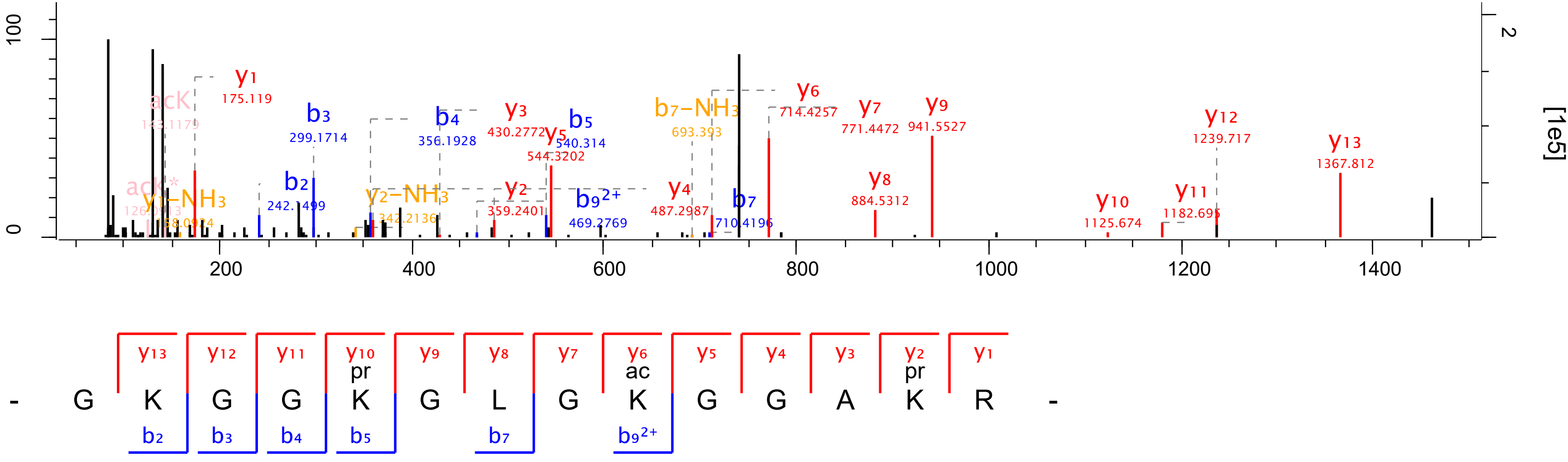

Raw file	Scan	Method	Score	m/z
MP-1	17636	FTMS; HCD	110.15	768.95

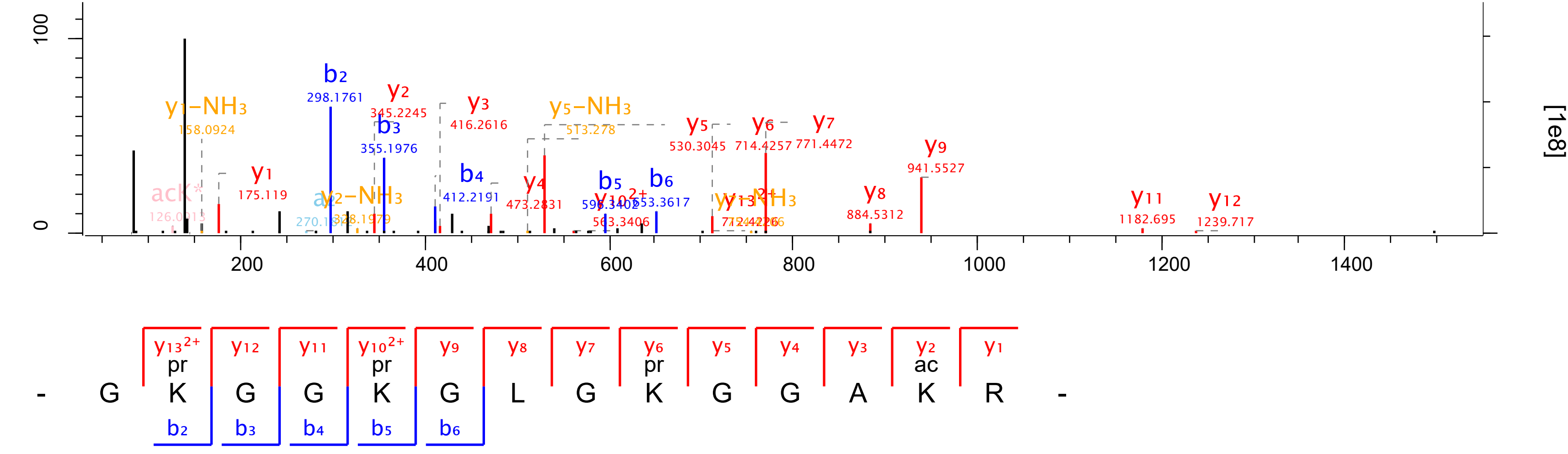

Raw file	Scan	Method	Score	m/z
TR-1	9438	FTMS; HCD	98.59	712.92

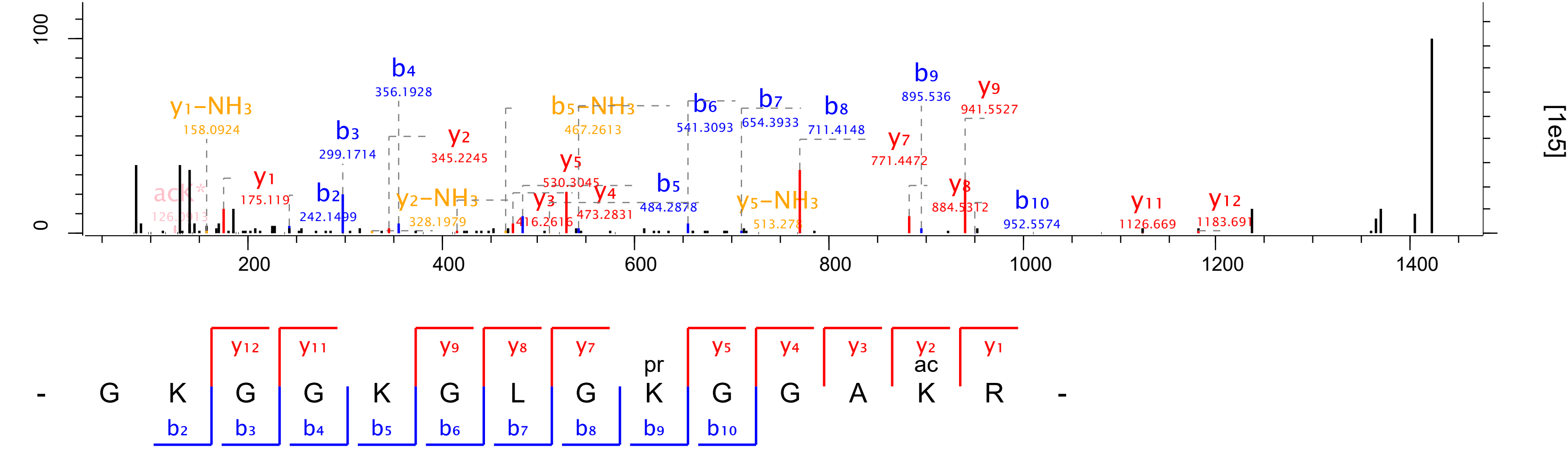

Raw file	Scan	Method	Score	m/z
TR+2	6342	FTMS; HCD	43.63	457.76

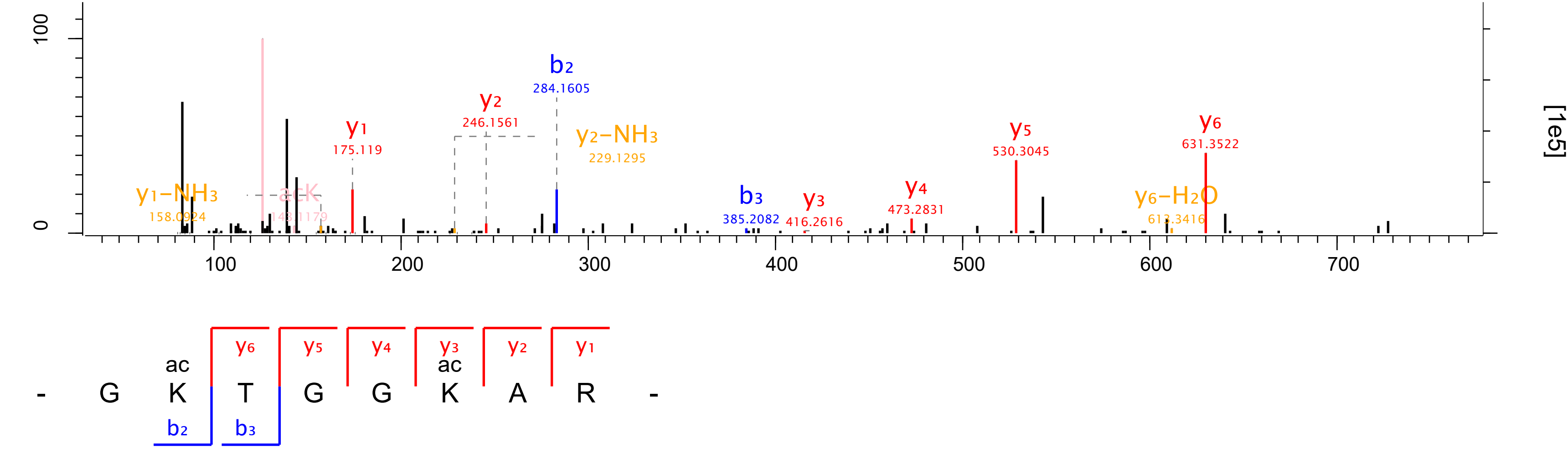

Raw file	Scan	Method	Score	m/z
TR-2	9877	FTMS; HCD	87.32	785.45

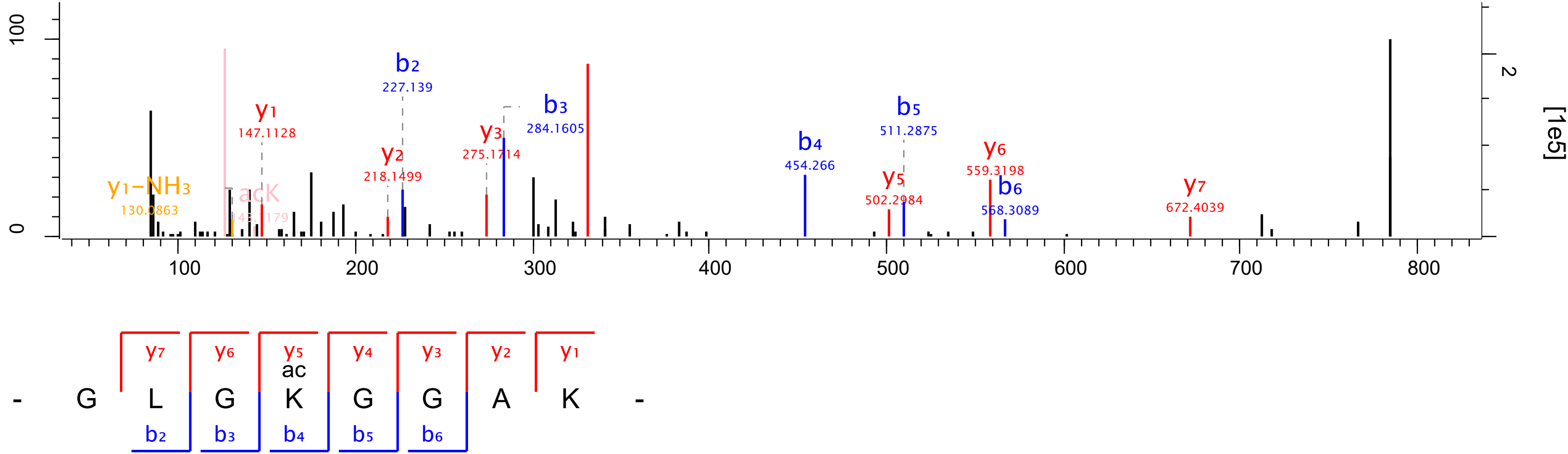

Raw file	Scan	Method	Score	m/z
TR-1	13859	FTMS; HCD	87.64	499.29

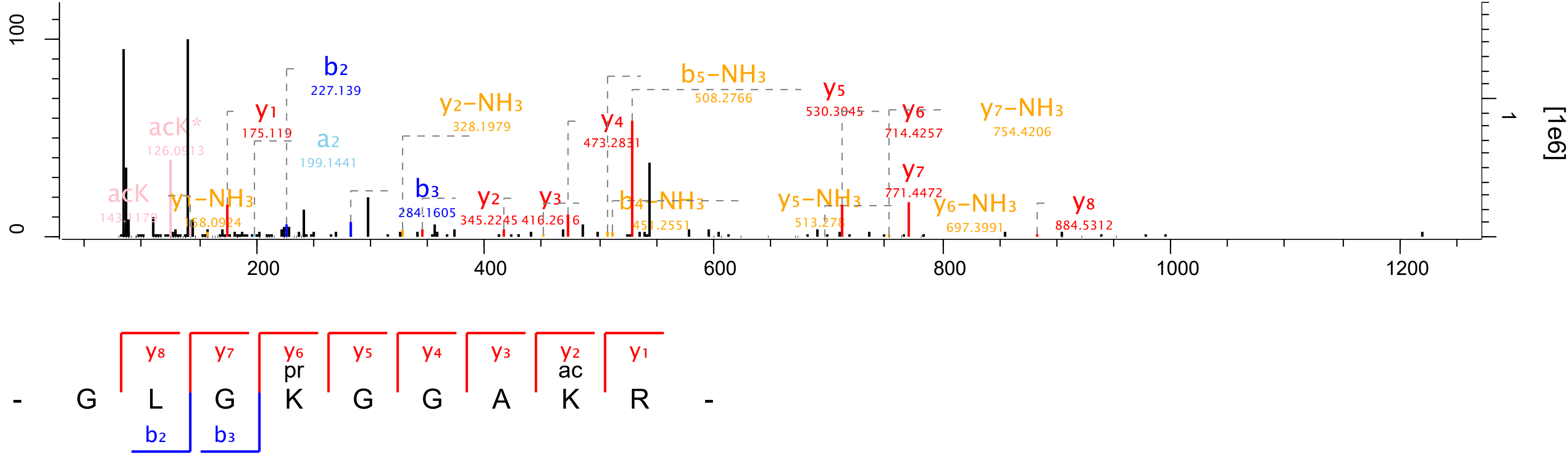

Raw file	Scan	Method	Score	m/z
TP-1	10278	FTMS; HCD	48.95	650.38

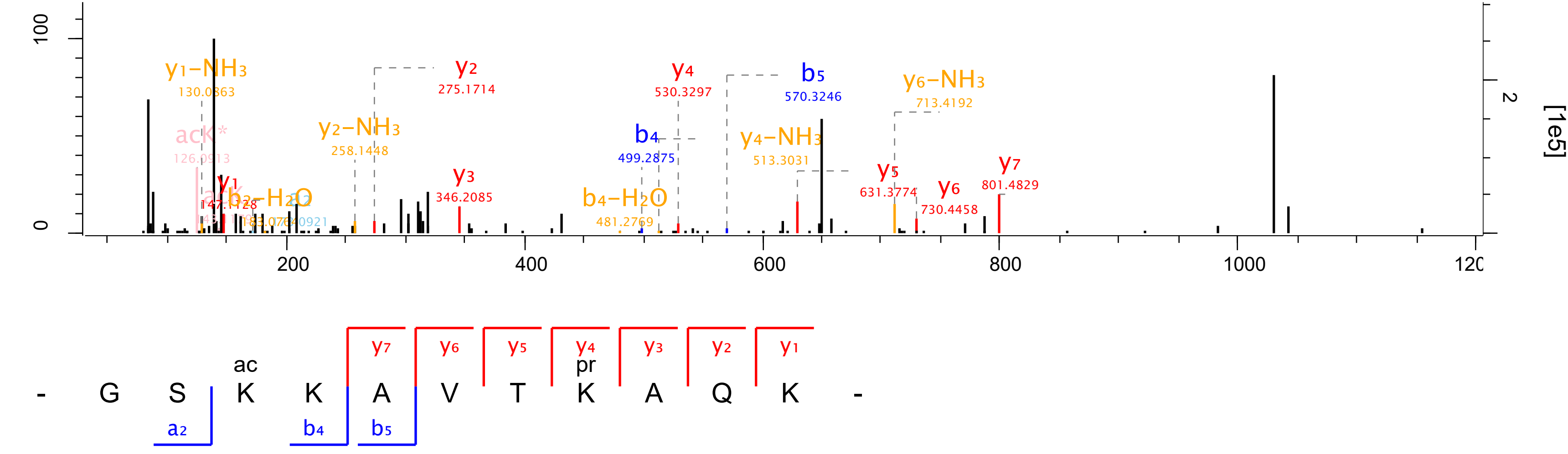

Raw file	Scan	Method	Score	m/z
TP+2	34982	FTMS; HCD	63.62	625.84

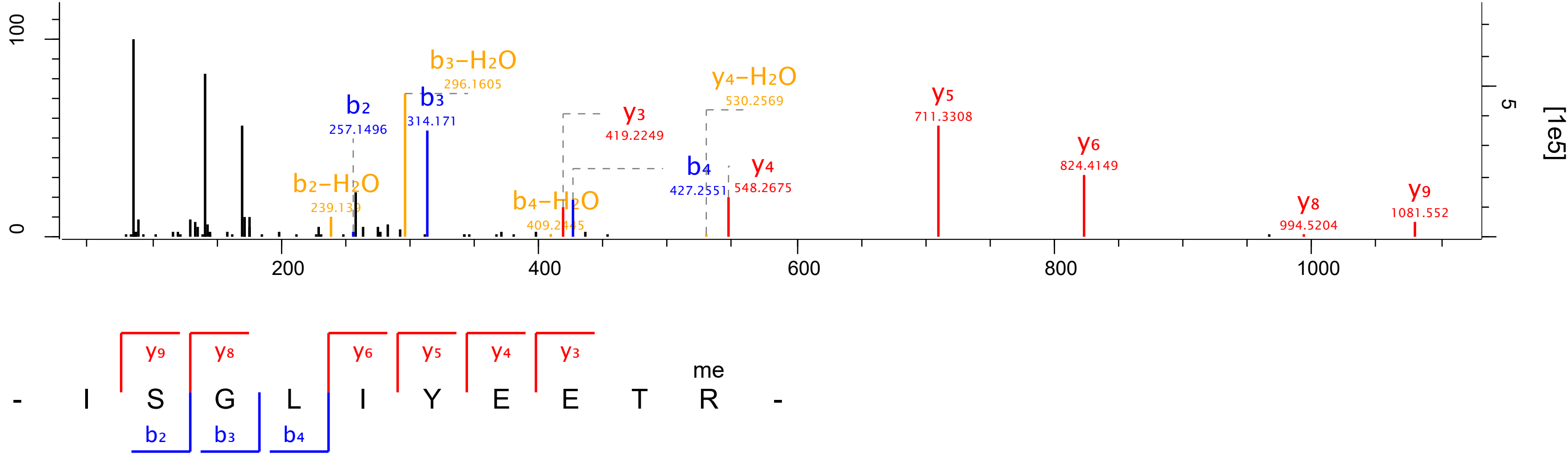

Raw file	Scan	Method	Score	m/z
TP-1	6228	FTMS; HCD	61.11	442.6

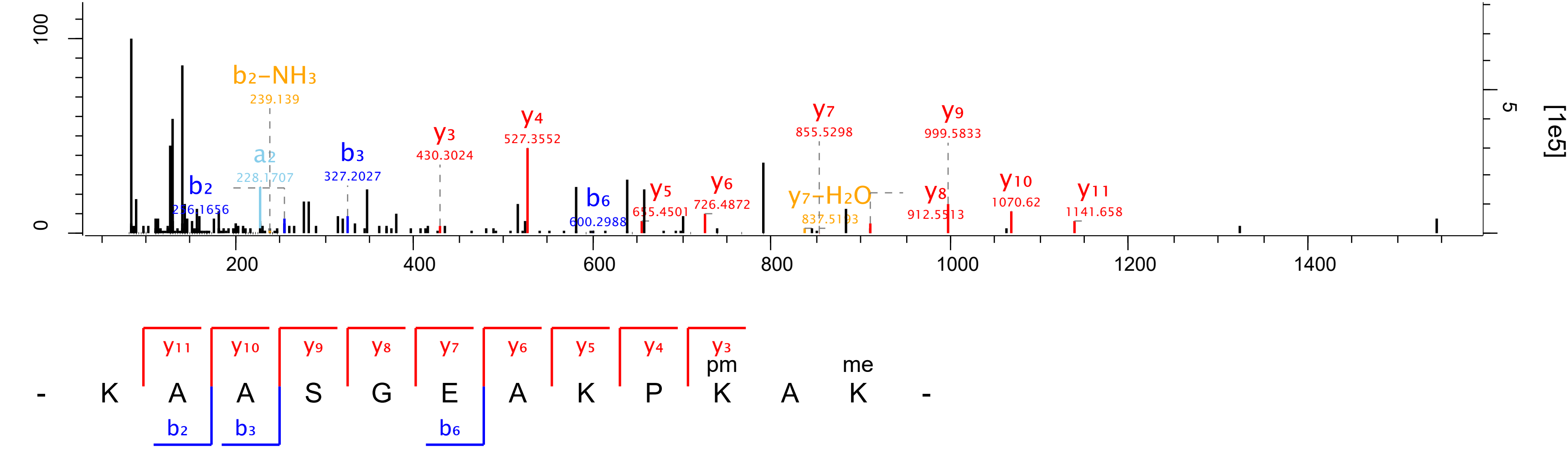

Raw file	Scan	Method	Score	m/z
TP+2	34235	FTMS; HCD	95.2	774.77

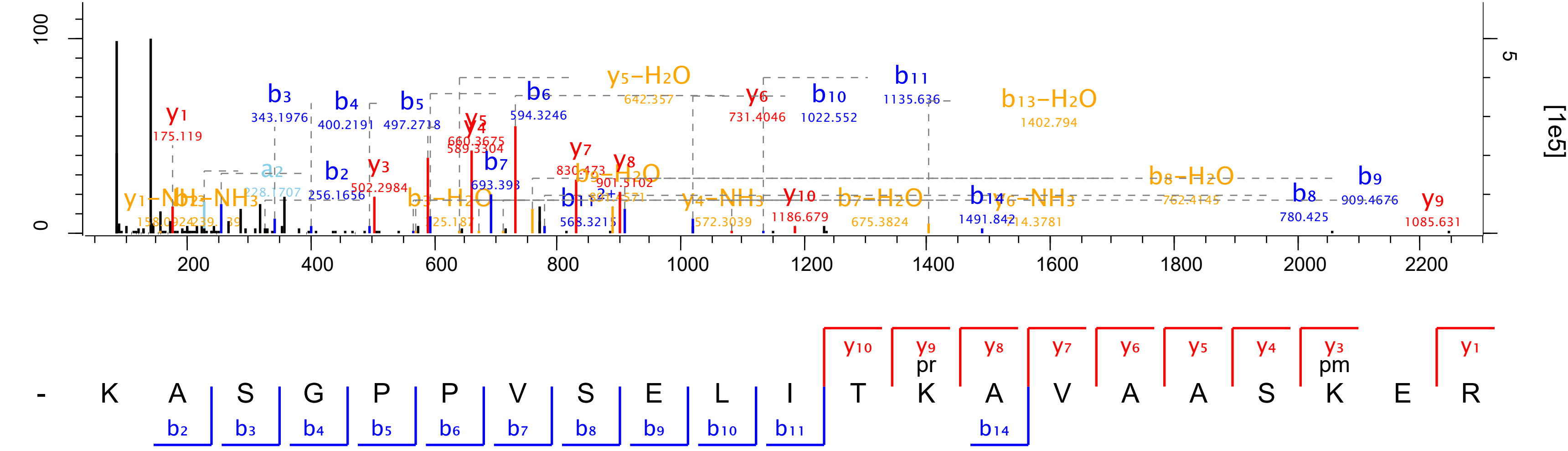

Raw file	Scan	Method	Score	m/z
TP+2	34784	FTMS; HCD	92.1	779.45

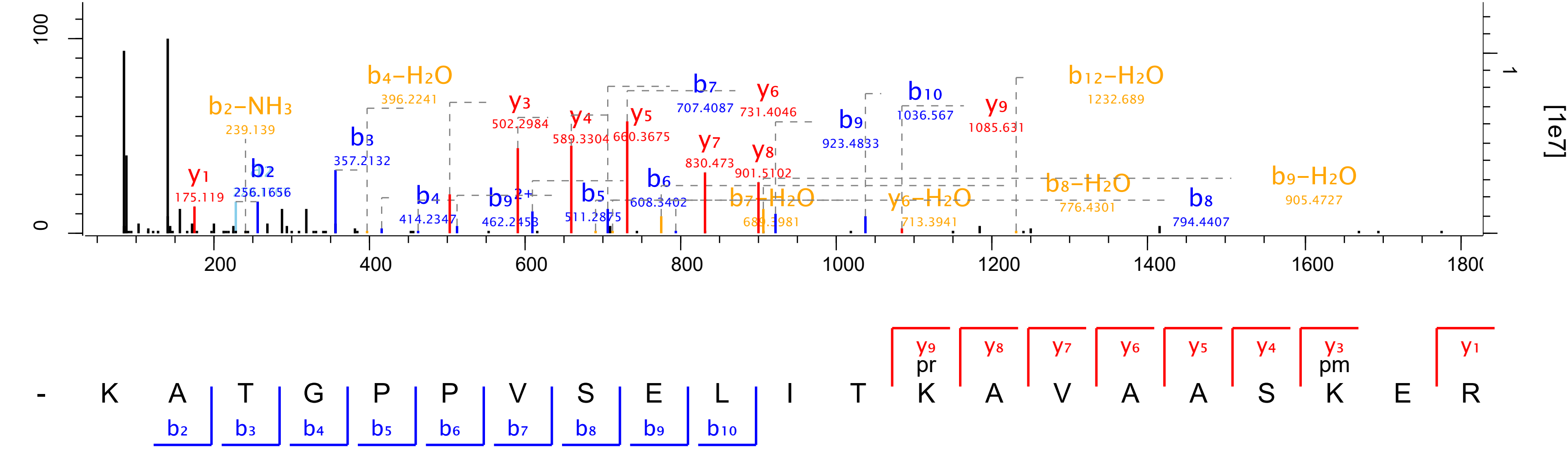

Raw file	Scan	Method	Score	m/z
TP-2	5974	FTMS; HCD	136.99	486.3

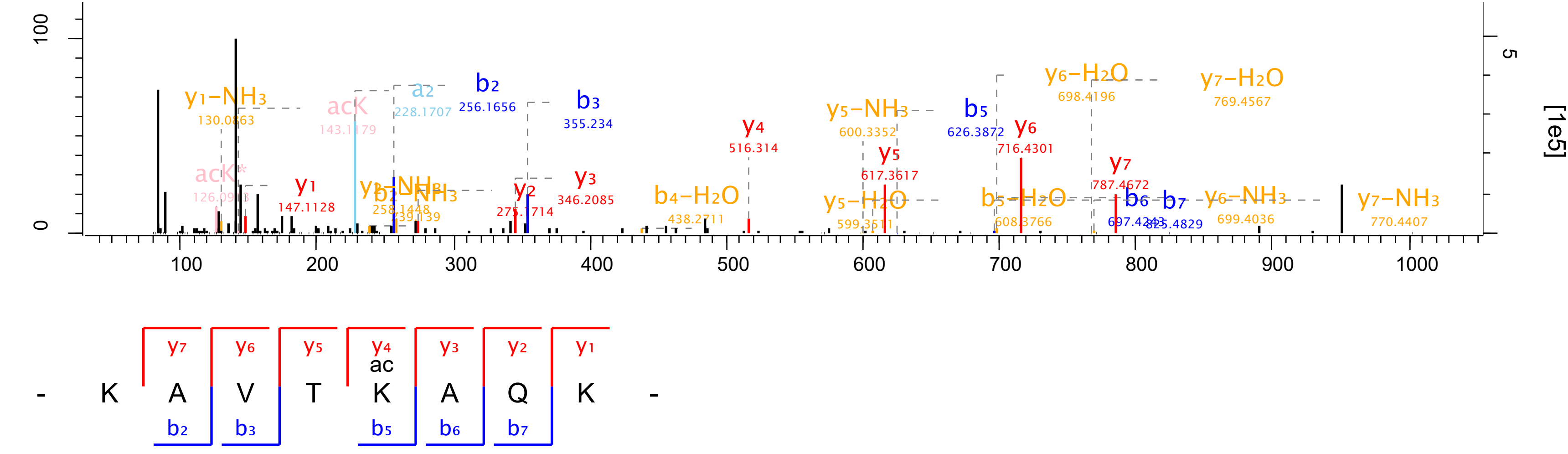

Raw file	Scan	Method	Score	m/z
TP-1	9258	FTMS; HCD	63.62	590.4

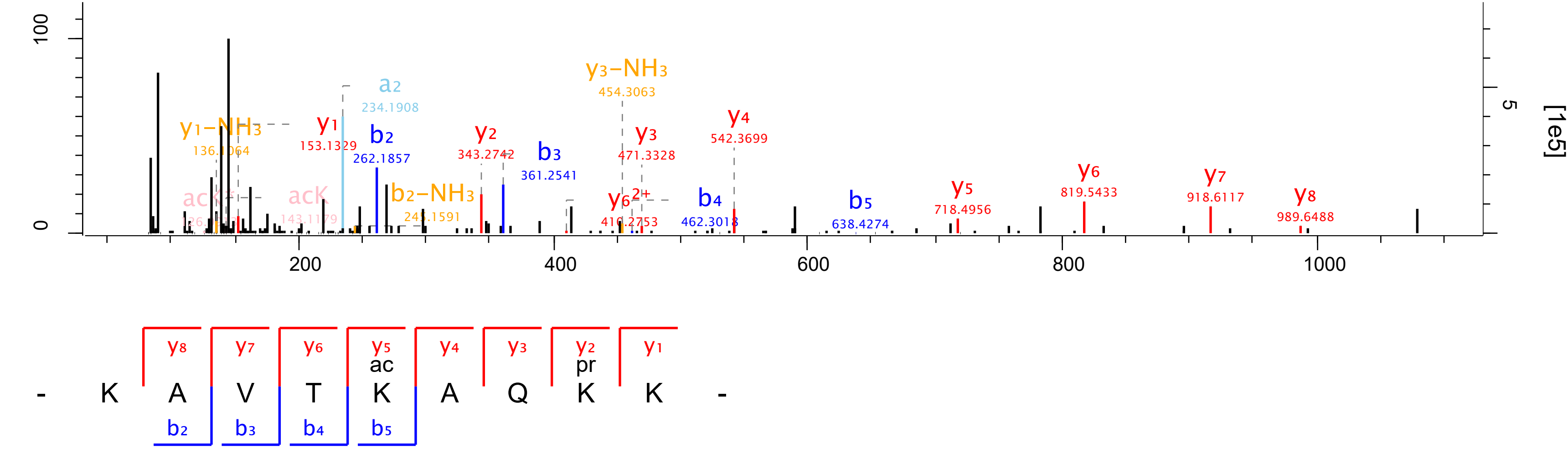

Raw file	Scan	Method	Score	m/z
TP-1	12606	FTMS; HCD	75.1	756.44

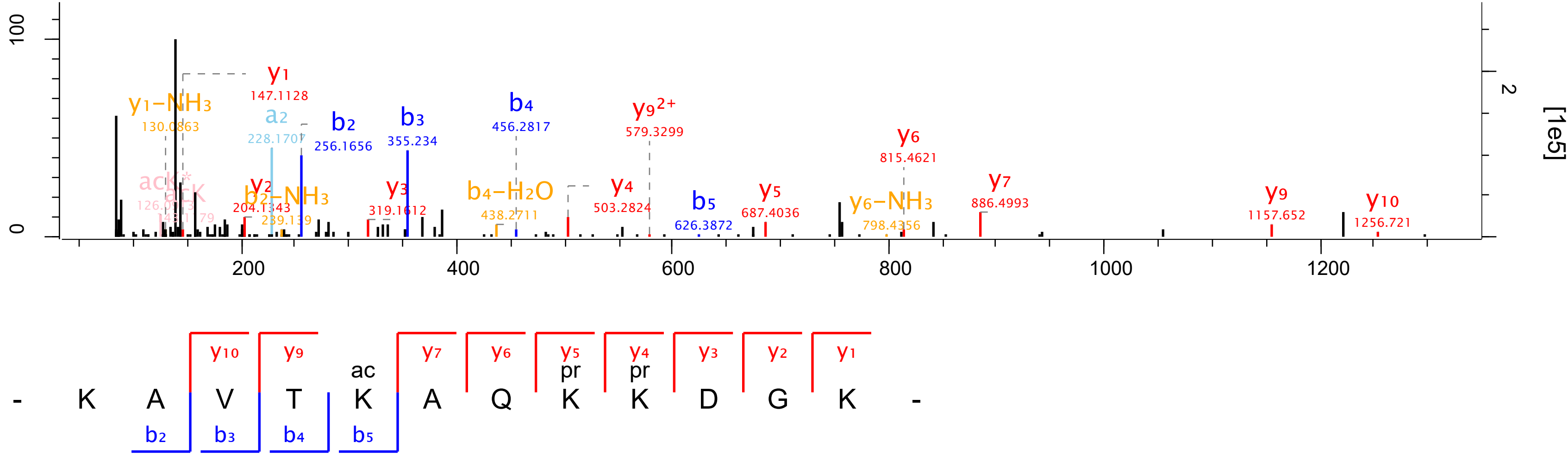

Raw file	Scan	Method	Score	m/z
TP+2	7637	FTMS; HCD	56.55	462.23

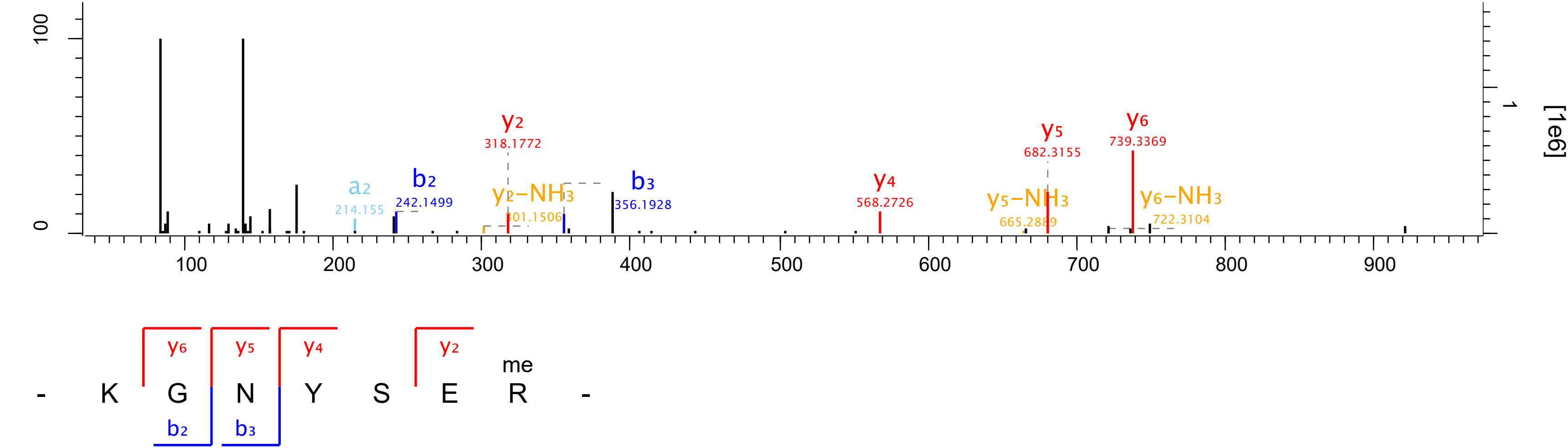

Raw file	Scan	Method	Score	m/z
TP-3	7234	FTMS; HCD	48.79	543.83

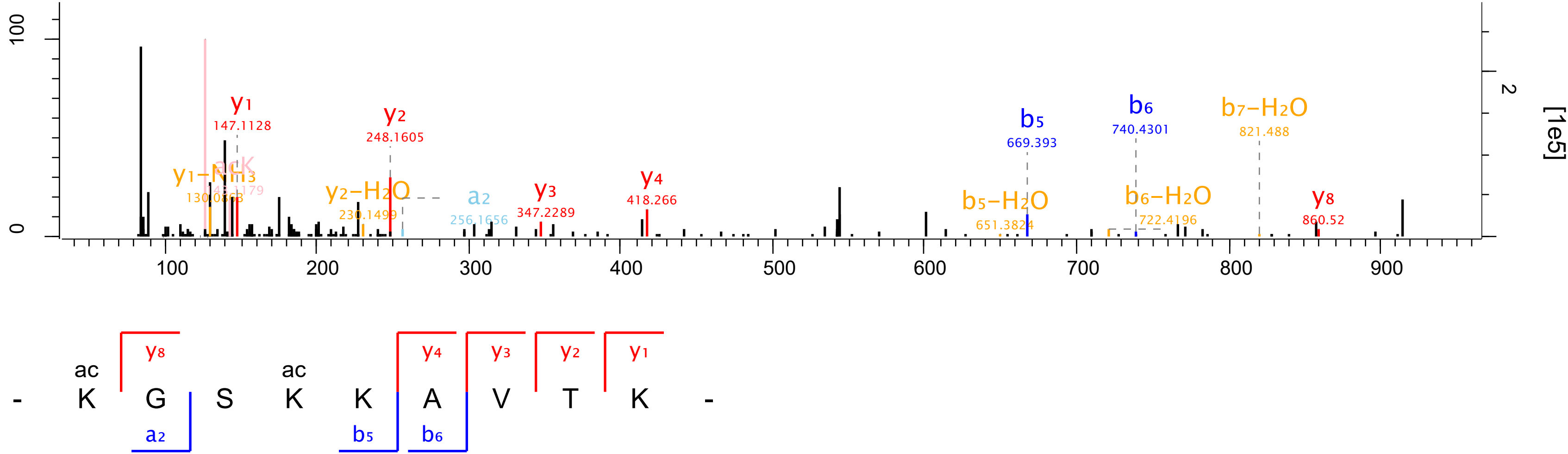

Raw file	Scan	Method	Score	m/z
TP-1	14677	FTMS; HCD	41.4	578.85

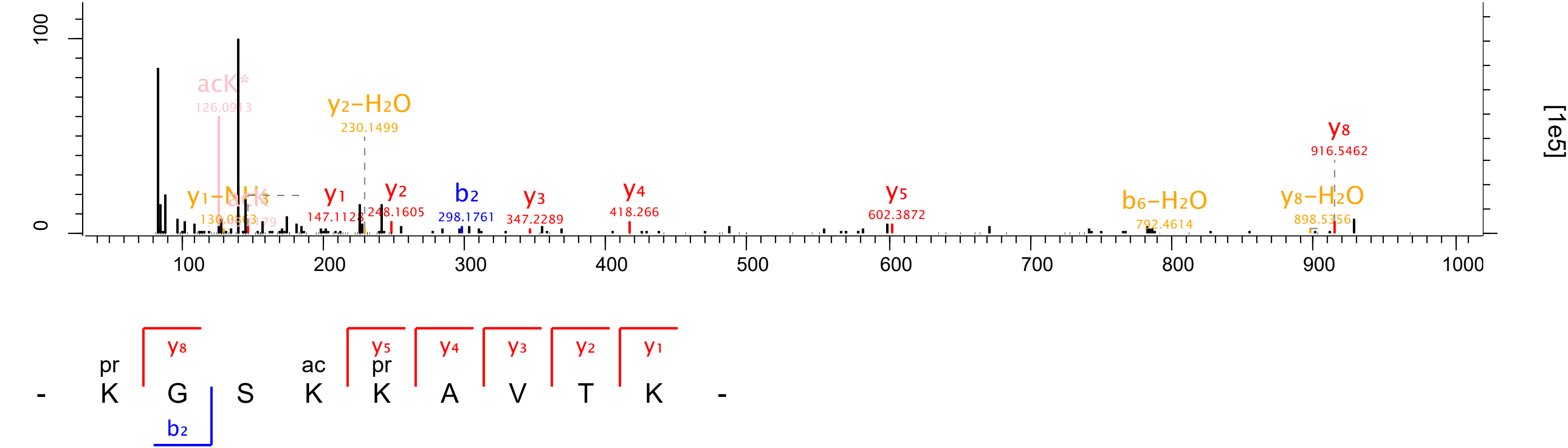

Raw file	Scan	Method	Score	m/z
MR+3	8277	FTMS; HCD	136.5	550.84

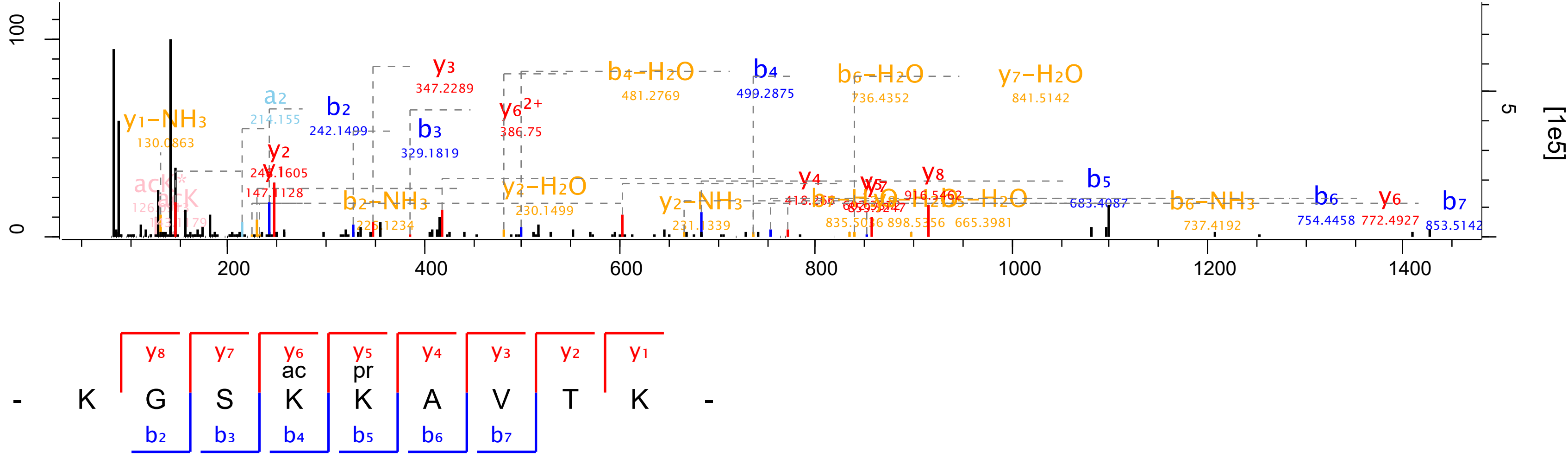

Raw file	Scan	Method	Score	m/z
TP-1	12228	FTMS; HCD	108.91	757.49

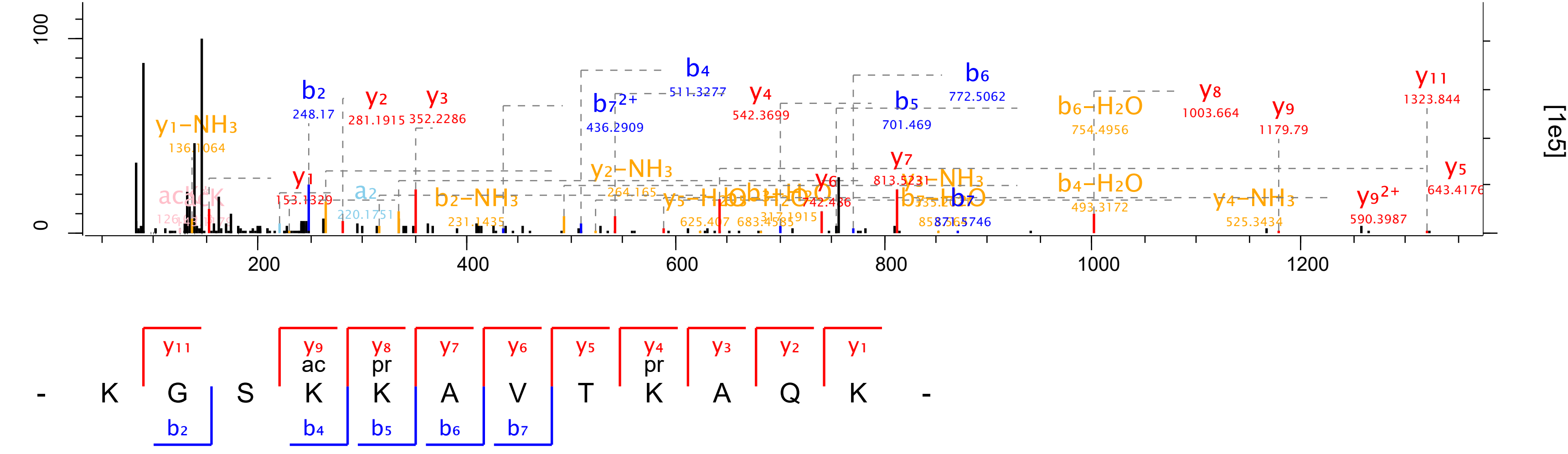

Raw file	Scan	Method	Score	m/z
TR+1	9841	FTMS; HCD	109.29	542.32

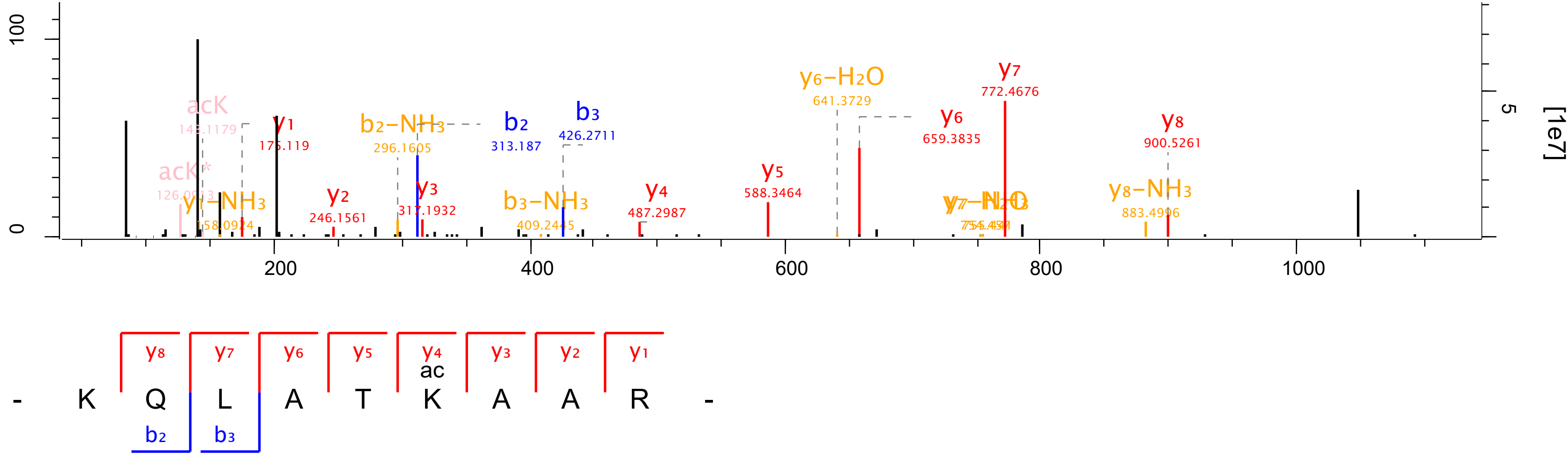

Raw file	Scan	Method	Score	m/z
TP-1	14069	FTMS; HCD	56.01	556.84

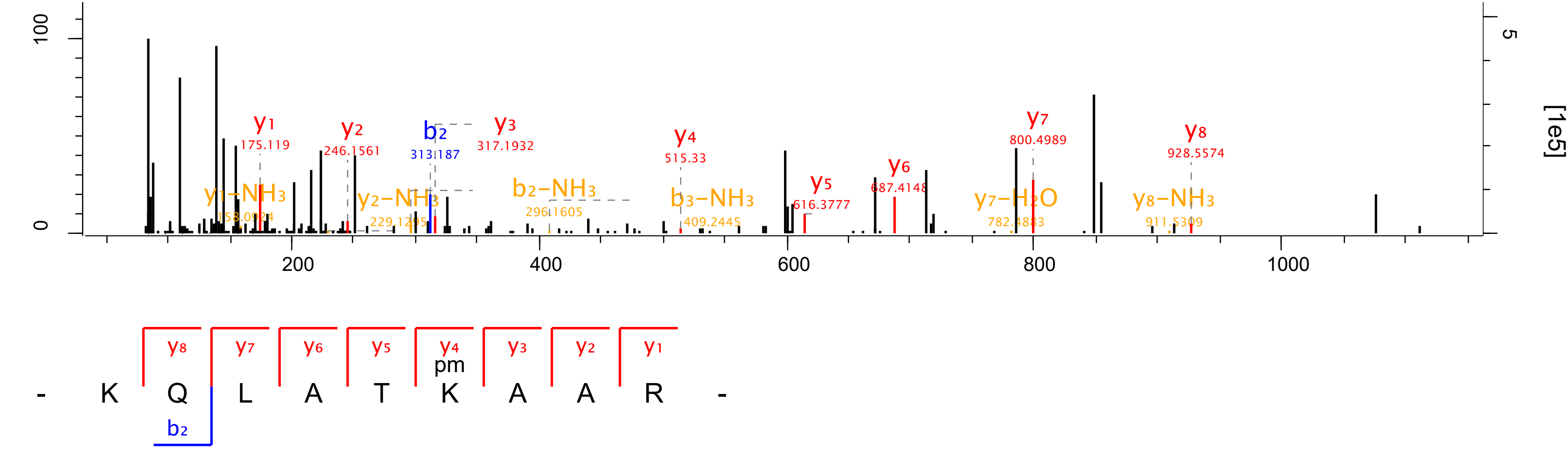

Raw file	Scan	Method	Score	m/z
MR-2	23631	FTMS; HCD	70.03	584.85

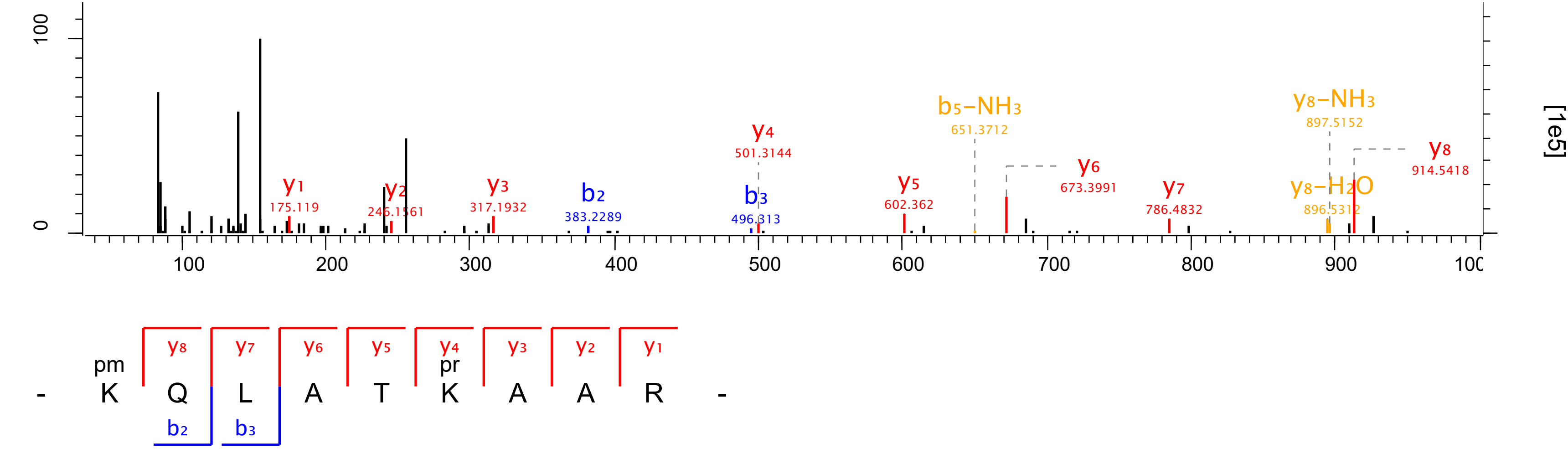

Raw file	Scan	Method	Score	m/z
TR-2	5666	FTMS; HCD	128.08	505.81

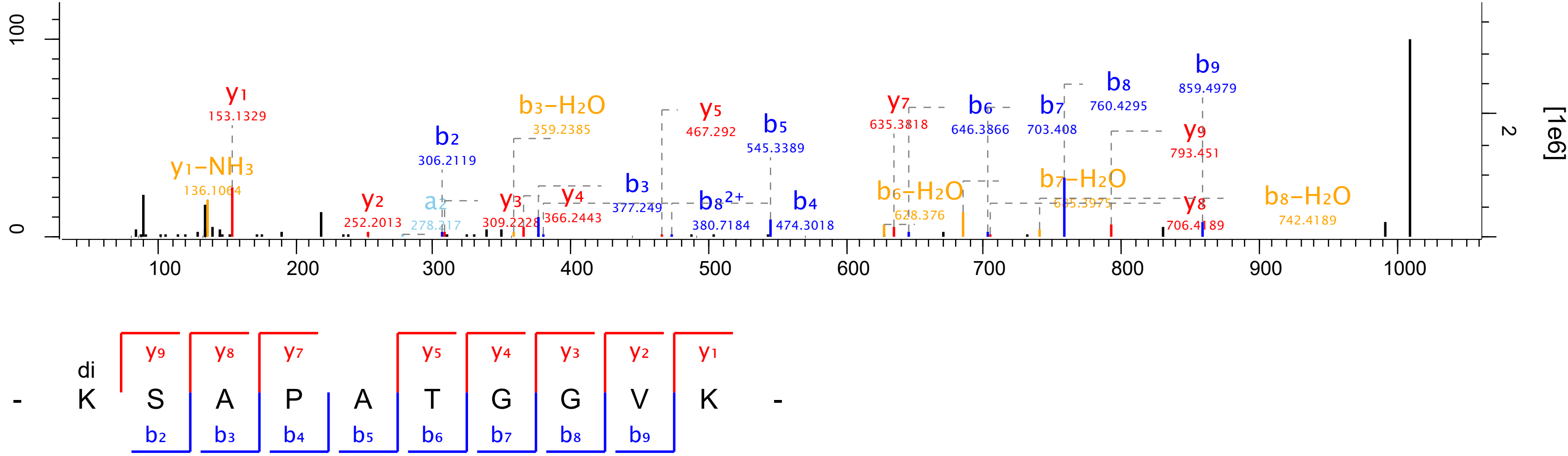

Raw file	Scan	Method	Score	m/z
TP-3	8792	FTMS; HCD	72.61	985.57

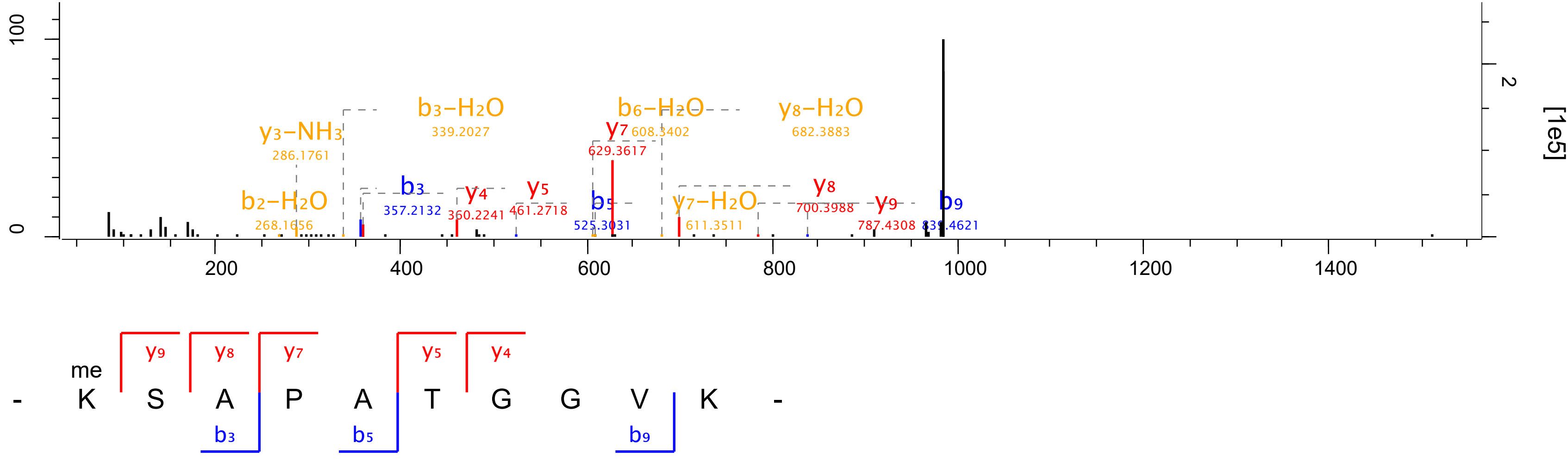

Raw file	Scan	Method	Score	m/z
MP-2	14158	FTMS; HCD	63.82	1041.59

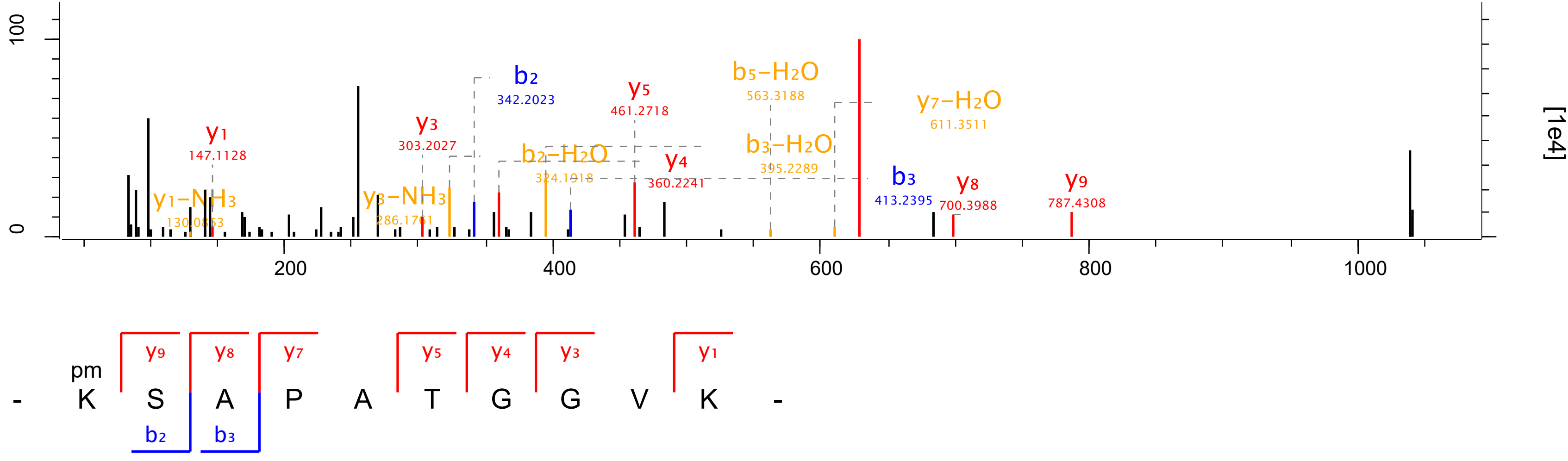

Raw file	Scan	Method	Score	m/z
TP-2	5440	FTMS; HCD	59.84	507.3

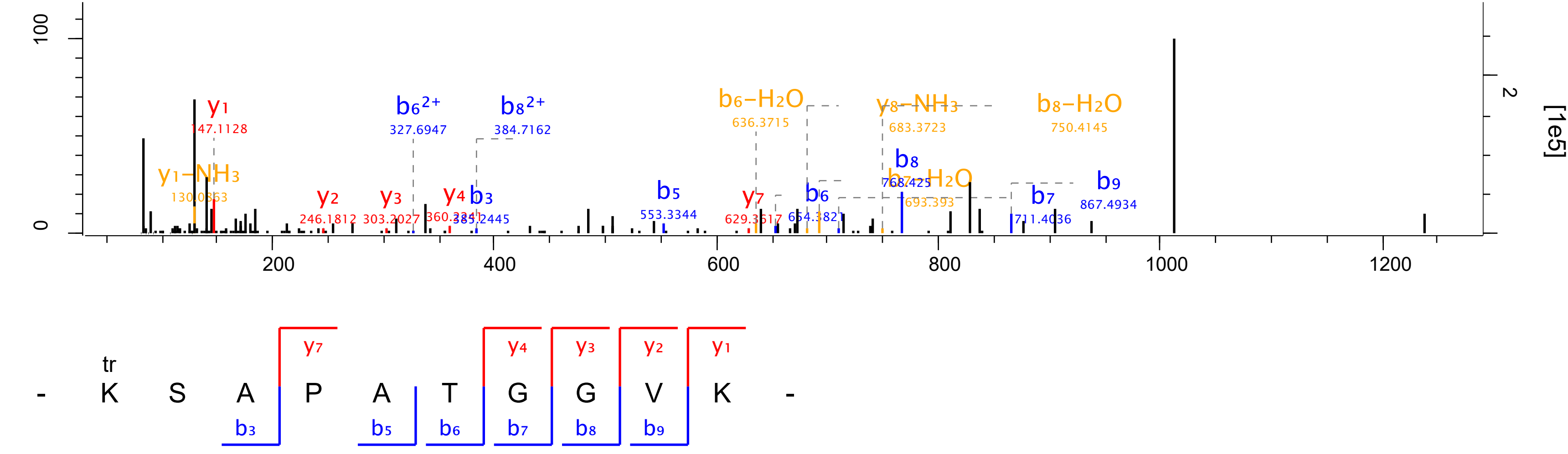

Raw file	Scan	Method	Score	m/z
MR+2	14625	FTMS; HCD	70.44	544.65

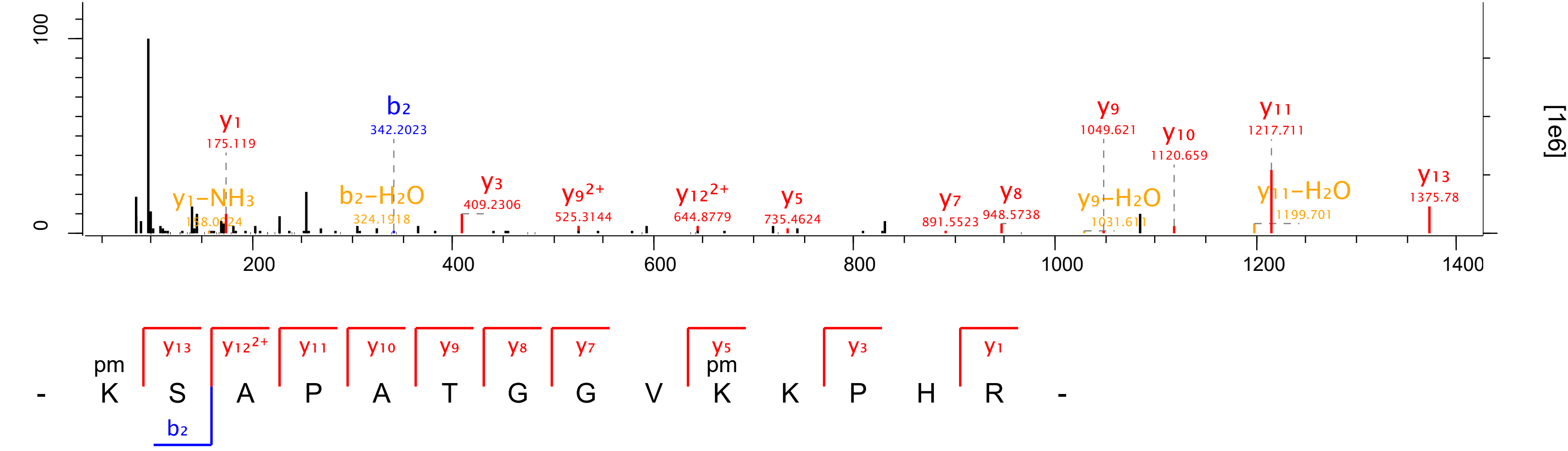

Raw file	Scan	Method	Score	m/z
TP+3	4364	FTMS; HCD	143.93	516.65

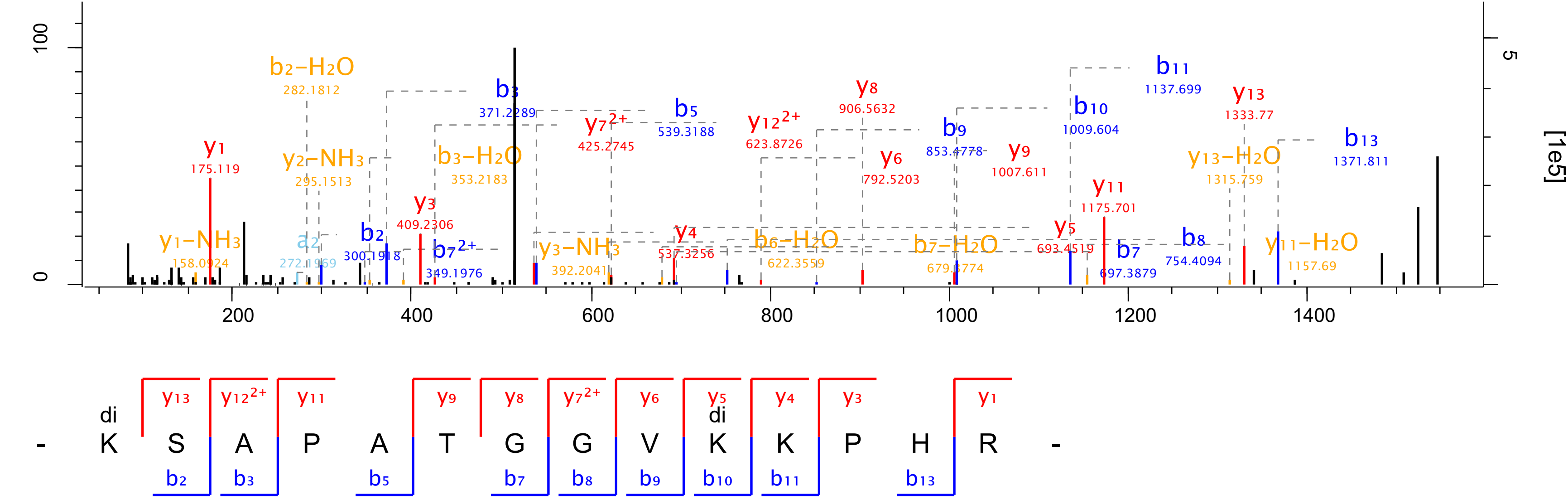

Raw file	Scan	Method	Score	m/z
MR-3	4712	FTMS; HCD	106.32	380.23

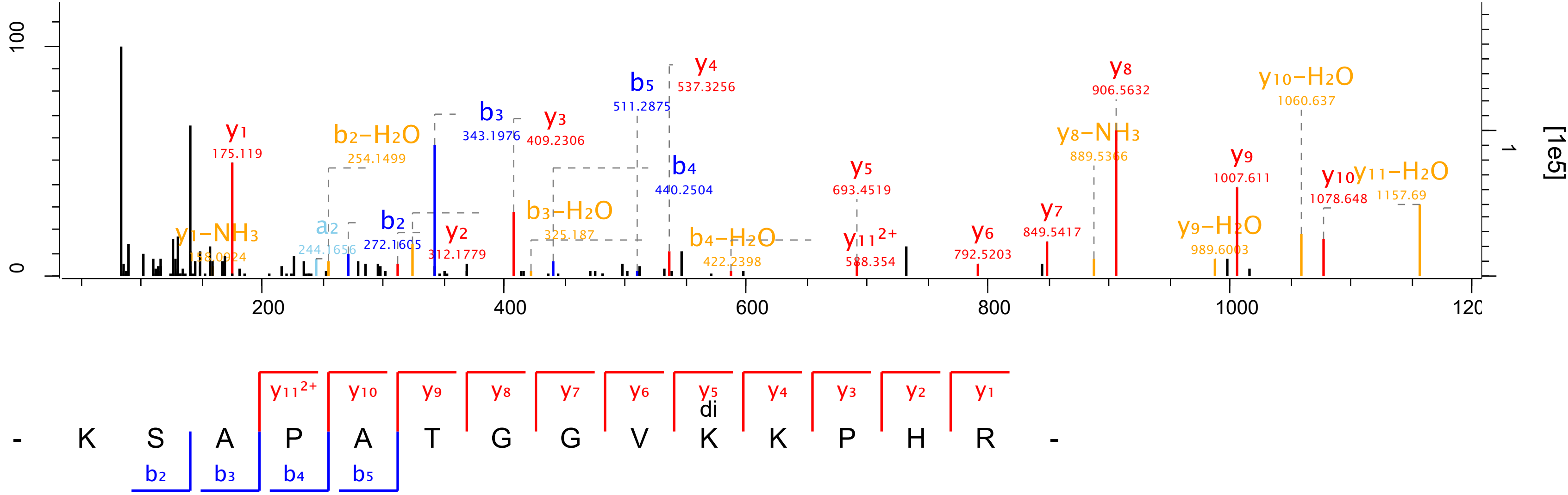

Raw file	Scan	Method	Score	m/z
TP-1	17741	FTMS; HCD	58.27	837.48

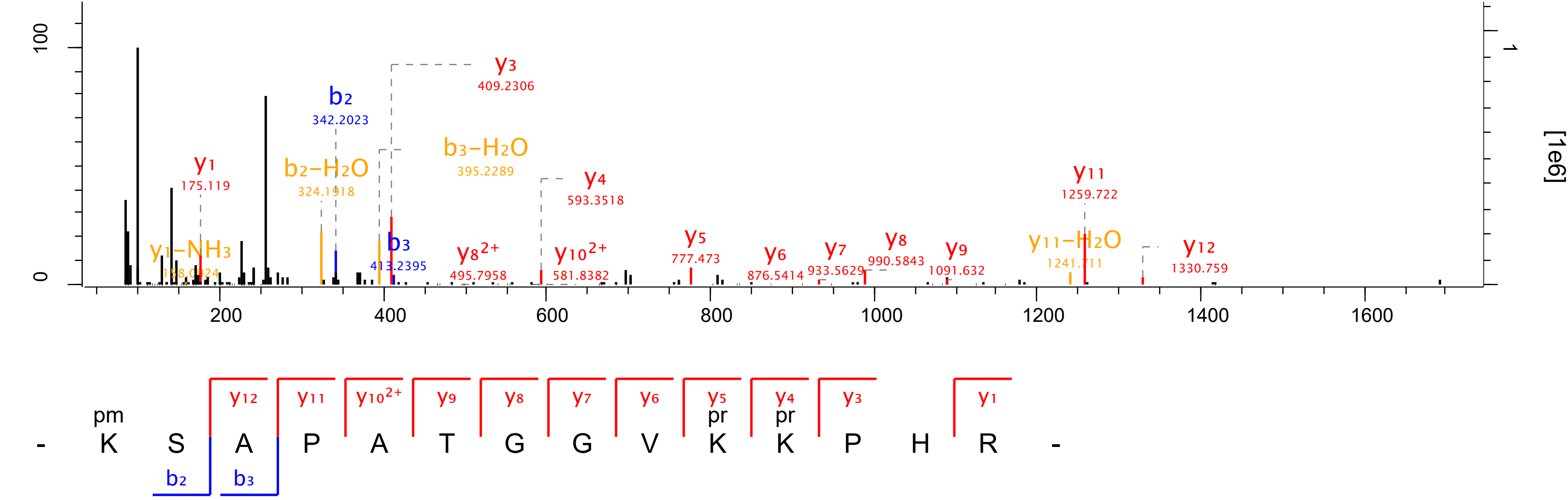

Raw file	Scan	Method	Score	m/z
TP-2	6386	FTMS; HCD	97.69	766.46

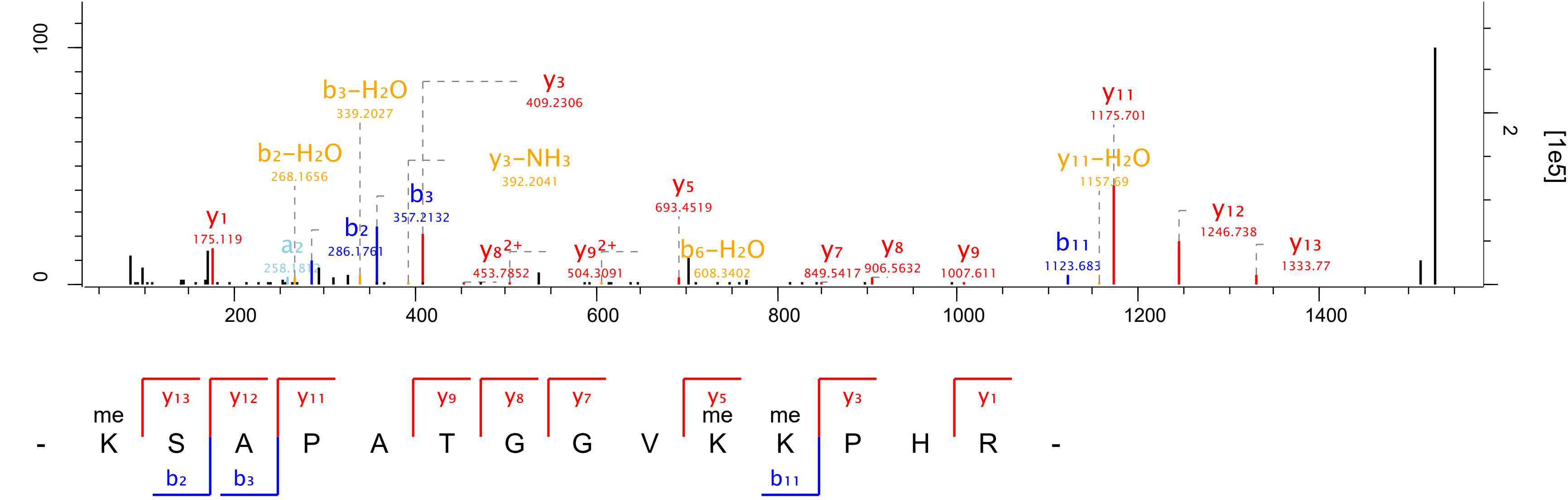

Raw file	Scan	Method	Score	m/z
TP+2	9133	FTMS; HCD	95.26	520.64

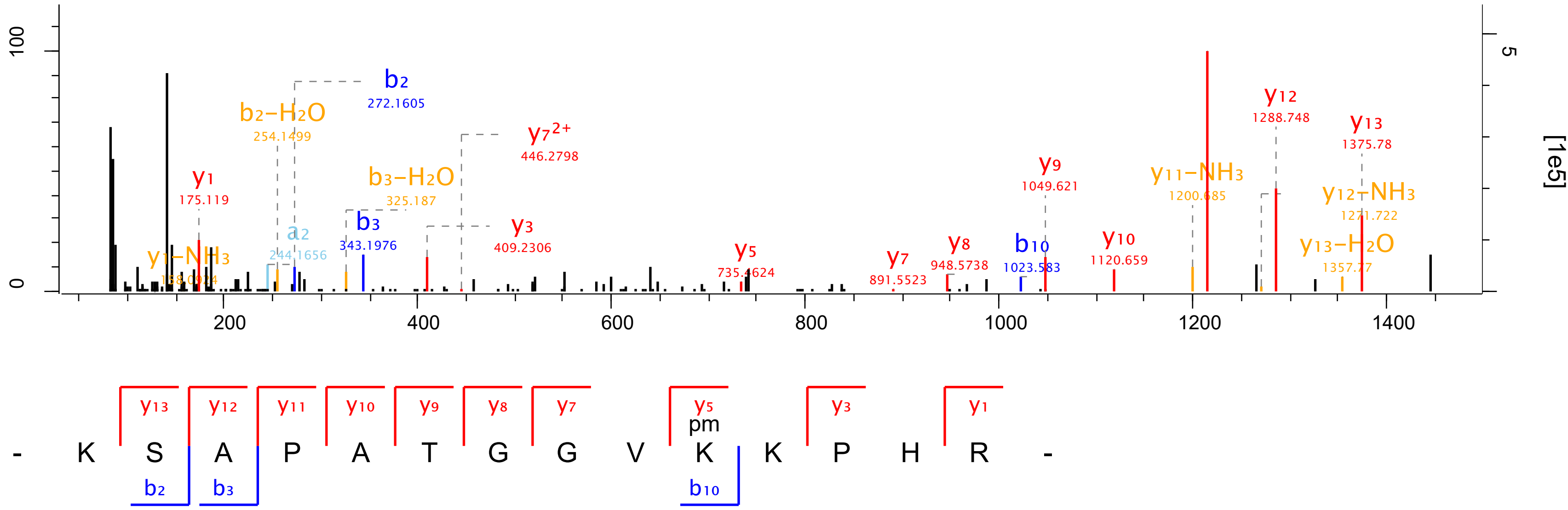

Raw file	Scan	Method	Score	m/z
TP+3	18648	FTMS; HCD	94.09	844.49

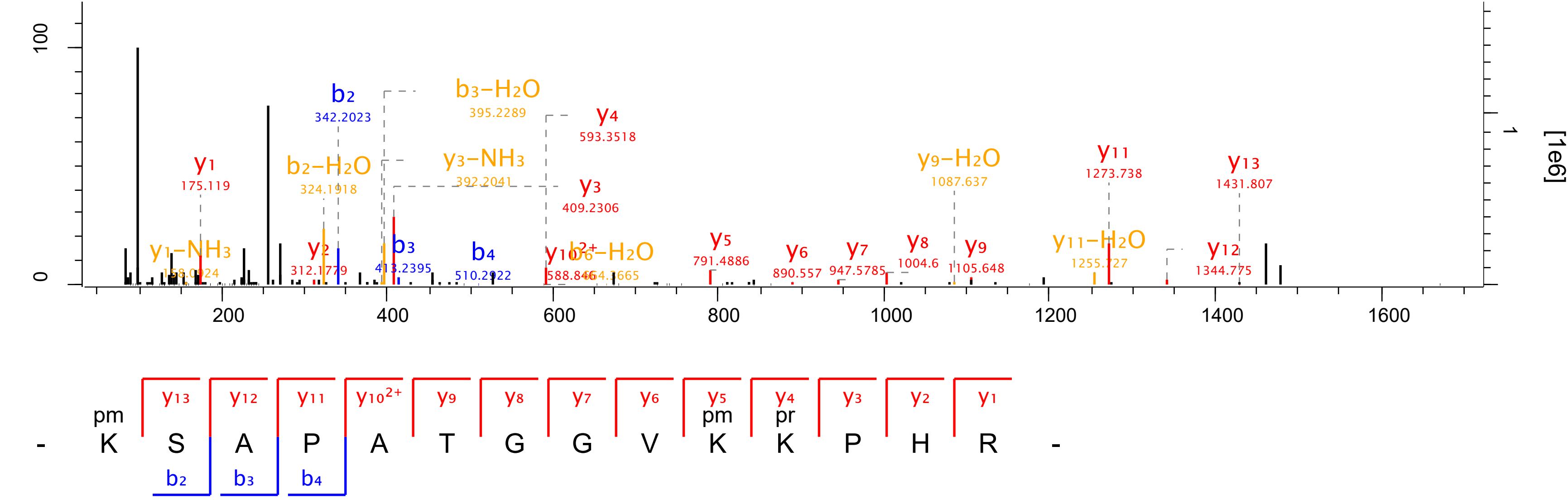

Raw file	Scan	Method	Score	m/z
TR-3	14973	FTMS; HCD	62.16	548.64

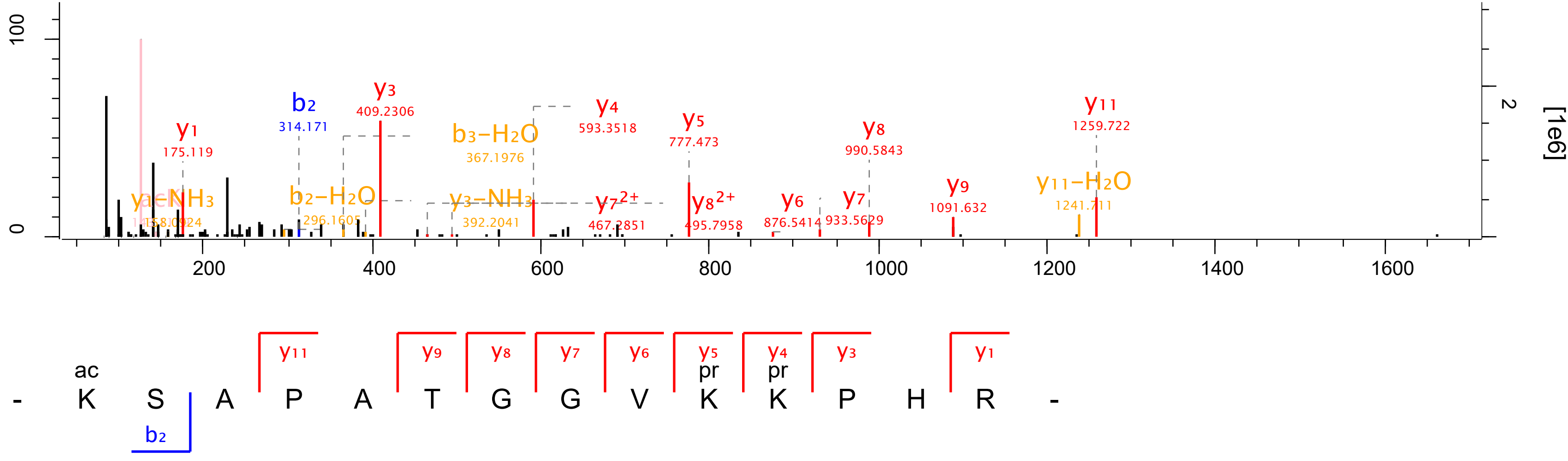

Raw file	Scan	Method	Score	m/z
MR+3	9214	FTMS; HCD	148.96	525.31

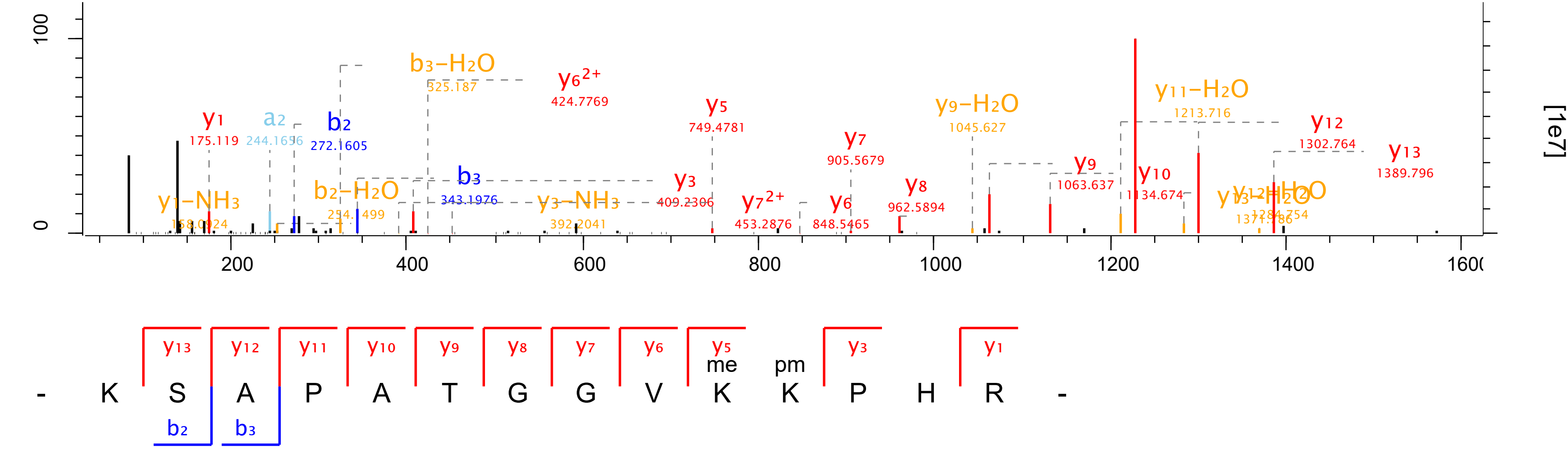

Raw file	Scan	Method	Score	m/z
MP-3	13392	FTMS; HCD	89.23	538.98

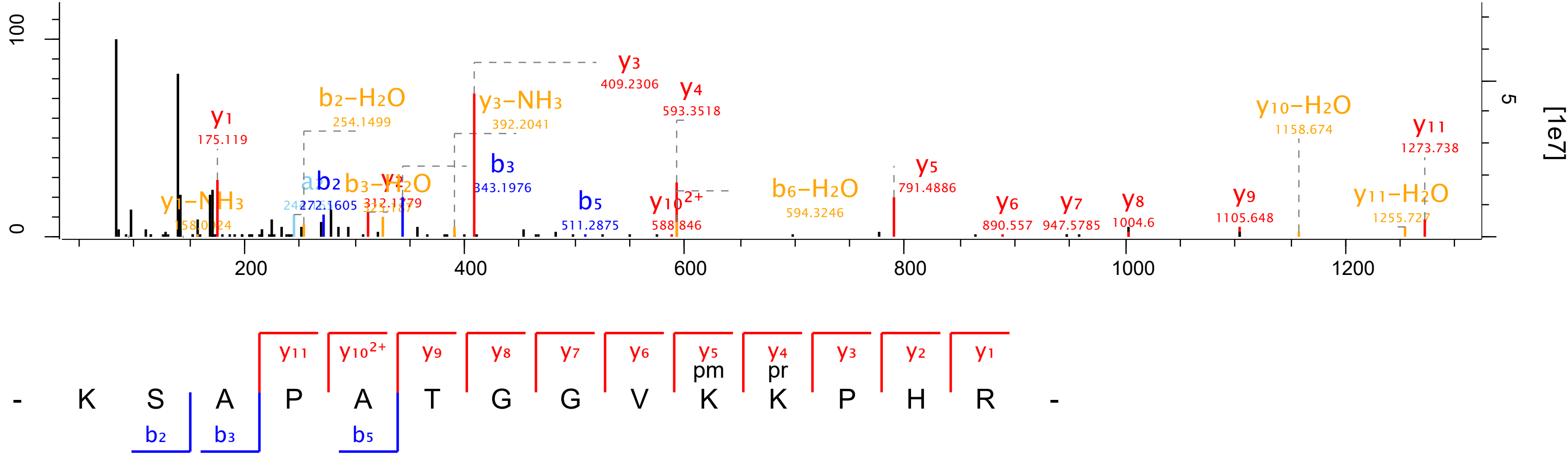

Raw file	Scan	Method	Score	m/z
TP+3	12790	FTMS; HCD	64.22	548.66

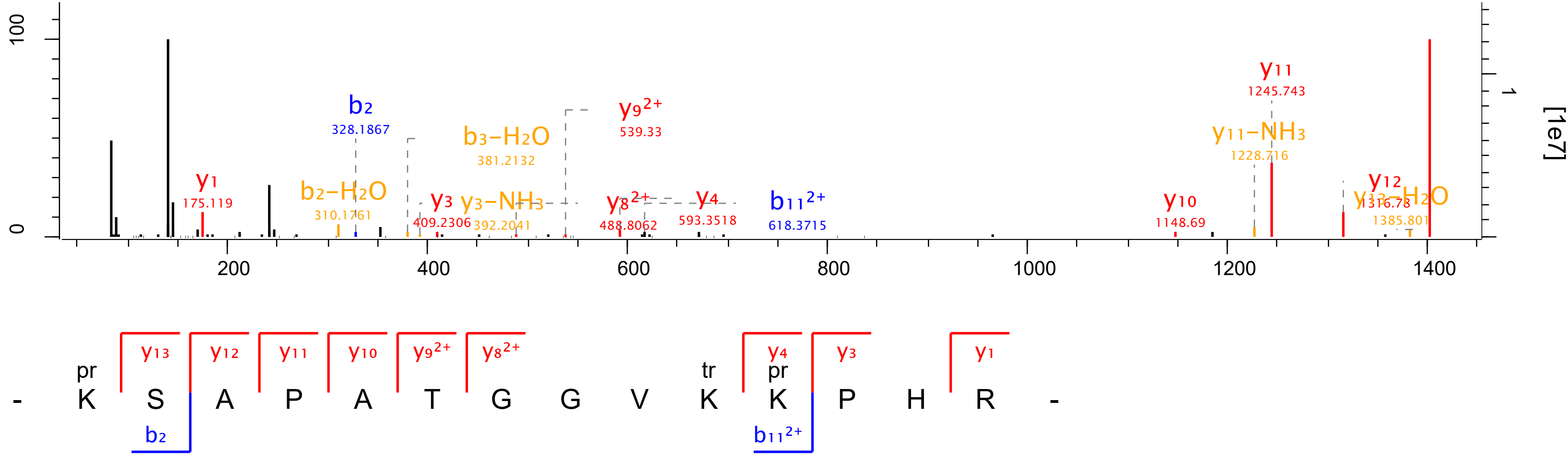

Raw file	Scan	Method	Score	m/z
MR-1	9120	FTMS; HCD	76.73	529.98

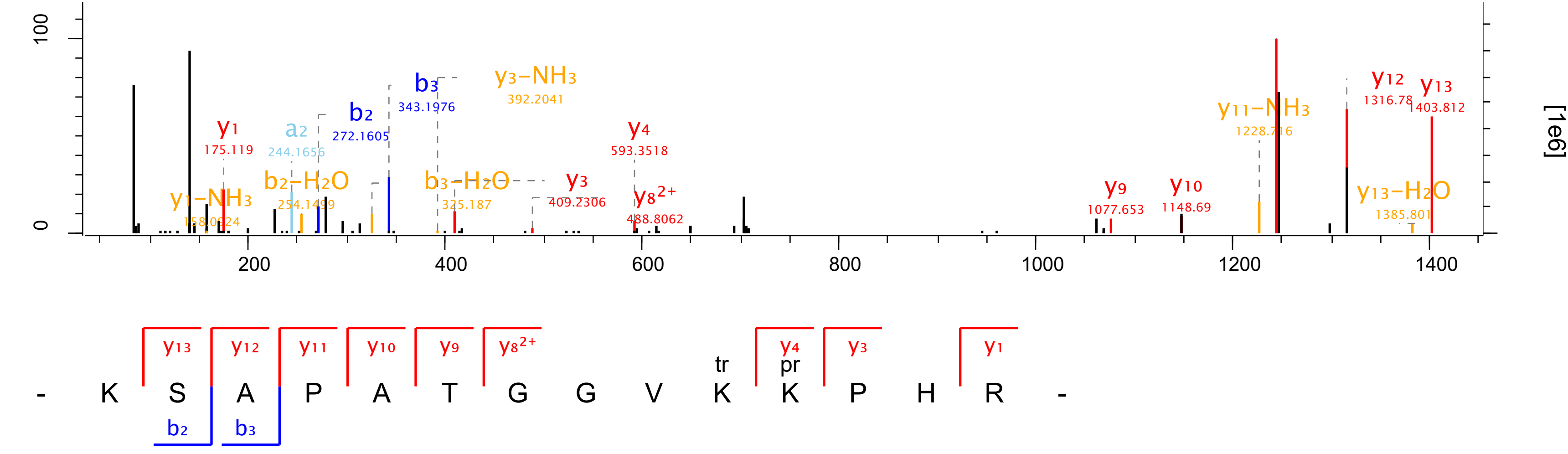

Raw file	Scan	Method	Score	m/z
TP-3	13047	FTMS; HCD	103.83	553.66

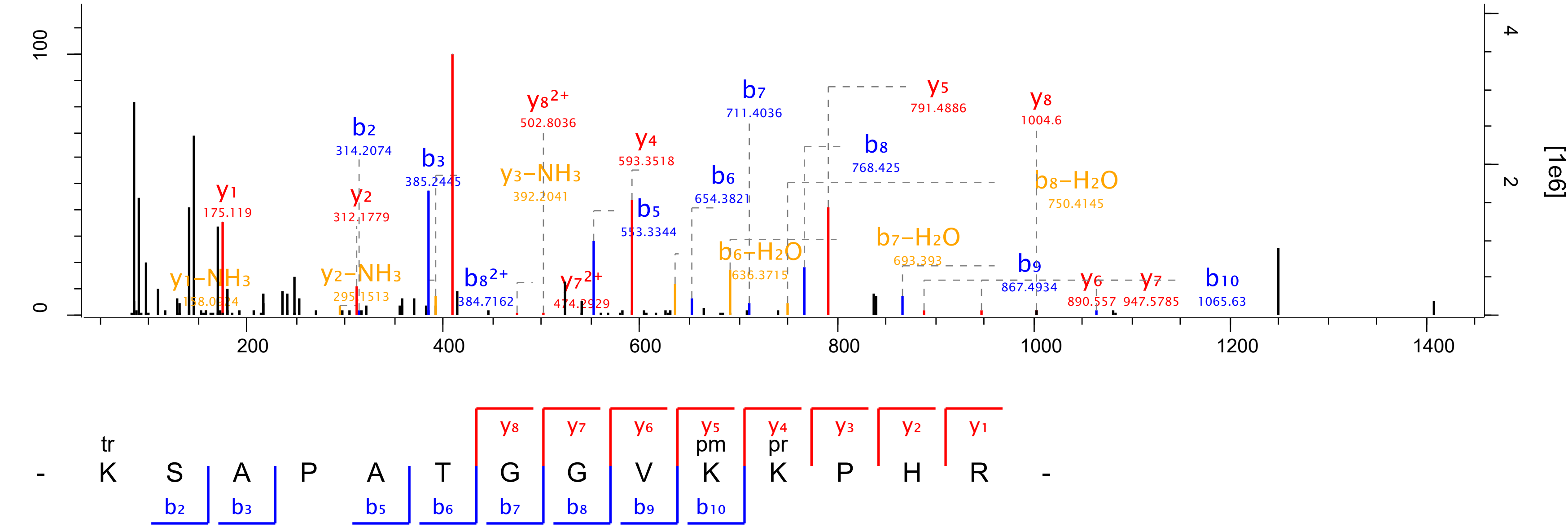

Raw file	Scan	Method	Score	m/z
TR+2	16351	FTMS; HCD	68.17	563.32

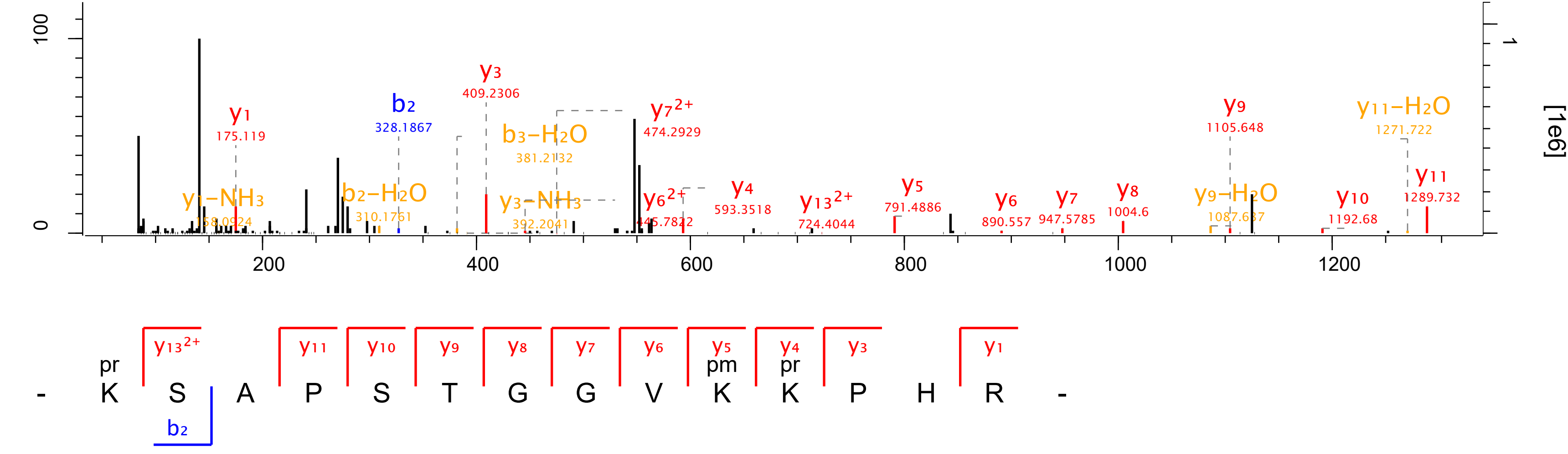

Raw file	Scan	Method	Score	m/z
TP-3	4330	FTMS; HCD	57.27	391.74

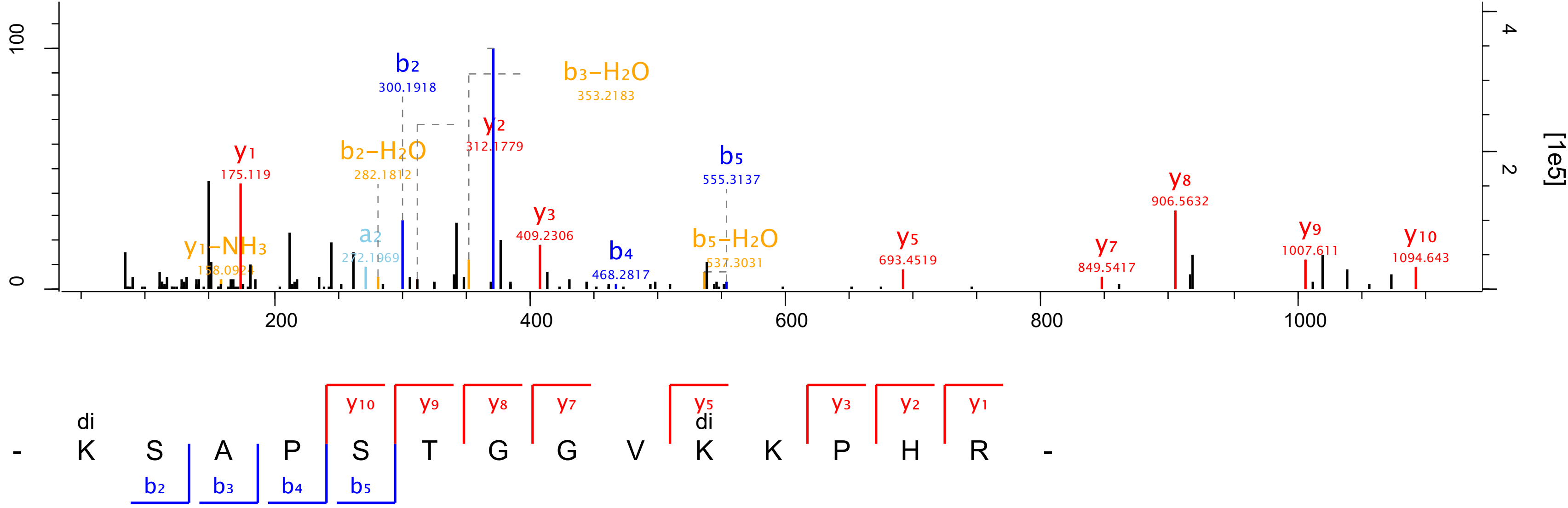

Raw file	Scan	Method	Score	m/z
TP+3	5157	FTMS; HCD	56.57	384.48

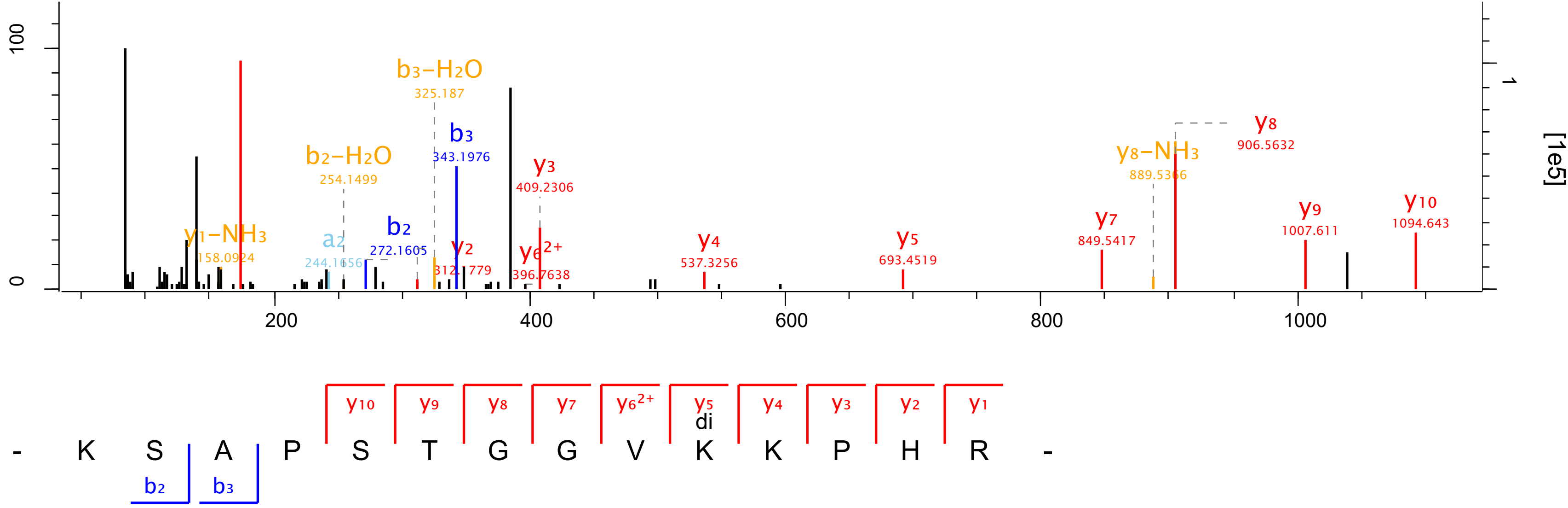

Raw file	Scan	Method	Score	m/z
TP+3	18483	FTMS; HCD	91.62	567.99

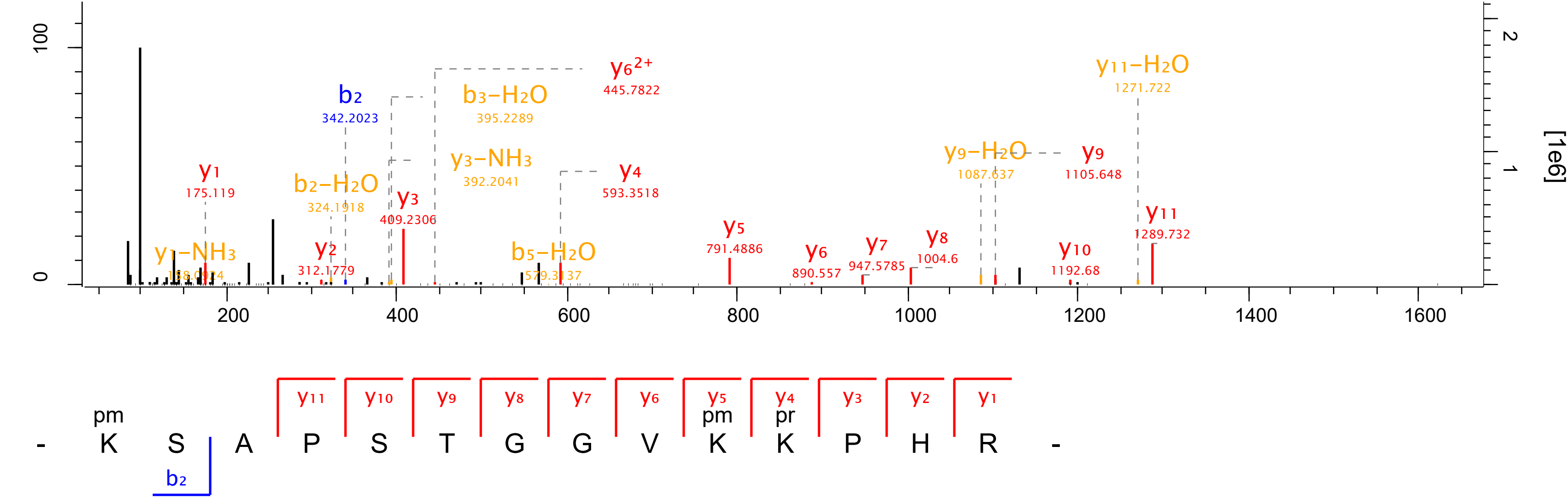

Raw file	Scan	Method	Score	m/z
TP+3	13345	FTMS; HCD	93.77	544.98

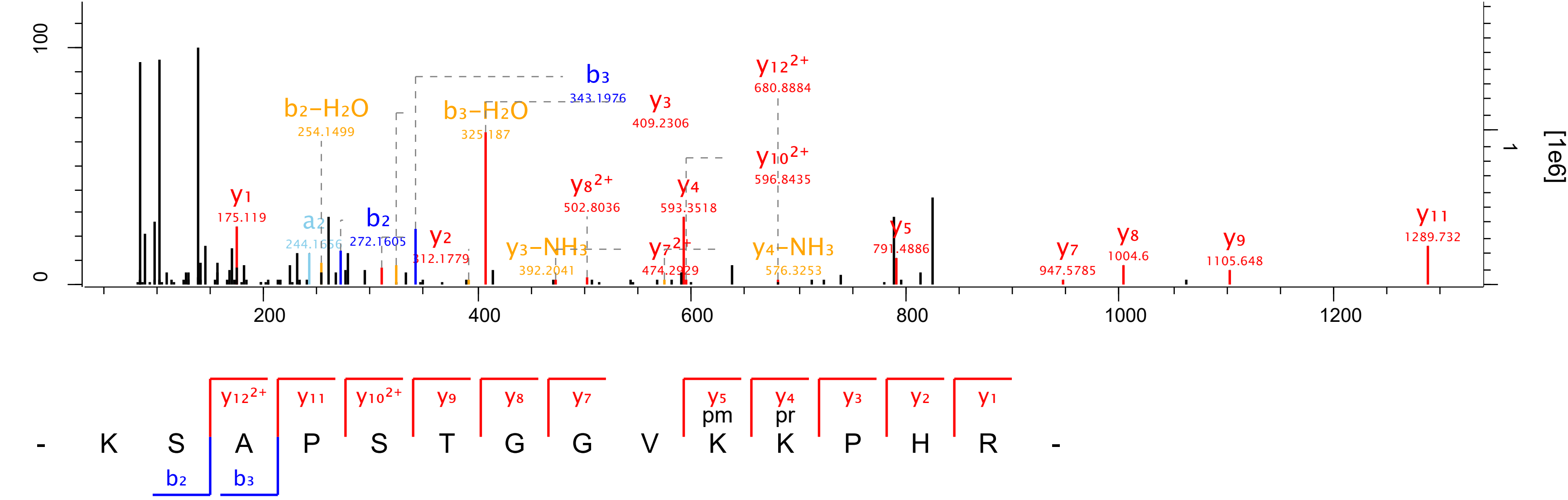

Raw file	Scan	Method	Score	m/z
TP+3	11124	FTMS; HCD	95.26	553.99

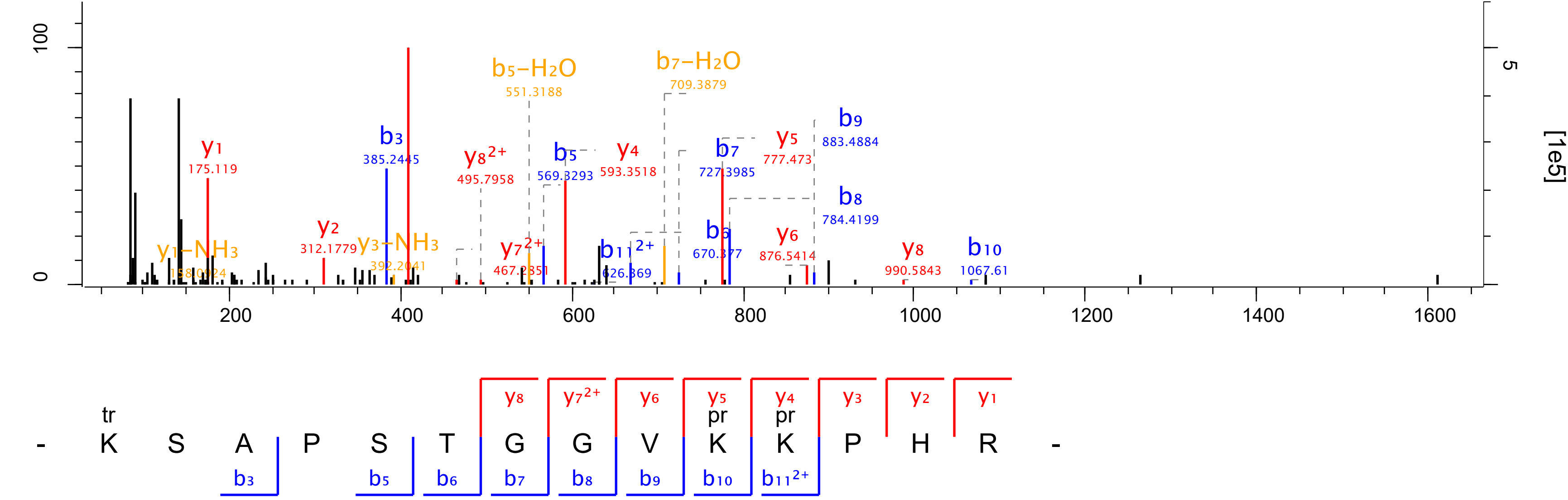

Raw file	Scan	Method	Score	m/z
MR+3	14195	FTMS; HCD	77.78	831.46

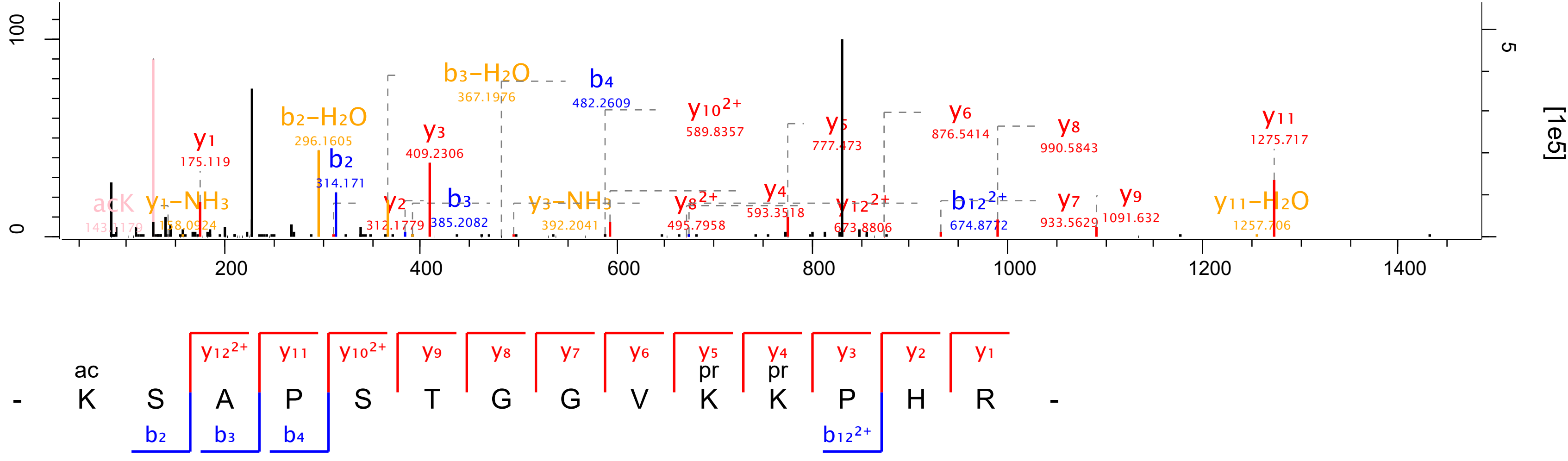

Raw file	Scan	Method	Score	m/z
MR+2	5162	FTMS; HCD	62.53	380.72

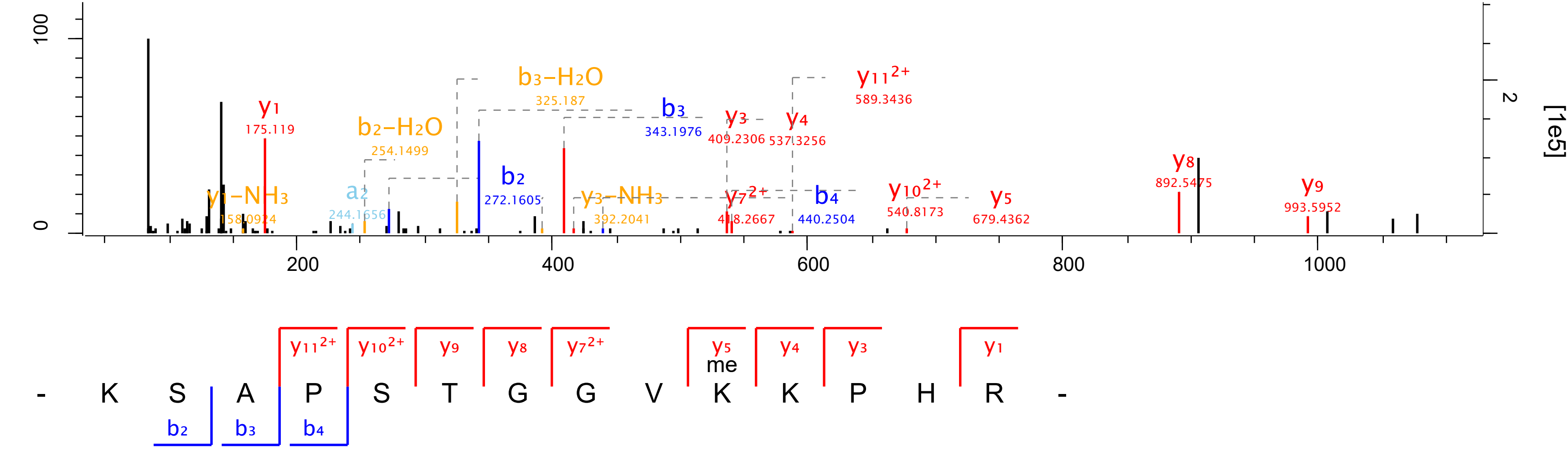

Raw file	Scan	Method	Score	m/z
MP-3	9388	FTMS; HCD	77.89	398.48

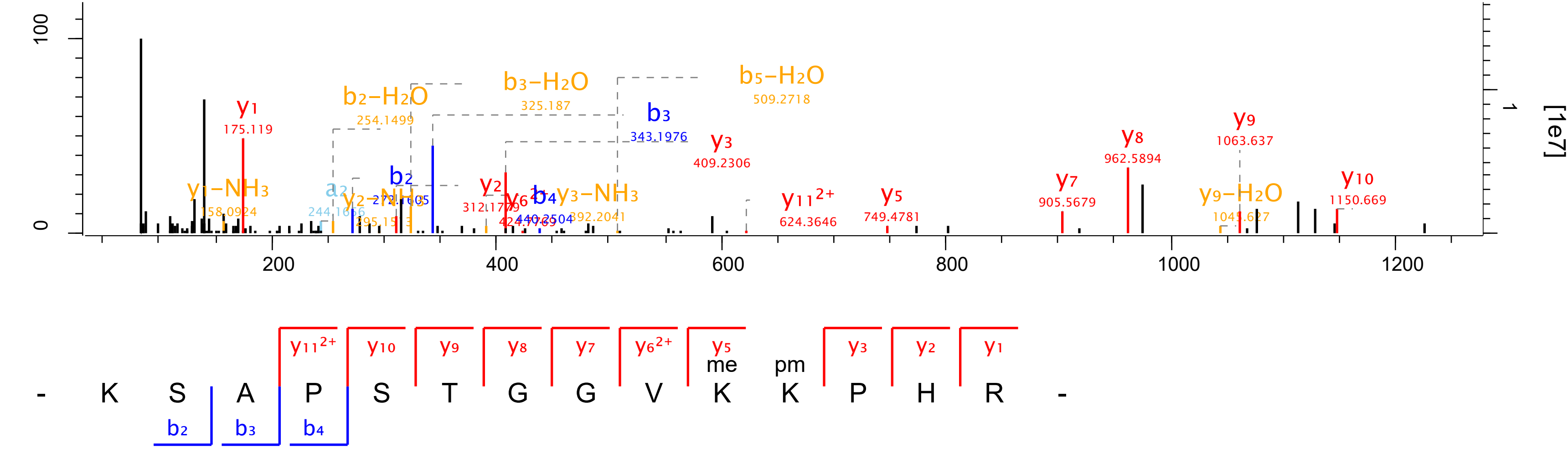

Raw file	Scan	Method	Score	m/z
MP+1	8898	FTMS; HCD	65.9	535.65

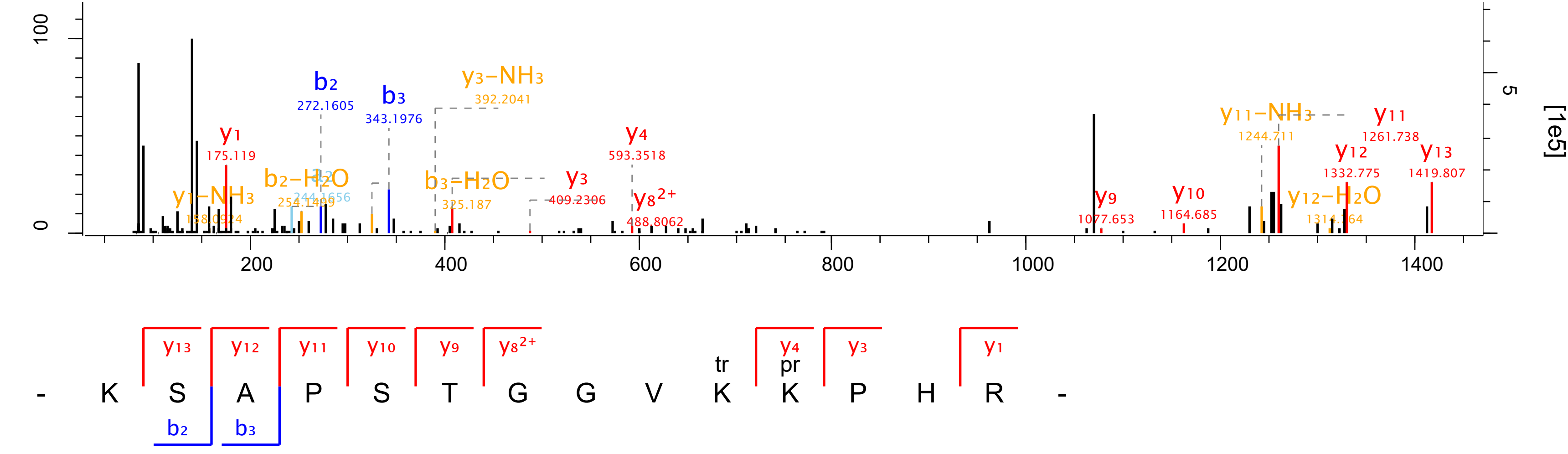

Raw file	Scan	Method	Score	m/z
TP+2	8462	FTMS; HCD	87.26	522.29

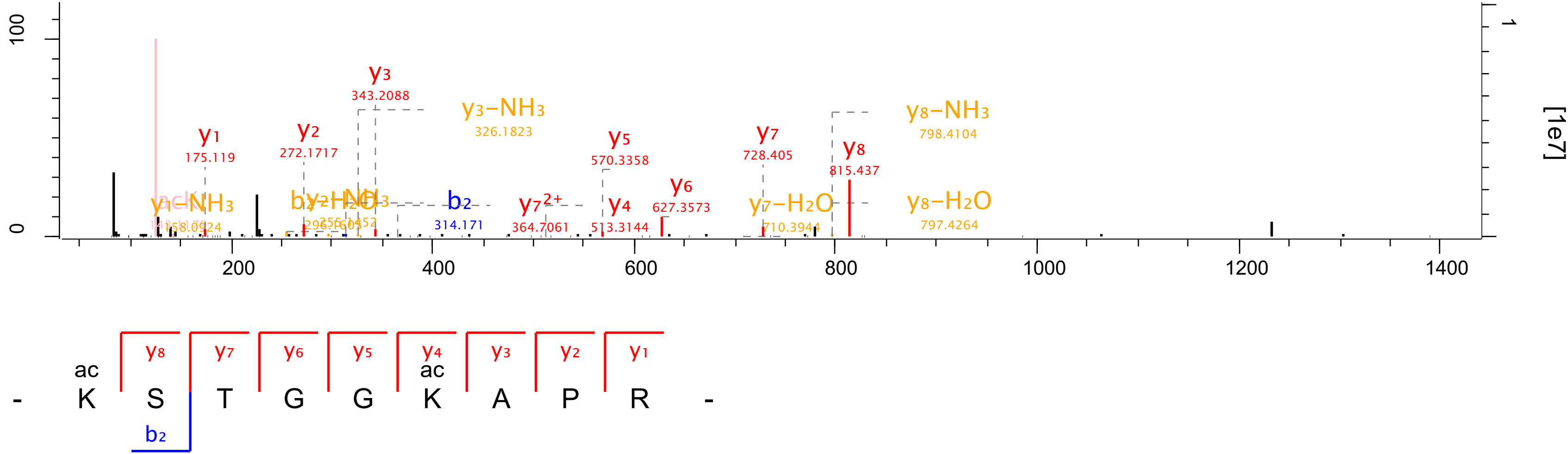

Raw file	Scan	Method	Score	m/z
MP+1	5661	FTMS; HCD	51.28	500.28

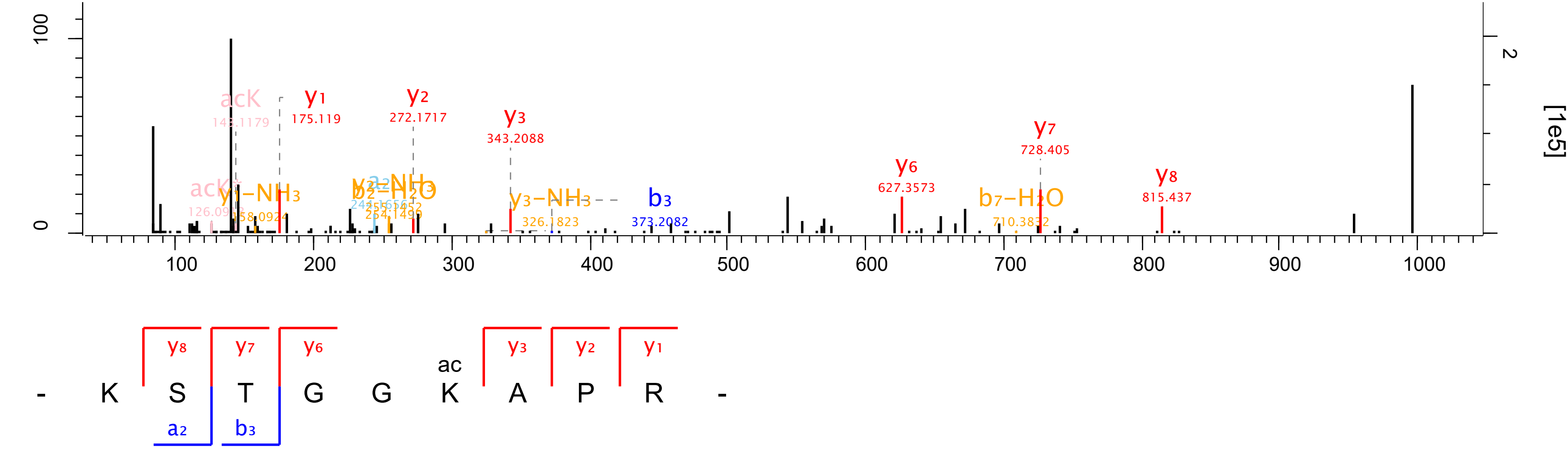

Raw file	Scan	Method	Score	m/z
TP+2	5549	FTMS; HCD	62.47	716.9

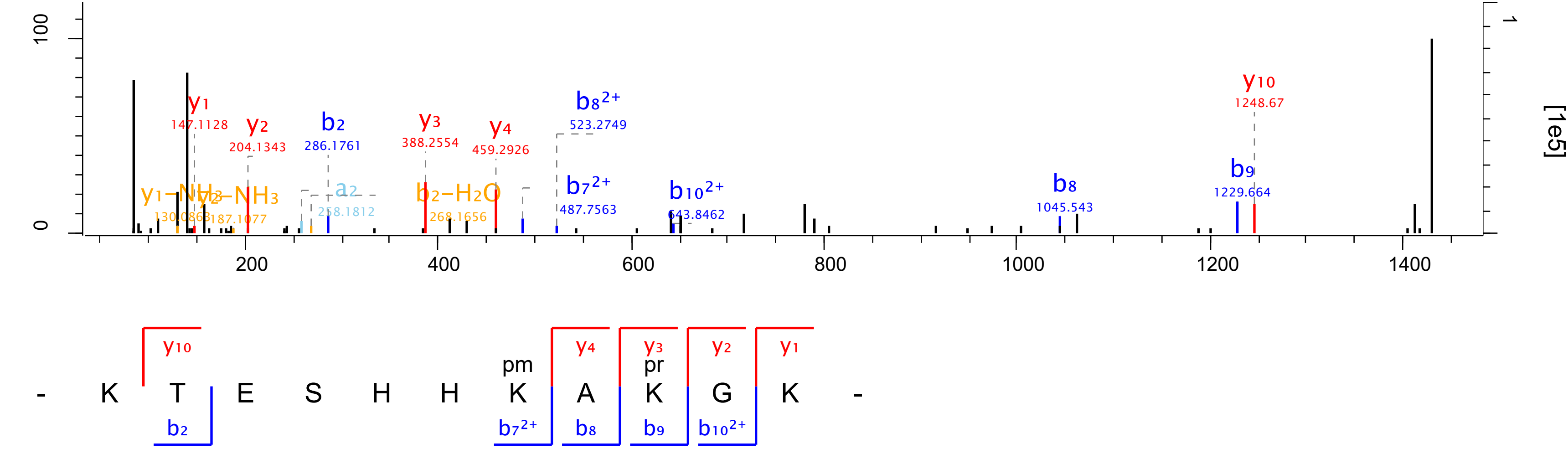

Raw file	Scan	Method	Score	m/z
TR-1	7240	FTMS; HCD	60.36	883.56

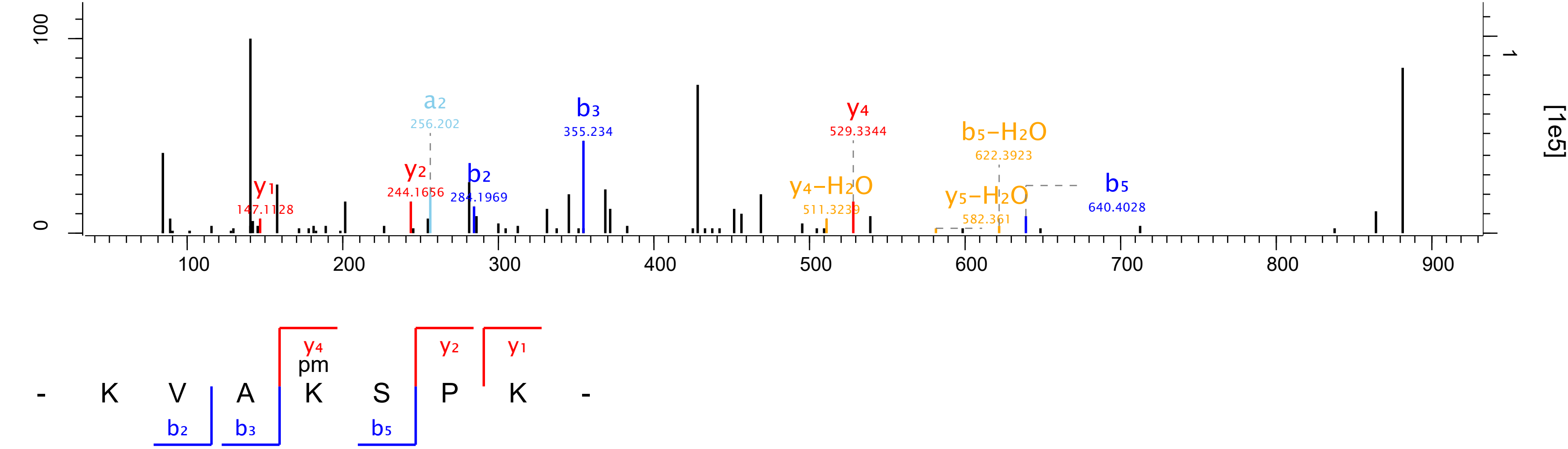

Raw file	Scan	Method	Score	m/z
TP+3	21210	FTMS; HCD	149	636.06

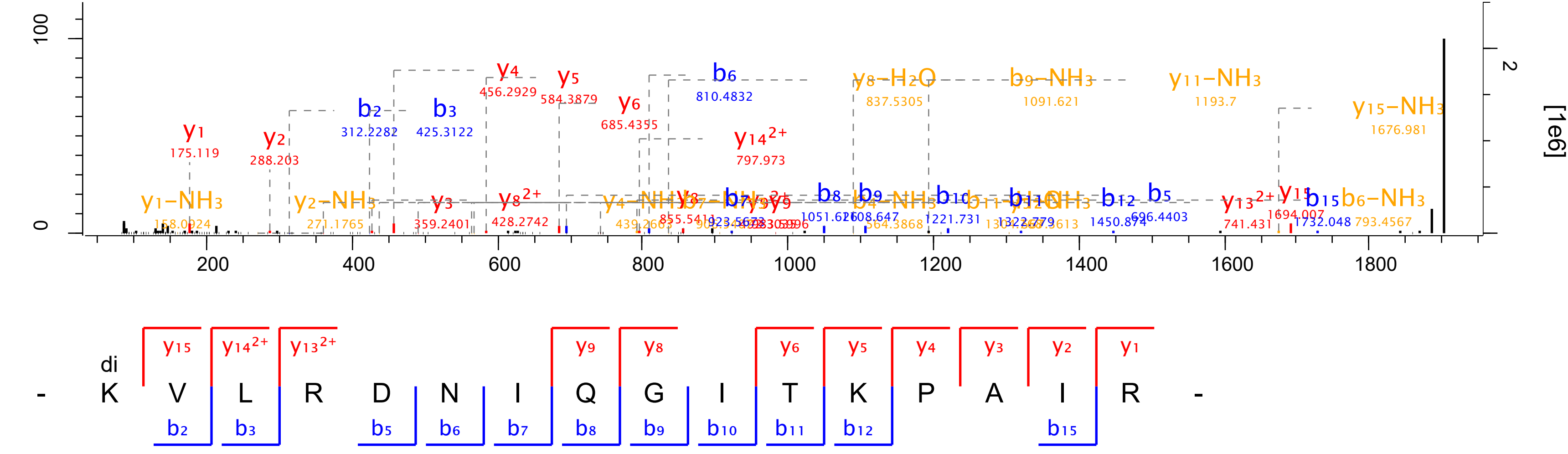

Raw file	Scan	Method	Score	m/z
MP-3	28833	FTMS; HCD	53.56	669.07

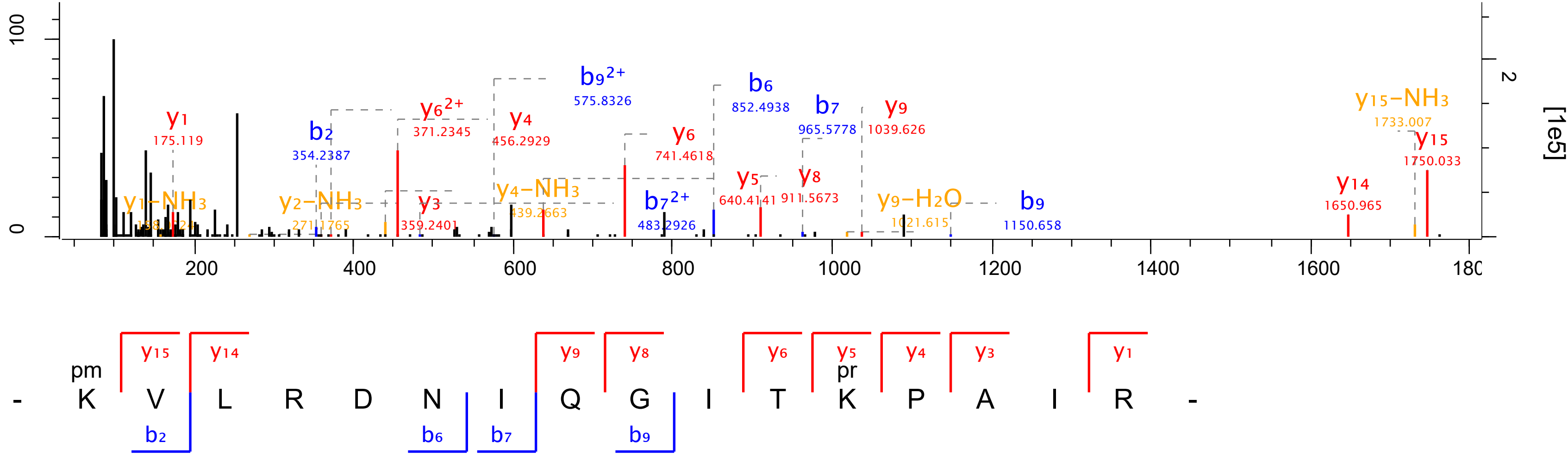

Raw file	Scan	Method	Score	m/z
MR-3	26508	FTMS; HCD	50.83	720.77

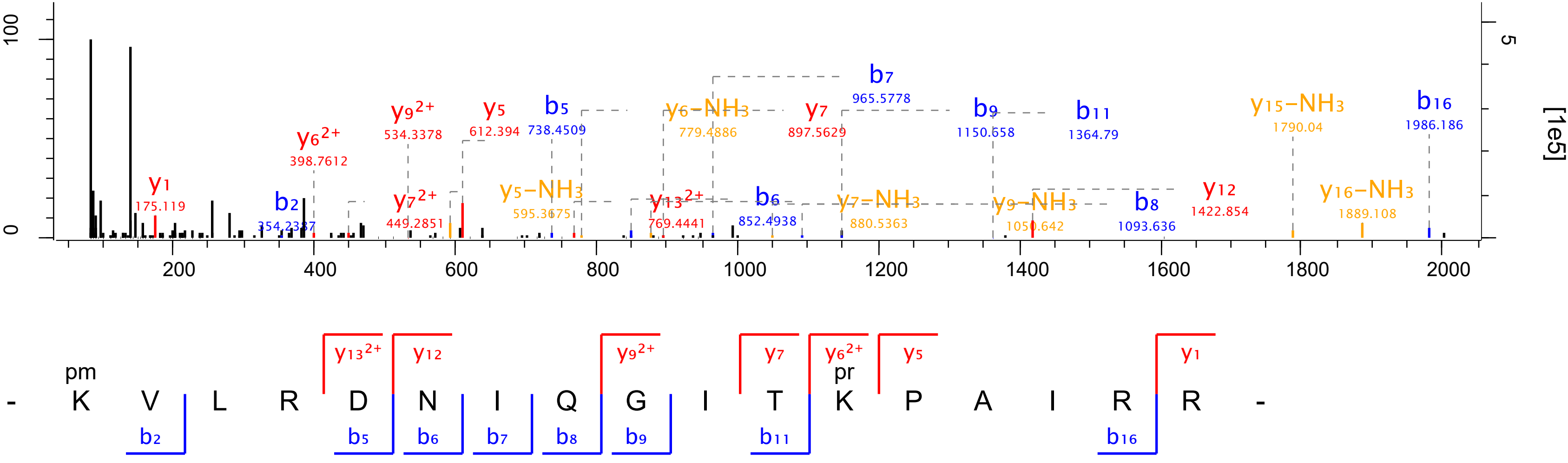

Raw file	Scan	Method	Score	m/z
TP+2	38788	FTMS; HCD	42.35	782.46

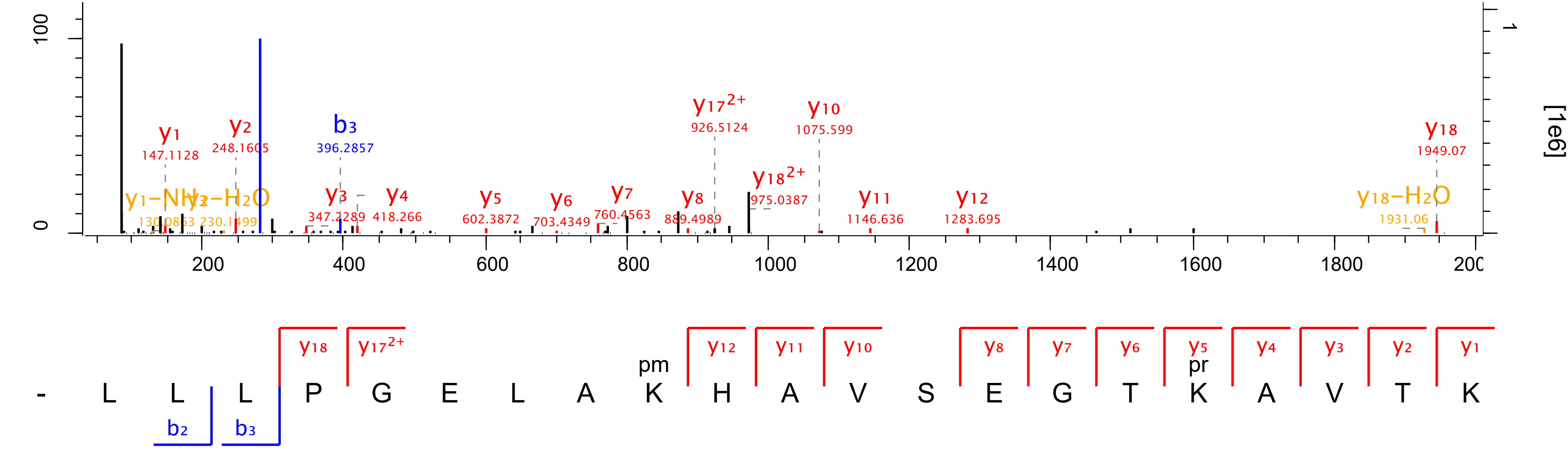

Raw file	Scan	Method	Score	m/z
TP+2	38535	FTMS; HCD	75.96	933.84

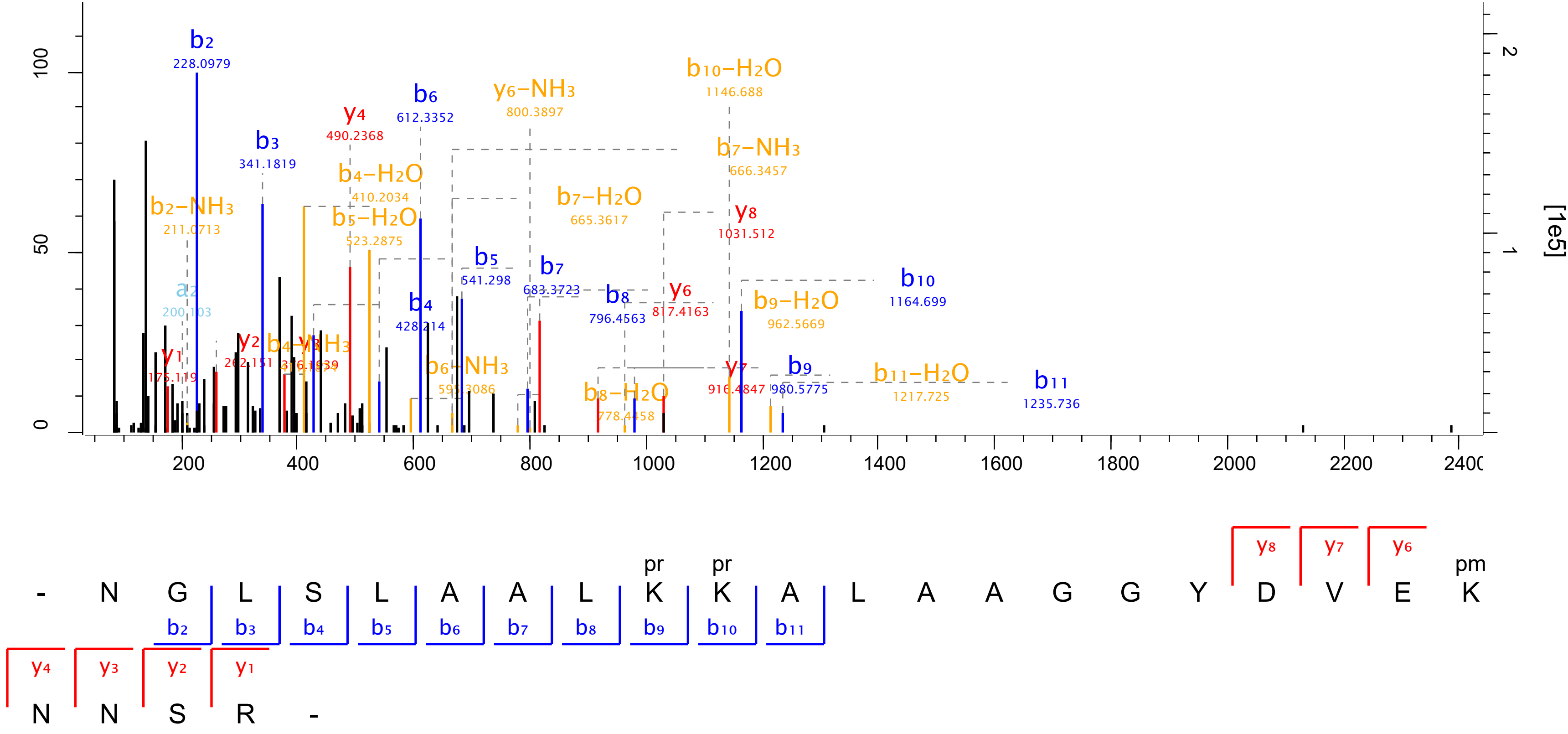

Raw file	Scan	Method	Score	m/z
MP-3	15671	FTMS; HCD	46.89	864.01

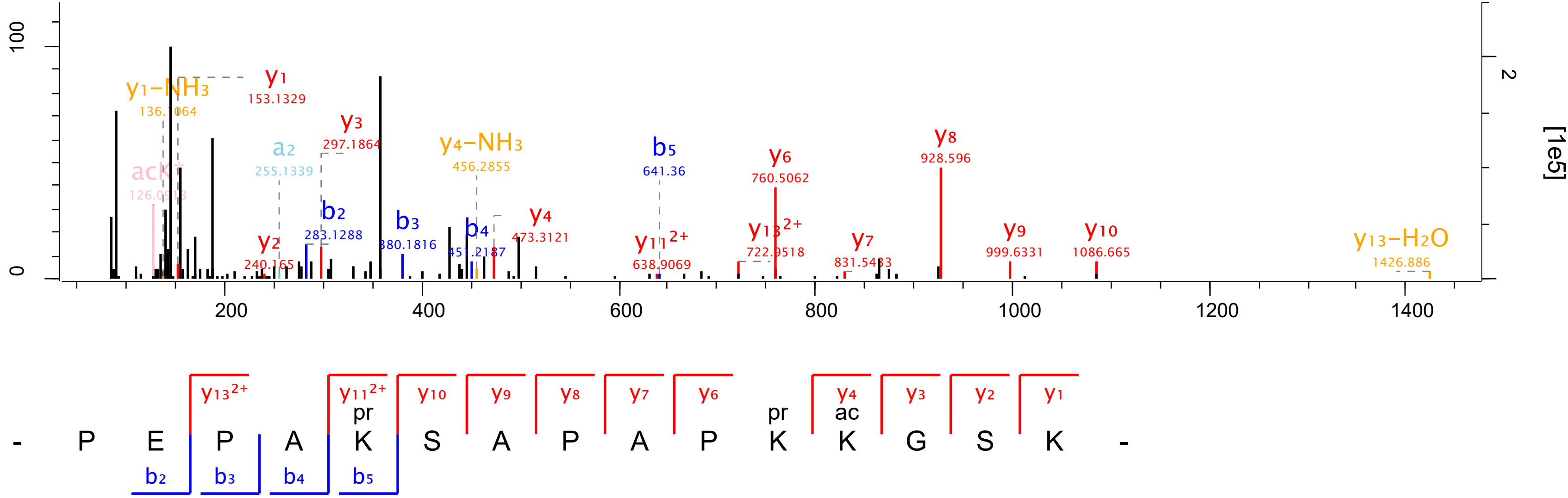

Raw file	Scan	Method	Score	m/z
TR+3	17352	FTMS; HCD	43.79	944.03

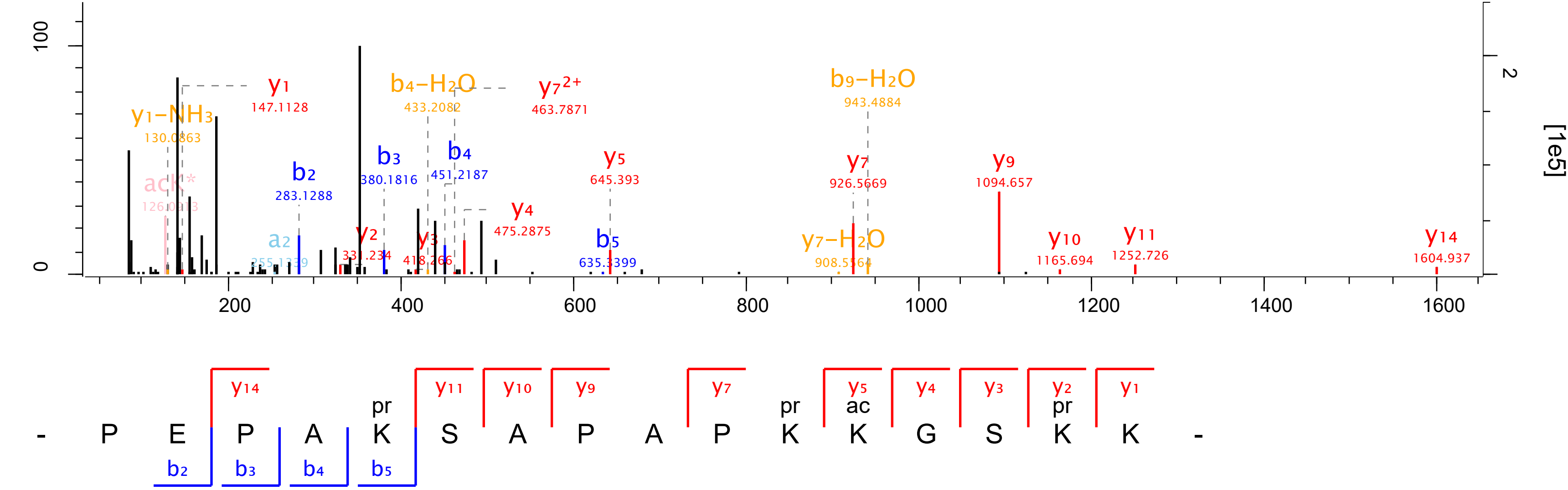

Raw file	Scan	Method	Score	m/z
MP-3	12851	FTMS; HCD	46.46	1220.65

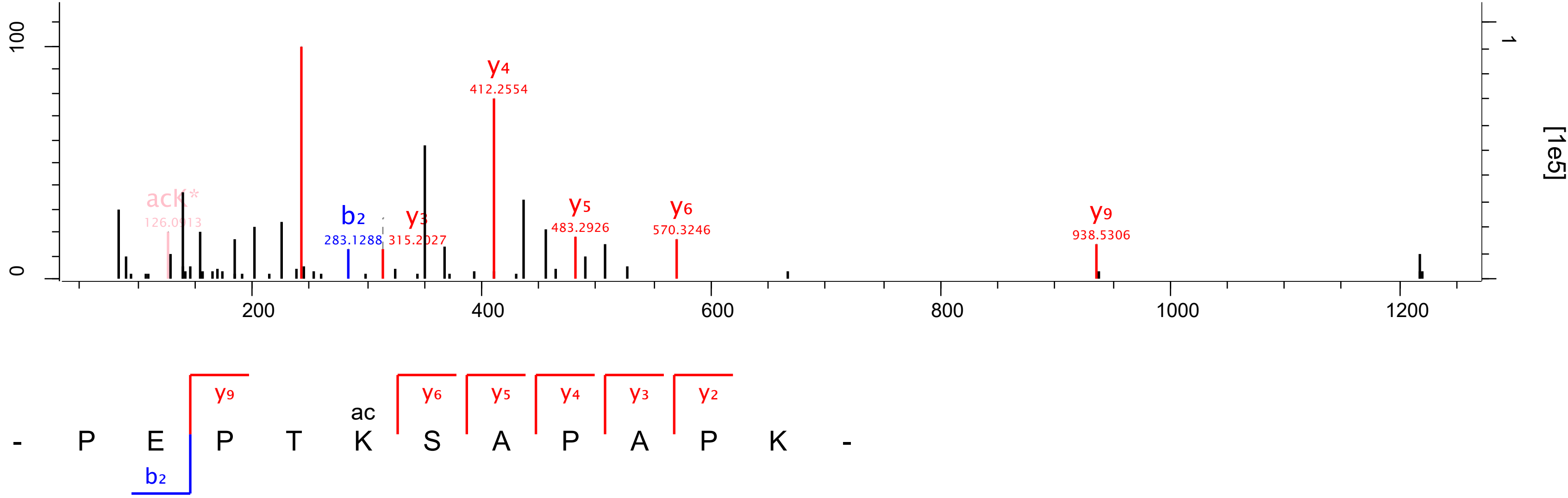

Raw file	Scan	Method	Score	m/z
TP-1	14599	FTMS; HCD	76.06	478.78

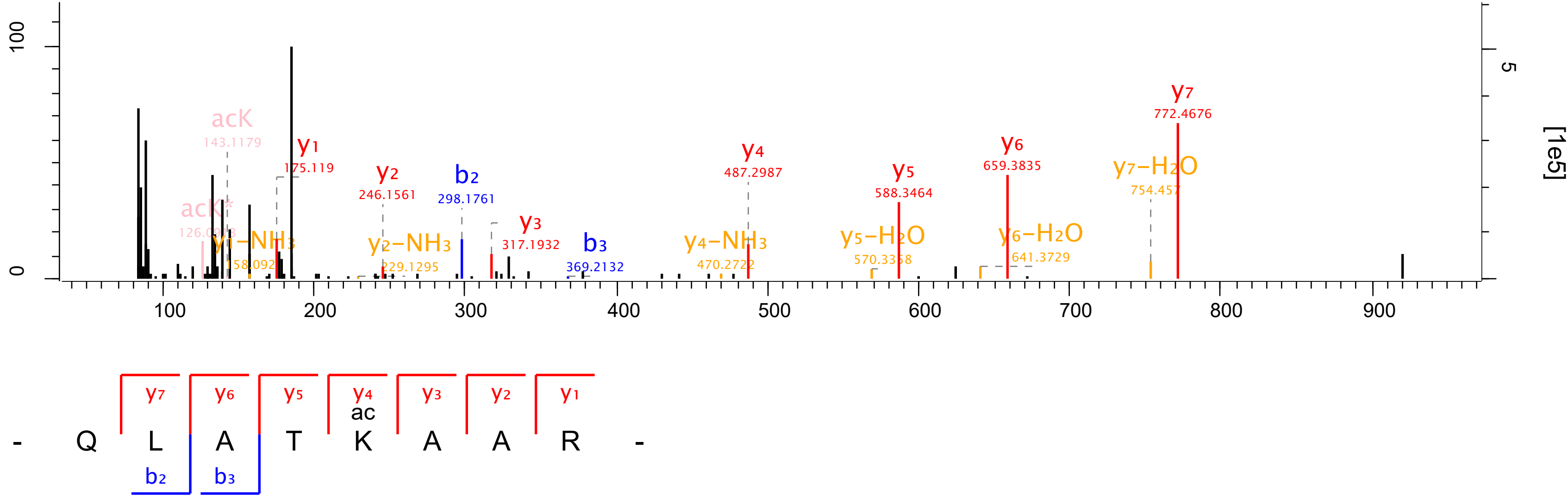

Raw file	Scan	Method	Score	m/z
TP+2	24106	FTMS; HCD	81.92	766.94

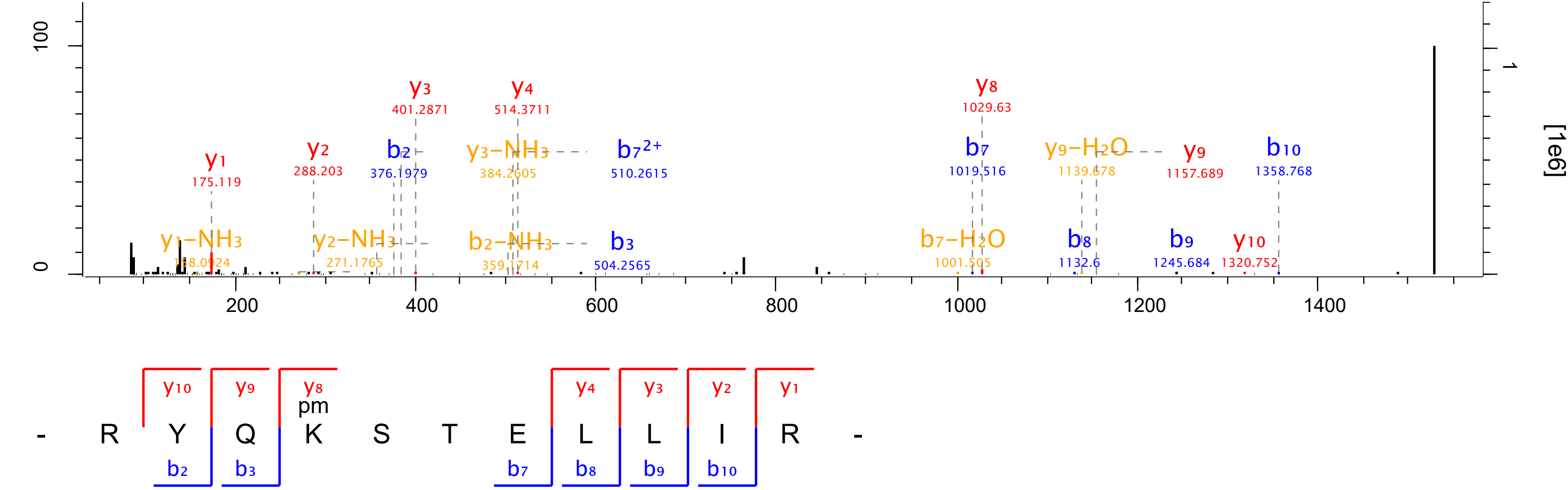

Raw file	Scan	Method	Score	m/z
TR-2	10481	FTMS; HCD	73.93	723.41

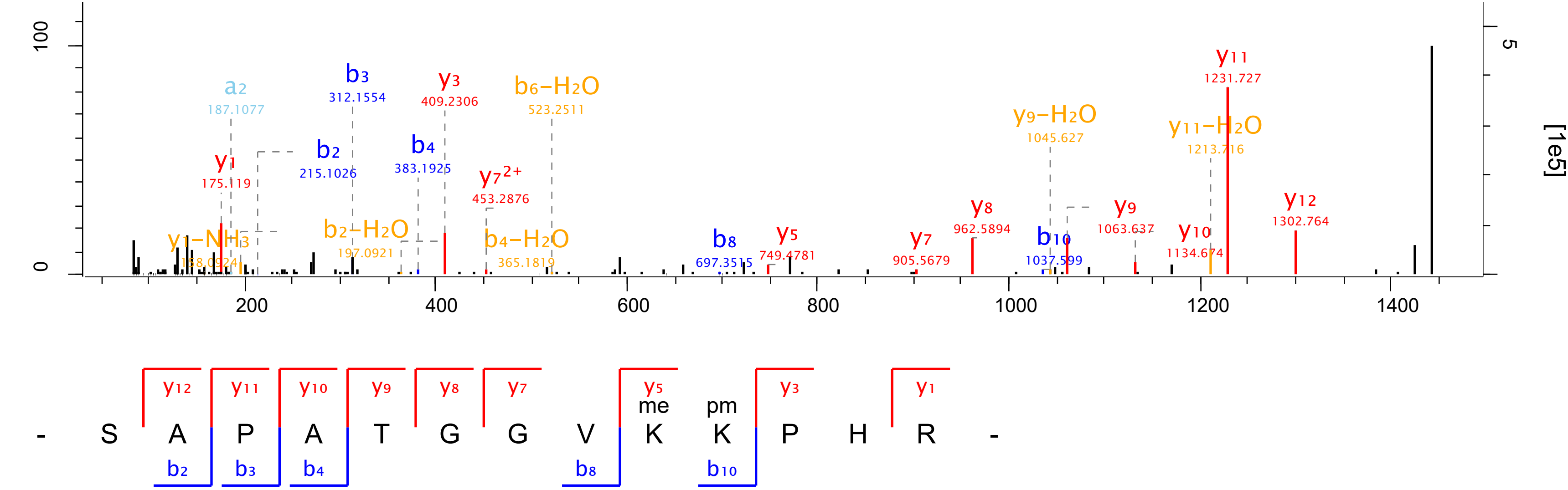

Raw file	Scan	Method	Score	m/z
MP+1	14710	FTMS; HCD	84.36	744.42

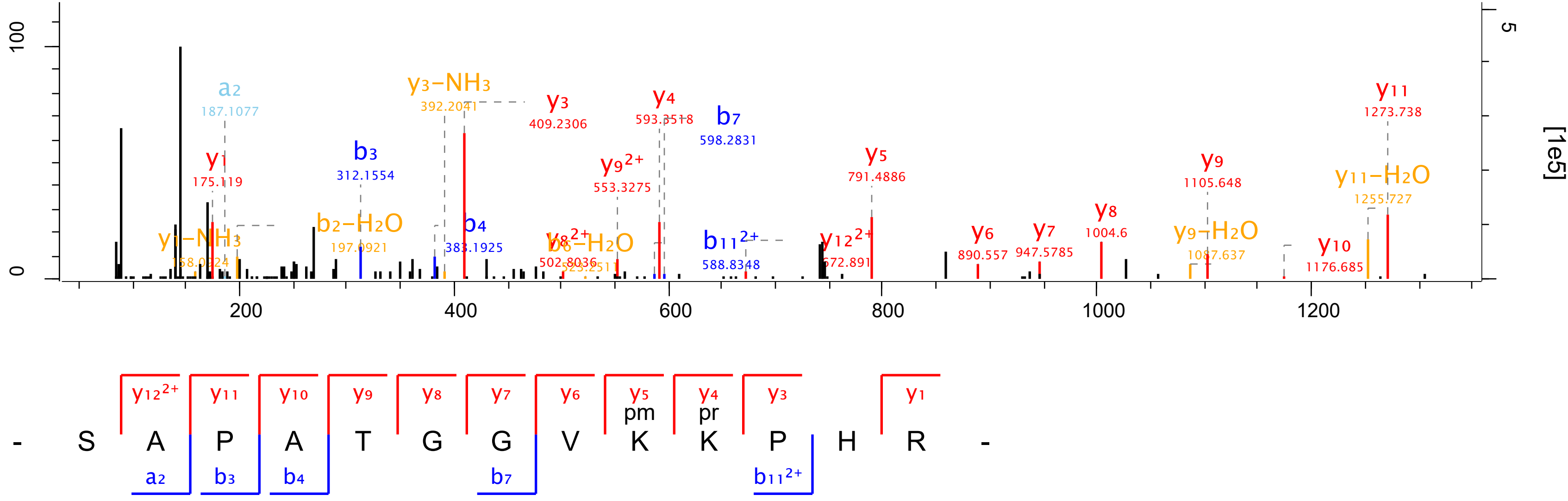

Raw file	Scan	Method	Score	m/z
MR-2	14389	FTMS; HCD	50.19	608.35

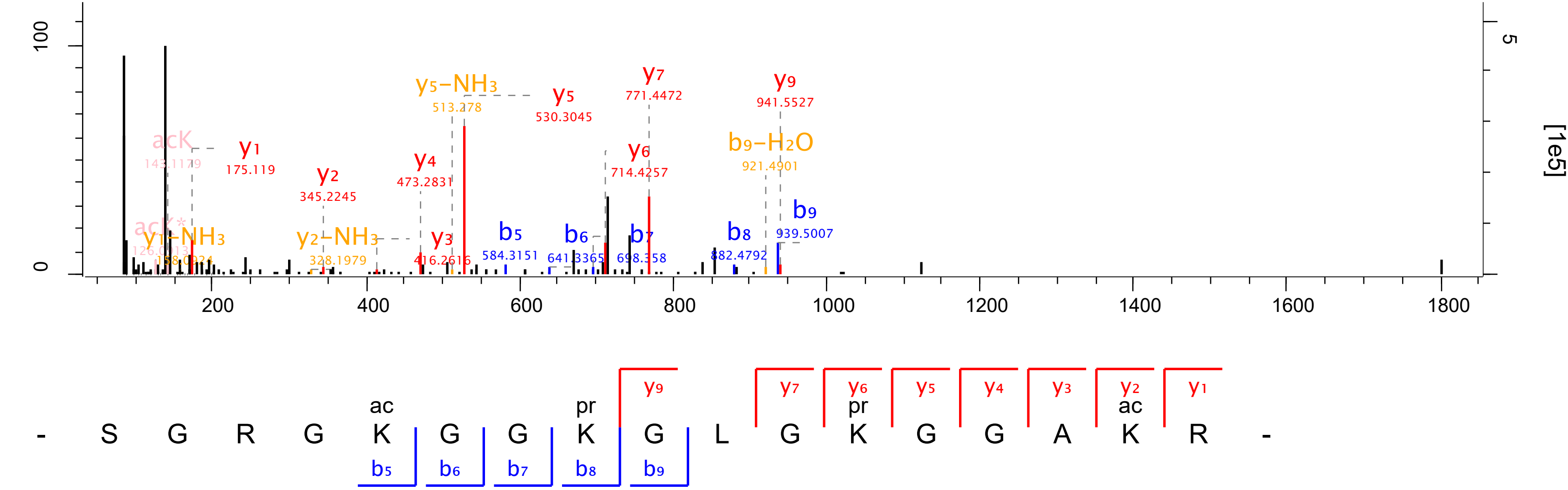

Raw file	Scan	Method	Score	m/z
MR-3	12216	FTMS; HCD	53.36	603.34

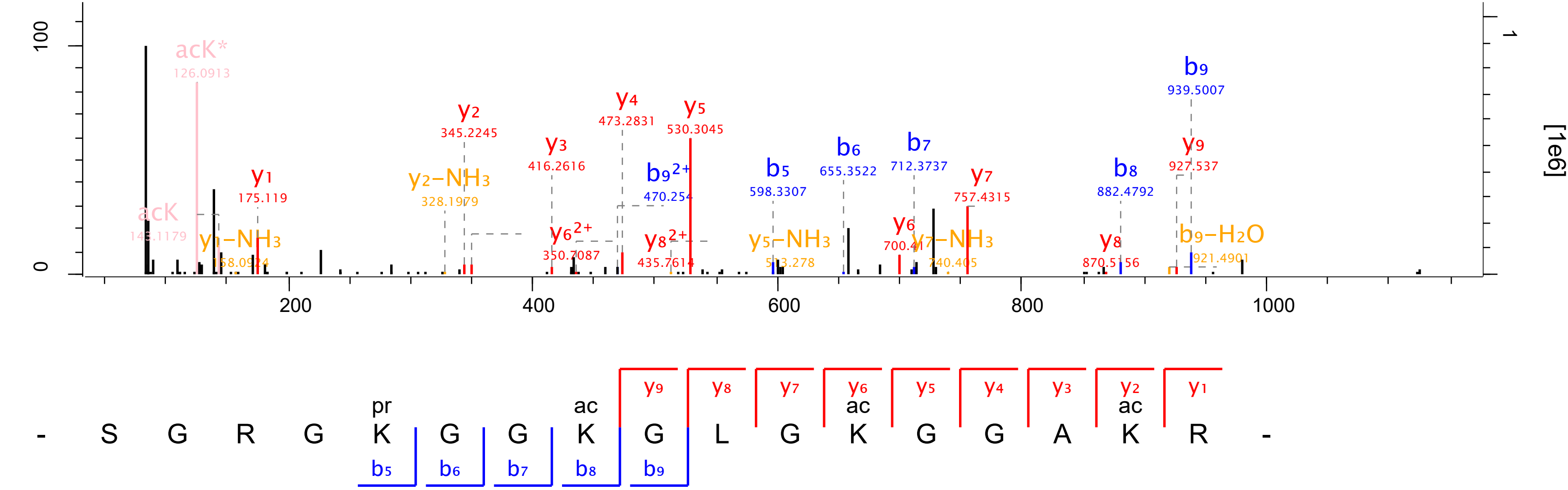

Raw file	Scan	Method	Score	m/z
MP+3	11189	FTMS; HCD	48.37	599

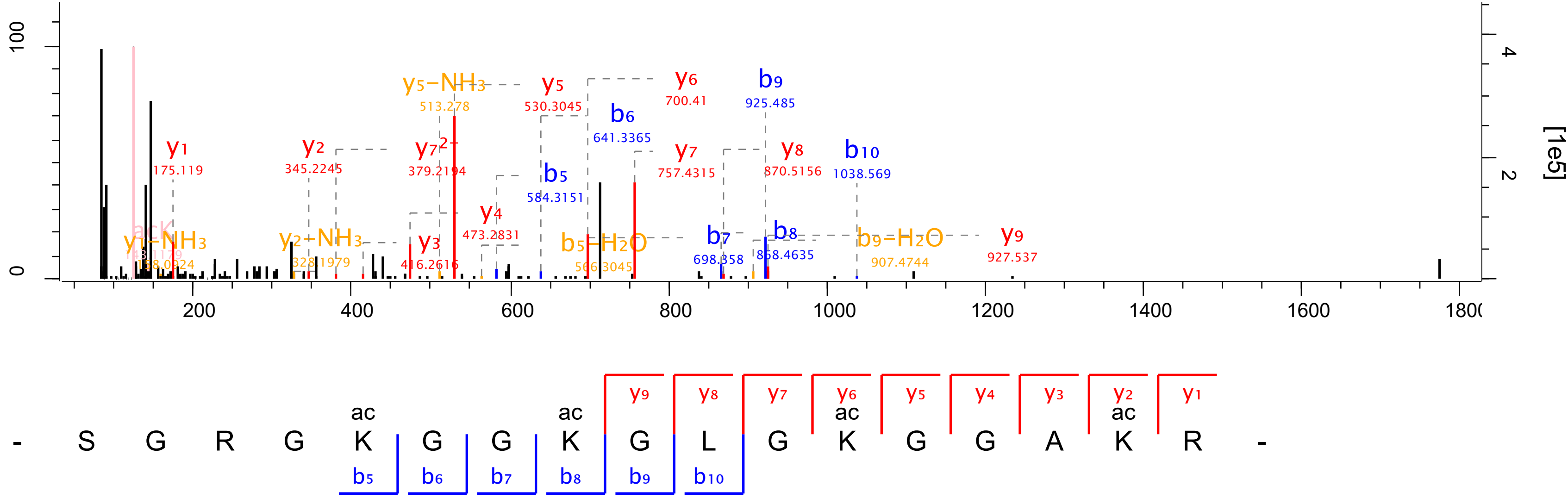

Raw file	Scan	Method	Score	m/z
TR+2	15260	FTMS; HCD	49.54	613.01

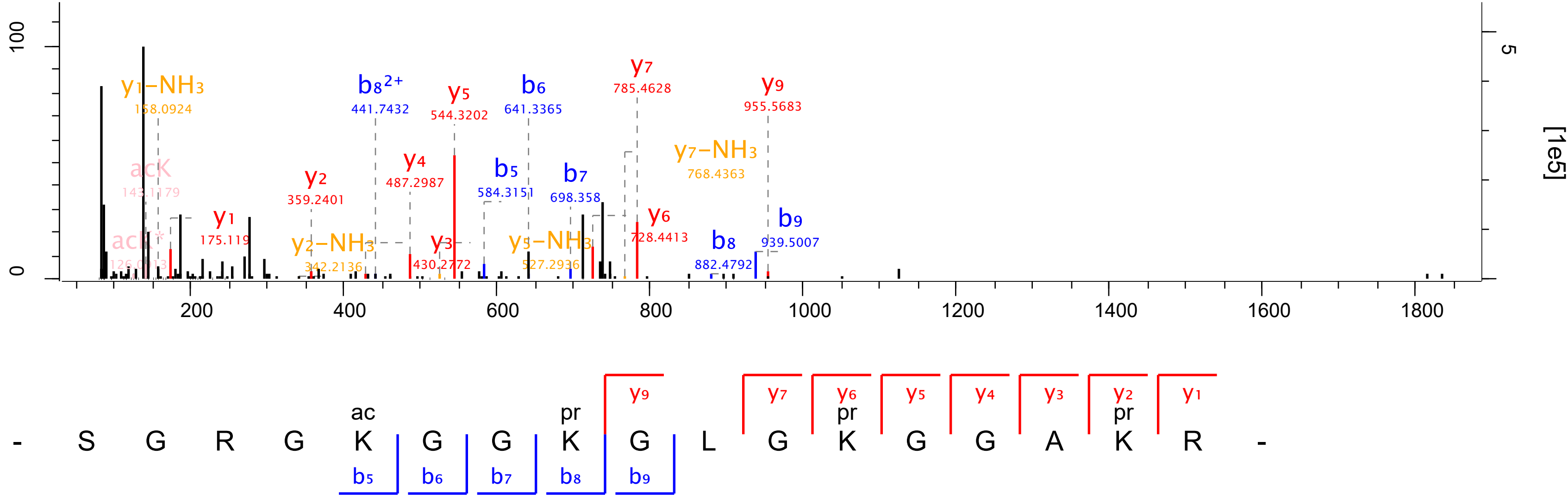

Raw file	Scan	Method	Score	m/z
TP+2	4964	FTMS; HCD	120.57	628.35

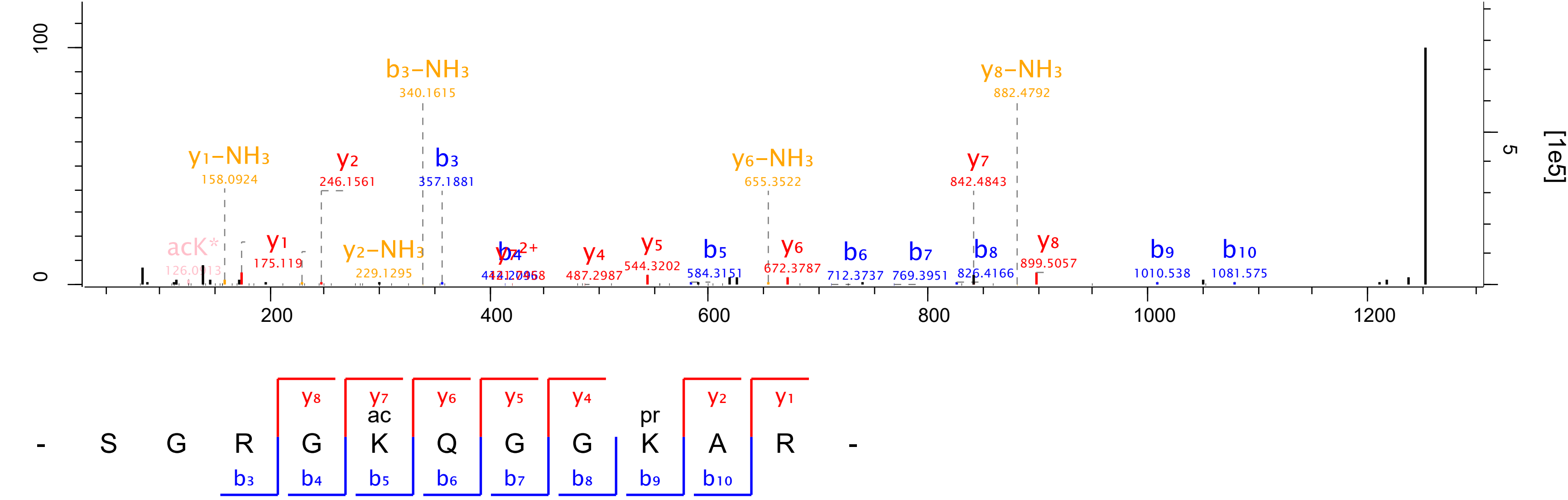

Raw file	Scan	Method	Score	m/z
TR-1	5921	FTMS; HCD	92.19	871.46

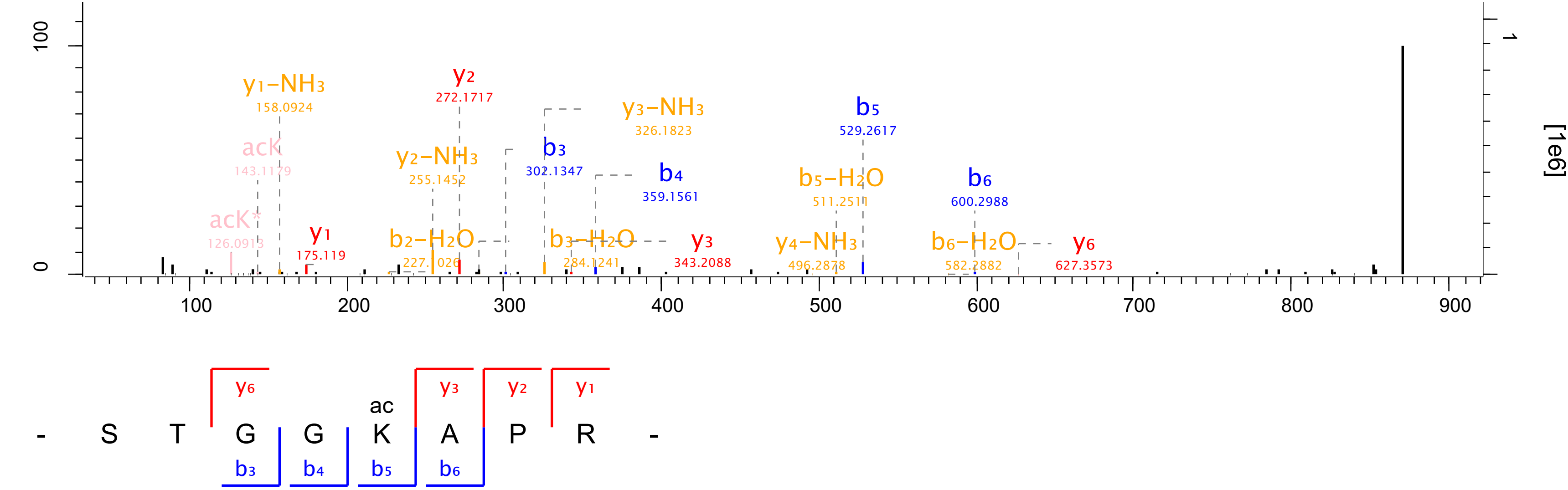

Raw file	Scan	Method	Score	m/z
TP+2	32114	FTMS; HCD	48.57	755.93

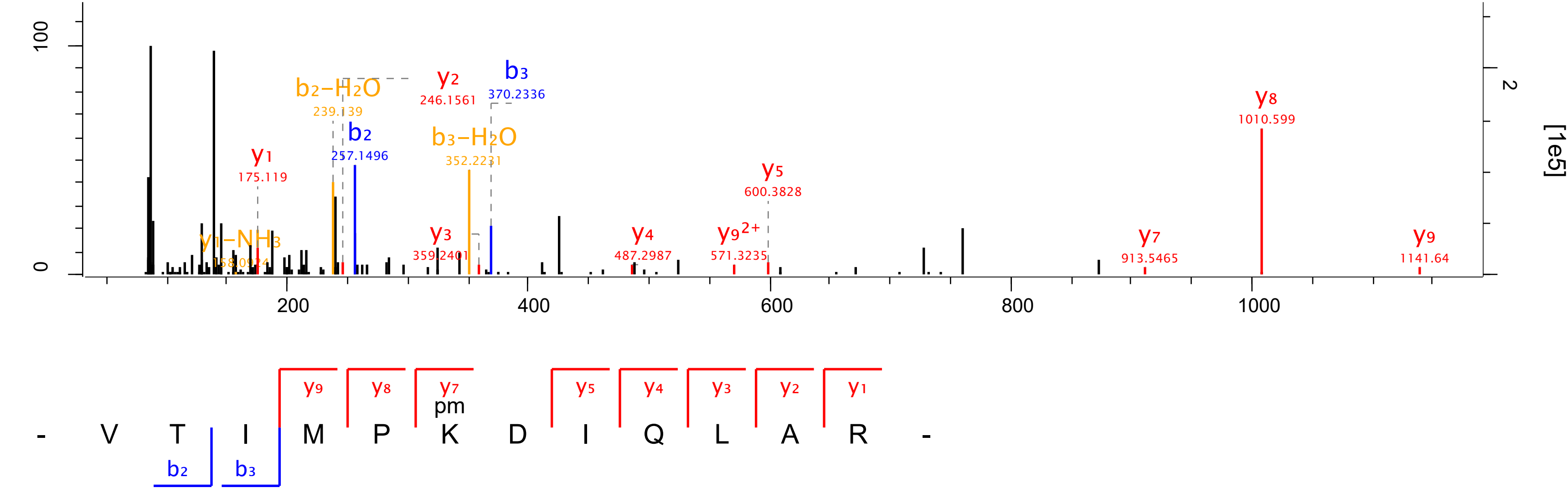

Raw file	Scan	Method	Score	m/z
TR+3	38719	FTMS; HCD	49.59	917.97

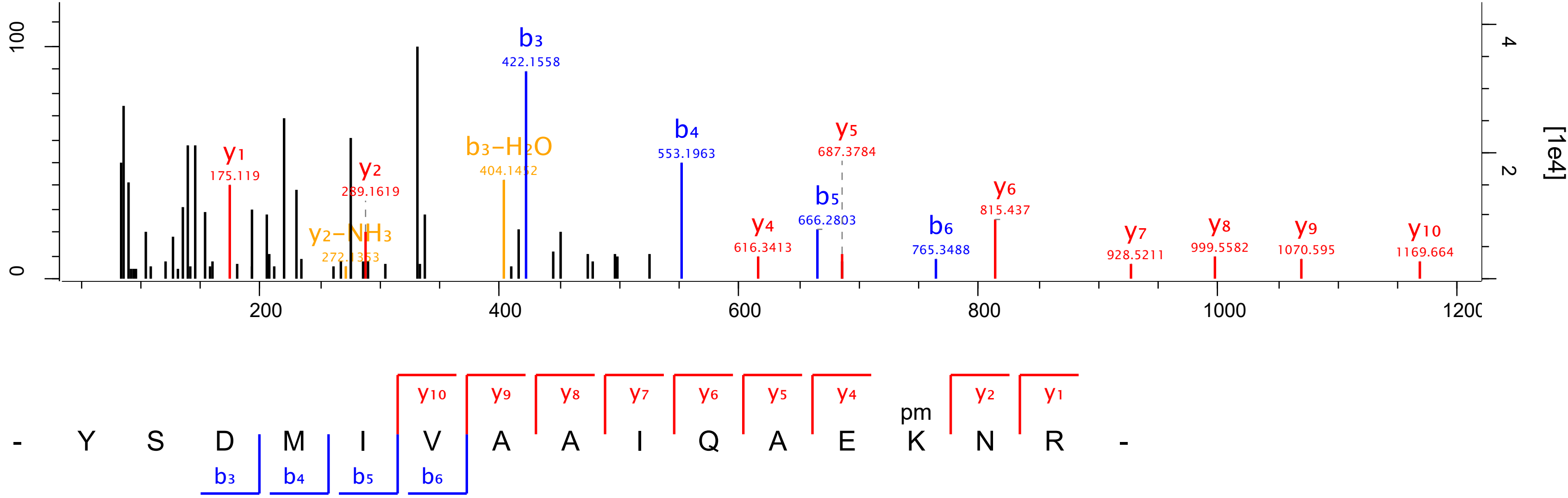

Supplement: Supplementary file 4 — 10.1186/s12014-016-9115-z Tandem spectra of 108 peptide species with PTMs. [file 12014_2016_9115_MOESM4_ESM.pdf]
