# Supplementary figures and images for: Quantitative proteomic analysis of histone modifications in decitabine sensitive and resistant leukemia cell lines
Source: Clin Proteomics. 2016 Jul 5;13:14. doi: 10.1186/s12014-016-9115-z (PMC4932764; doi:10.1186/s12014-016-9115-z)

## Slide 1
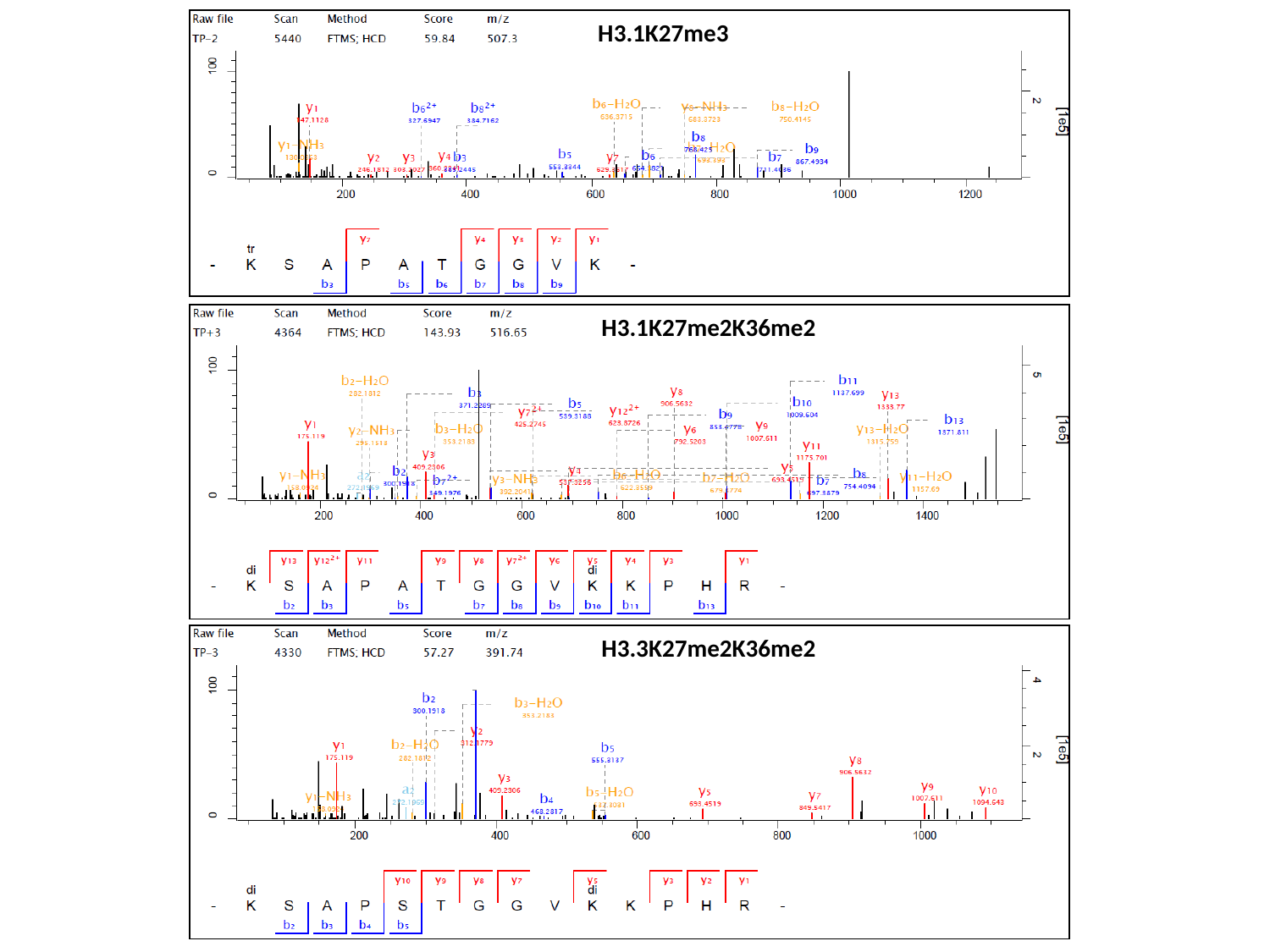

H3.1K27me3
H3.1K27me2K36me2
H3.3K27me2K36me2

## Slide 2
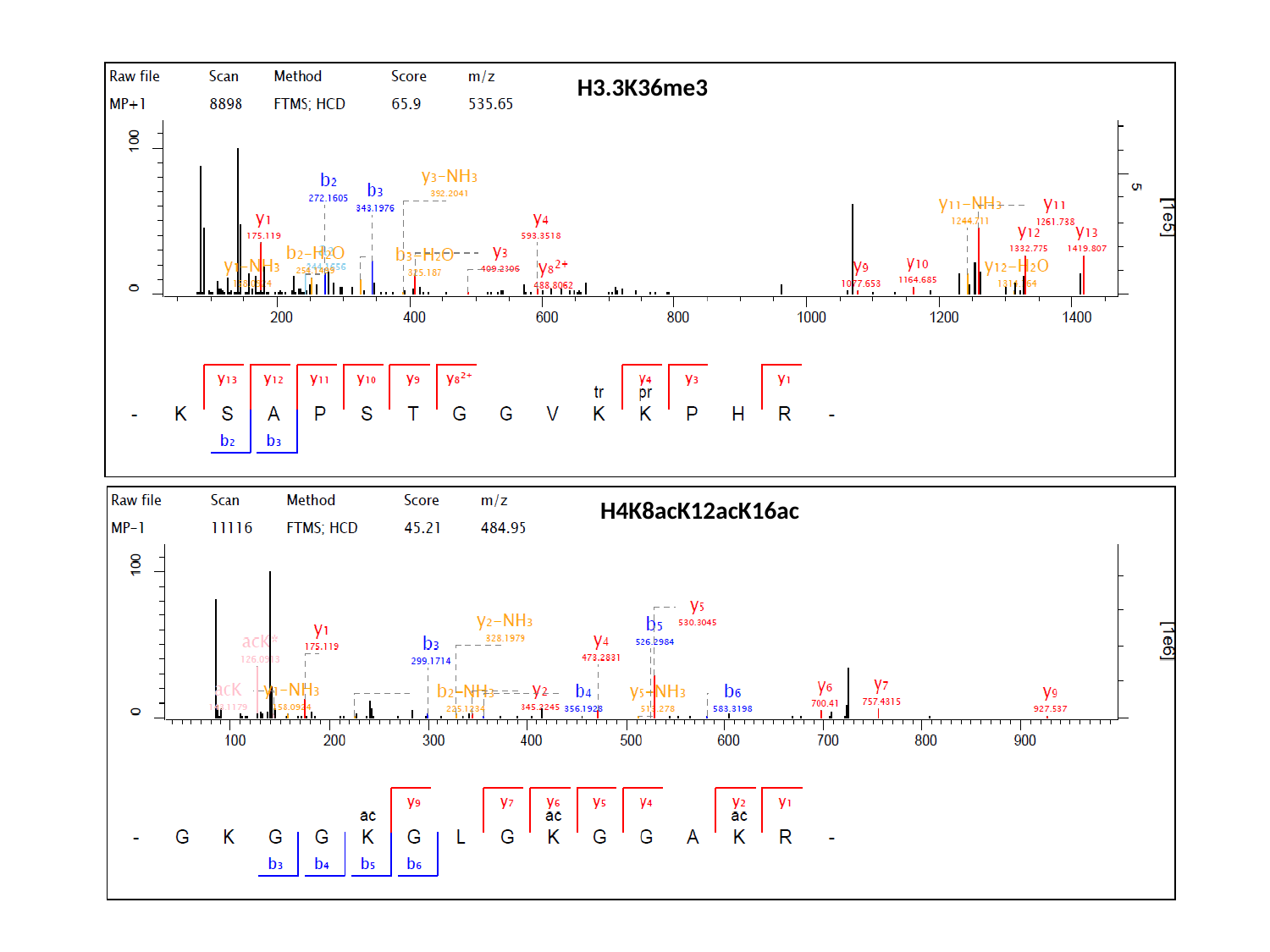

H3.3K36me3
H4K8acK12acK16ac

## Slide 3
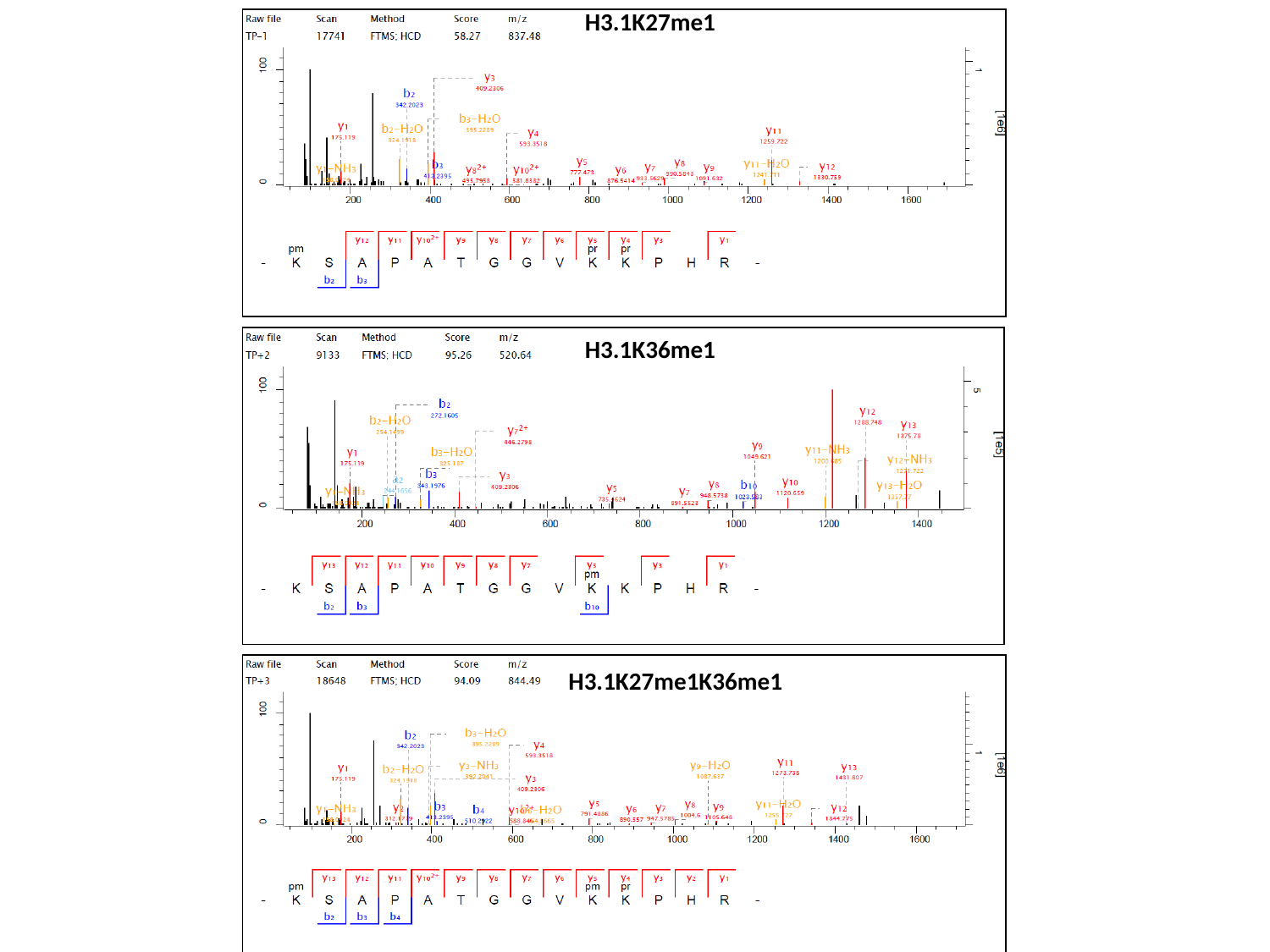

H3.1K27me1
H3.1K36me1
H3.1K27me1K36me1

Supplement: Supplementary file 6 — 10.1186/s12014-016-9115-z A list of 107 modified peptide species. Note: Trypsin cleaves K if propionylation is incomplete. [file 12014_2016_9115_MOESM6_ESM.pptx]
